# Unravelling the enigma of lignin^OX^: can the oxidation of lignin be controlled?†
†Electronic supplementary information (ESI) available: The raw data files can be found at DOI: 10.17630/71024a95-c166-45f5-8537-712ae2d015c4. See DOI: 10.1039/c7sc03520a


**DOI:** 10.1039/c7sc03520a

**Published:** 2017-11-09

**Authors:** Haiwei Guo, Daniel M. Miles-Barrett, Andrew R. Neal, Tao Zhang, Changzhi Li, Nicholas J. Westwood

**Affiliations:** a School of Chemistry and Biomedical Sciences Research Complex , University of St. Andrews , EaStCHEM , St. Andrews , Fife , Scotland KY16 9ST , UK . Email: njw3@st-andrews.ac.uk; b State Key Laboratory of Catalysis , Dalian Institute of Chemical Physics , Chinese Academy of Sciences , Dalian , 116023 , China . Email: licz@dicp.ac.cn; c University of Chinese Academy of Sciences , Beijing , 100049 , China

## Abstract

As societal challenges go, the development of efficient biorefineries as a means of reducing our dependence on petroleum refineries is high on the list. One of the core strengths of the petroleum refinery is its ability to produce a huge range of different products using all of the components of the starting material. In contrast, the target of using all the biopolymers present in lignocellulosic biomass is far from realised. Even though our ability to use the carbohydrate-based components has advanced, our plans for lignin lag behind (with the notable exception of vanillin production). One approach to lignin usage is its controlled depolymerisation. This study focuses on an increasingly popular approach to this challenge which involves highly selective lignin oxidation to give a material often referred to as lignin^OX^. But what do we mean by lignin^OX^? In this study we show that it is possible to form many different types of lignin^OX^ depending on the oxidation conditions that are used. We show that variations in the levels of processing of the β–O-4, the β–β and a third linkage occur. Through use of this information, we can form a well-defined lignin^OX^ from six different hardwood lignins. This process is reproducible and can be carried out on a large scale. With a source of well-defined lignin^OX^ in hand, we show that it can be converted to simple aromatic monomers and that any remaining lignin^OX^ is sufficiently soluble for further processing to be carried out.

## Abstract 2

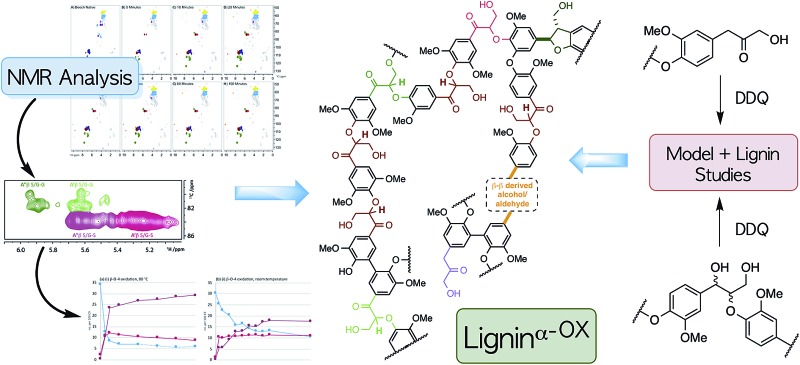
As societal challenges go, the development of efficient biorefineries as a means of reducing our dependence on petroleum refineries is high on the list.

## Introduction

**Fig. 1:**
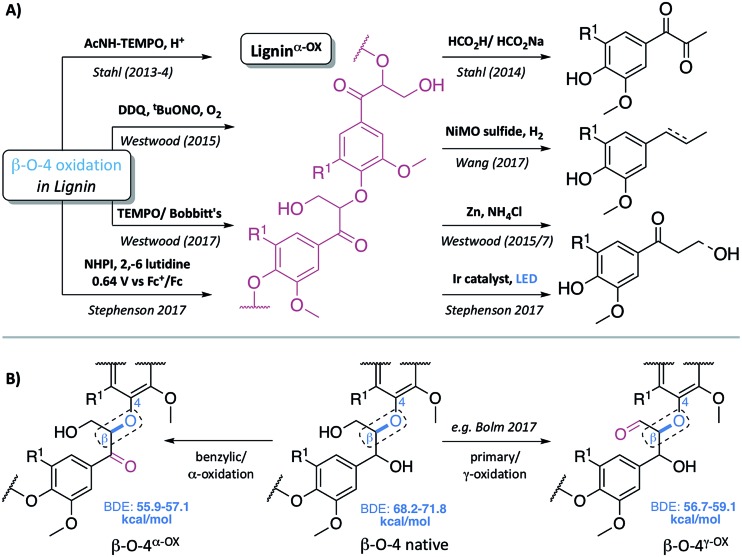
(A) Representative examples of previous approaches to depolymerisation using benzylic oxidation methods to lower C–O-aryl ether bond dissociation enthalpies (BDEs) in the β–O-4 units and so facilitate subsequent cleavages, selected examples are those used on lignin itself.^5^,^6^,^8^,^30^–^32^ (B) Representative structures of the β–O-4, β–O-4^α–OX^ and β–O-4^γ–OX^ (Bolm *et al.*^28^) units within lignin and the corresponding calculated C–O BDEs of native and oxidised states.^27^ R^1^ = H or OMe in hardwoods.

Our society remains addicted to oil and all the products that stem from this natural resource. As the availability of easy to access (and hence cheap) oil reduces, we must identify alternative sources to meet our fuel and, most importantly in the context of the work described here, our chemical feedstock needs. Many view the efficient use of lignocellulosic biomass as one part of a future solution, with impressive progress in the generation of bioethanol from this source already in place.^1^,^2^ To deliver maximum efficiency, however, it is likely that we will have to use all the components of the lignocellulose not just the carbohydrate containing part. Lignin, a complex biopolymer biosynthesised from three phenylpropanoid monomers,^3^,^4^ constitutes up to 30% of the plant cell wall (and hence lignocellulose).^2^ A desire to depolymerise lignin into simple aromatic monomers is driving a considerable amount of research.^5^–^18^ In particular, extensive studies have been carried out on the controlled cleavage of one of lignin's units – the β–O-4 linkage, partly due to its high abundance in “native-like” lignins (the β–O-4 unit represents approximately 60% of the units in hardwoods).^1^,^2^ Although not always the case (*e.g.* TfOH- or metal triflate-mediated depolymerisation of lignin in the presence of ethylene glycol^12^,^19^ and others^20^–^26^) one major depolymerisation approach involves an initial selective oxidation of the β–O-4 linkage in the lignin, followed by cleavage of the C–O aryl bond (Fig. 1A). In this type of approach to lignin depolymerisation, it seems inevitable that the structure of the oxidised lignin will have a significant impact on the success of the C–O-aryl bond cleavage reaction.

Common two step approaches to lignin depolymerisation have focused on selective oxidation of the benzylic alcohol in the β–O-4 linkage, as this chemical transformation leads to a reduction in the bond dissociation enthalpy (BDE) of the C–O-aryl ether bond. Calculated C–O-aryl ether BDEs in β–O-4 linkages decrease from 68.2–71.8 kcal mol^–1^ to 55.9–57.1 kcal mol^–1^ upon benzylic oxidation (*i.e.* in β–O-4^α–OX^ linkages, Fig. 1B).^27^ Oxidation of the primary alcohol in the β–O-4 unit (to give β–O-4^γ–OX^) has also been reported to decrease the C–O-aryl ether BDE to 56.7–59.1 kcal mol^–1^ though to date this has been less well studied.^27^–^29^ It has become clear that the ability to oxidise the β–O-4 linkage in a controlled selective manner represents a very important step in overcoming the recalcitrance of lignin. Successful generation of lignin^α–OX^ (as opposed to related work carried out on model systems^9^,^10^) has been achieved through a variety of methods including: (a) acetamido-TEMPO (AcNH-TEMPO)-mediated catalytic benzylic oxidation^5^,^6^ of an Aspen lignin; (b) Bobbitt's salt and TEMPO-methanesulfonic acid (MsOH)-mediated stoichiometric benzylic oxidation of a Douglas fir lignin and a novel lignosulfonate;^30^ (c) Cp*Ir-catalysed dehydrogenation of benzylic alcohols;^7^ (d) 2,3-dichloro-5,6-dicyano-1,4-benzoquinone (DDQ)-mediated stoichiometric and catalytic benzylic oxidation of a Birch lignin;^8^ (e) DDQ, N-hydroxypthalimide (NHPI), NaNO_2_;^31^ and (f) the recently reported use of NHPI/2,6-lutidine-mediated electrochemical oxidative methods.^32^ Although not studied here, the related generation of lignin^γ–OX^ (Fig. 1B) has been studied. Meier *et al.*^29^ and more recently, Bolm *et al.*^28^ have reported systems, one of them catalytic,^18^ that oxidise selectively the primary alcohol of β–O-4 units in model compounds and lignin itself.

DDQ is known to oxidise efficiently benzylic and allylic alcohols.^8^,^33^–^36^ This reagent is often used stoichiometrically, partly due to the relative ease of the recyclability of DDQ-H_2_ to DDQ (*e.g.* using Mn(OAc)_3_).^34^ Alternatively catalytic DDQ systems in conjunction with a range of co-oxidant systems have been used (for example, NaNO_2_ or ^*t*^BuONO and molecular O_2(g)_;^8^ Mn(OAc)_3_ (ref. 34) and more recently the use of electrochemistry^37^). DDQ has been reported to be an ideal oxidant for selective benzylic oxidation of β–O-4 units in model compounds^33^ and in a birch lignin.^8^ Here we extend the current level of understanding of the material lignin^α–OX^, generating it by reaction with a range of oxidants under different conditions. Our study focuses predominantly on hardwood lignin oxidation using DDQ. Important new insights into the oxidation of the β–β and lignin-bound Hibbert's ketones (LBHK) units, as well as the reactivity of the β–O-4 linkage are presented. As is shown below, the material we refer to as lignin^α–OX^ can vary considerably in structure depending on the details of the oxidation protocol. It seems likely that the structure of lignin^α–OX^ will have a significant knock-on effect on the ability to produce aromatic monomers. We also show that DDQ-mediated oxidation of lignin is general across a range of hardwoods and can be tuned to alter the solubility of the residual lignin after the removal of an initial set of aromatic monomers. This is of importance as one vision of the ideal use of lignin involves a series of carefully controlled depolymerisation steps that ultimately results in the stepwise production of a number of different aromatic compounds in pure form. The overall aim of this work has therefore been to define better what is meant by lignin^α–OX^ in the hope, ultimately, of facilitating the valorisation of this important renewable resource.

## Results and discussion

In our previous studies, lignin^α–OX^ was prepared using catalytic DDQ oxidation with ^t^BuONO and O_2(g)_ as the co-oxidants (Scheme S1 and Fig. S1†).^8^ This procedure was carried out in 2-methoxyethanol and 1,2-dimethoxyethane using a dioxasolv birch lignin.^8^ During this earlier work (and from the work of others,^5^,^6^,^31^,^32^ Fig. S2†) it became clear to us that other linkages, in addition to the β–O-4, in the lignin were also modified, but these additional transformations have not been studied. Here we revisit the generation of lignin^α–OX^, providing a much broader study of this important biomaterial.

In this new work, it was decided to oxidise a dioxasolv *beech* lignin using varying weight equivalents of DDQ (*i.e.* the non-catalytic variant of our previously reported DDQ-oxidation protocol). A reaction temperature of 80 °C, a fixed reaction time of 2 hours and 1,4-dioxane as the solvent were selected as a compromise between the use of moderate reaction temperatures and rapid reaction progress (Fig. S3, S4, Tables S1 and S2†). The stoichiometric reaction was selected to enable more simple control over the reaction conditions. The plan was to use 2D HSQC NMR analysis to track linkage reactivity with increasing amounts of DDQ. In addition to assessing the relative reactivity of the different linkages we were also interested in the reproducibility of the oxidation process and its applicability to a range of different hardwood lignins.

## Beech lignin oxidation study

### Reactivity of the β–O-4 linkages

**Fig. 2:**
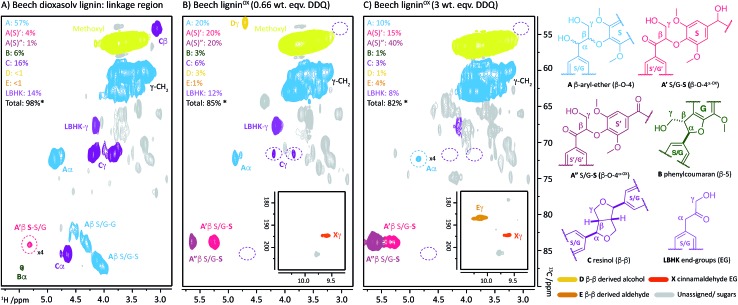
2D HSQC NMR analysis (700 MHz, *d*_6_-DMSO) of: (A) beech dioxasolv lignin linkage region; (B) beech lignin^α–OX^ post reaction with 0.66 wt equiv. of DDQ and; (C) with 3 wt equiv. of DDQ. Percentages were calculated assuming the units present are 100% of the linkage content and are based on semi-quantitative analyses of lignin samples.^38^ For an example of integral analysis see Fig. S5.† For full raw data see Table S3 and Fig. S6.† Assignments of key structures are based on data from this study and the literature.^5^,^8^,^26^,^30^,^39^–^44^ γ-CH_2_ assigned cross-peak refer to all possible alkyl-oxygenated CH_2_-groups present in the lignin chain. Threshold levels in the analysis of the 2D HSQC NMR have been lowered to observe key cross-peaks. The oxidation of G–β–O-4 structures is discussed in more detail in the ESI.† *Total percentage of linkages < 100% due to omission of **A′**/**A′′** S/G–**G** (β–O-4^α–OX^) values, please see Table S3 for further details.†

**Fig. 3:**
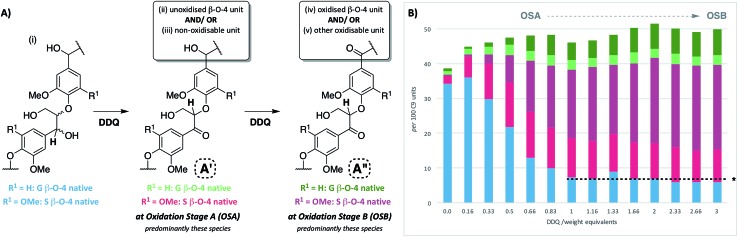
Beech lignin DDQ oxidation study: (A) proposed reactivity of β–O-4 units within lignin including possible oxidized stages within lignin^α–OX^ of the β–O-4 unit. The chemical shifts of the highlighted **H** atoms are reliant on the substituents on the aromatic ring connected *via* the aryl-ether linkage (Fig. S1†); (B) graph displaying β–O-4 and β–O-4^α–OX^ units per 100 C9 units against DDQ weight equivalents; highlighted **H** atoms (and their corresponding cross-peaks within 2D HSQC analysis) were used for semi-quantification of units within lignin.^38^ Data repeated in triplicate, for standard error analysis, see Table S3.† All standard errors associated with values in this figure are <1.5%. *Dashed lines indicate a plateau where no more oxidation was notably occurring. No/very little signal corresponding to remaining β–O-4 native units were observed at this point in 2D HSQC NMR spectra, but background noise and/or new peaks may be overlapping with integral regions rendering these integrals not equal to zero.

Upon treatment of our dioxasolv beech lignin (S : G ratio = 3.4 : 1, Fig. S5†) with increasing weight equivalents of DDQ, many chemical changes were observed based on analysis by 2D HSQC NMR (Fig. 2, S6 and Table S3†). This analytical technique is a semi-quantitative approach and the discussion below is based only on the trends that were reproducibly observed. The starting lignin contained predominantly native β–O-4 units (Fig. 2A, 3A(i) and B, blue section of 0.0 DDQ weight equivalent bar for all possible combinations of S and G native β–O-4 units) with only small amounts of naturally oxidised β–O-4 units being present (Fig. 3A(ii) and B, pink and green sections of 0.0 DDQ wt equiv. bar). Reaction with increasing amounts of DDQ (for example using up to 0.5 weight equivalents) led to a significant decrease in the amount of native β–O-4 units (smaller blue section in bars as DDQ wt equiv. increases) and an increase in the signals corresponding to oxidised β–O-4 units (pink and green sections in bars, Fig. 3A(ii), (iii) and B). These conditions (0.5 wt equiv. DDQ) also led to a number of oxidised β–O-4 units in which the adjacent linkage had been modified (for example dark pink section in 0.5 DDQ wt equiv. bar in Fig. 3B referring to the signal present at 5.65/83.2 ppm in Fig. 2B). In previous work^8^ we have described how the environment of the remaining β-proton in an oxidized β–O-4 unit is affected by oxidation of an adjacent β–O-4 unit (Fig. 3A(iv)). The change in oxidation state at the α-carbon of the adjacent β–O-4 unit is communicated to the phenolic oxygen and hence the next β-hydrogen *via* the aromatic ring (Fig. S1a†). It is also possible that oxidative processing of a different adjacent linkage (not β–O-4) could lead to an analogous (overlapped) cross-peak (Fig. 3A(v)).

The described reactivity trends continued when even more DDQ was used. Comparison of the 2D HSQC NMR spectra obtained for the starting lignin (Fig. 2A) and for the lignin treated with 0.66 wt equiv. of DDQ (Fig. 2B) showed that there was a clear decrease in the size of the cross peaks corresponding to the native β–O-4 units (blue peaks). Linked to this, the cross peaks corresponding to the oxidised β–O-4 units increased (*e.g.* light pink and dark pink sections). It was decided to refer to the lignin prepared in this way as being at Oxidation Stage **A** (OSA – 0.66 wt equiv. DDQ in 1,4-dioxane, 80 °C for 2 hours, pink (light and dark) cross peaks were of approximately equal size).

When even larger amounts of DDQ were used (up to 3 wt equiv.) a static situation was reached in which essentially all the native β–O-4 units had been converted (blue cross peak at 4.87/72.2 ppm is magnified 4-fold in Fig. 2C 38) with the main cross-peak now corresponding to the β-hydrogen of an oxidised β–O-4 unit in the situations shown in Fig. 3A(iv) or A(v).

It should be noted that no matter how much DDQ was used, a cross peak at ^1^H/^13^C 5.32/83.8 ppm (Fig. 2C) was always observed on analysis of the lignin. This presumably results from the fact that some oxidised β–O-4 units in lignin are located adjacent to non-oxidisable units (situation shown in Fig. 3A(iii)), which will be discussed further below in the context of the lignin-bound Hibbert's ketones (LBHK) units. It was decided to refer to the lignin prepared in this way as being at oxidation stage **B** (OSB – 3 wt equiv. DDQ in 1,4-dioxane, 80 °C for 2 hours with essentially no native β–O-4 signal (blue cross peak) and with the dominant cross-peak at 5.65/83.2 ppm in Fig. 2B). Analogous reactivity trends were also observed during the processing of the β–O-4(G) units (see Fig. 3A(i) for structure, see ESI for discussion, Fig. S7 and S8†).

**Fig. 4:**
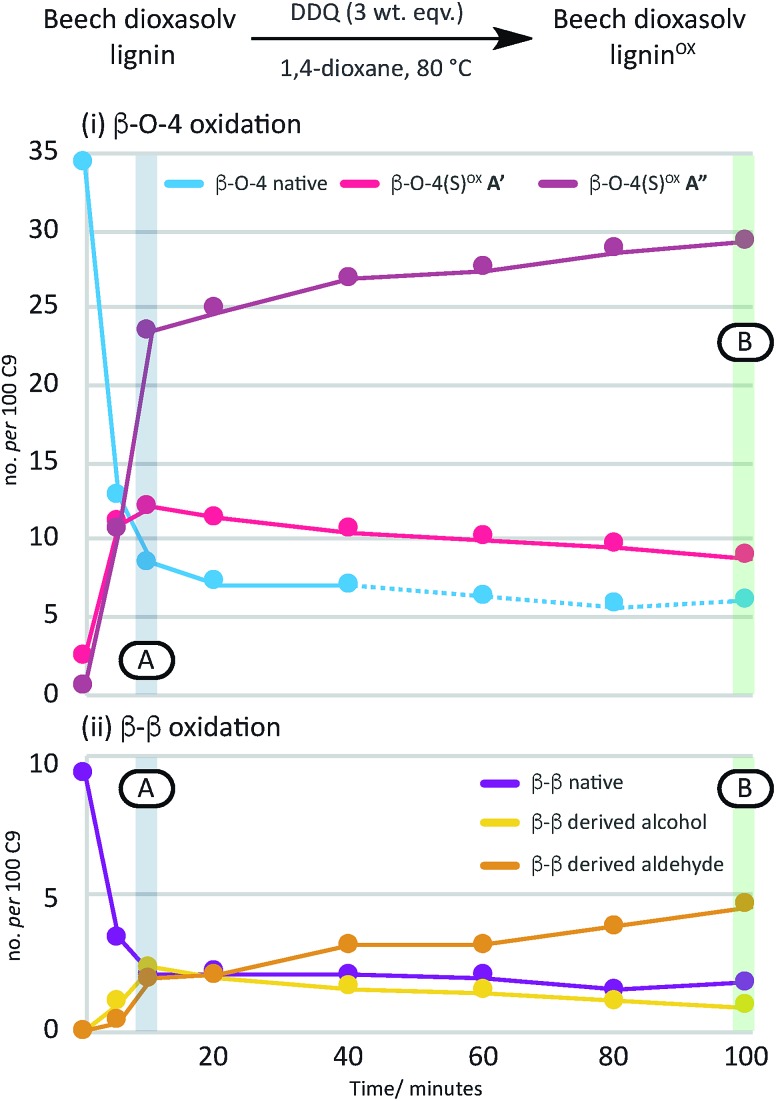
A graphical representation of a time course analysis of the DDQ oxidation of dioxasolv beech lignin. The *X*-axis represents the time (in minutes) and the *Y*-axis represents the no per 100 C9 units for each unit calculated using semi-quantitative 2D HSQC NMR analysis. For full data and graph displaying β–O-4(G)^α–OX^ also, see Table S4 and Fig. S9 and S10† respectively. See also Fig. S11 and S12† for full NMR spectra.

A time course analysis of the oxidation of beech lignin using 3 wt equiv. of DDQ at 80 °C (as judged by 2D HSQC NMR semi-quantitative analysis, Fig. 4i, Table S4, Fig. S9 and S10†) showed a rapid decrease in the native β–O-4 units (blue line) within the first 10 minutes followed by a more gradual decrease in any remaining native β–O-4 units. Consistent with rapid oxidation of the β–O-4 units under these conditions, the signals for the β–O-4^α–OX^ units (*e.g.* light and dark pink lines in Fig. 4) also increased rapidly. After 15 minutes, only minor changes were observed in the β–O-4 and β–O-4^α–OX^ signals (Fig. 2 for structures).^38^

### Reactivity of the β–β units

**Fig. 5:**
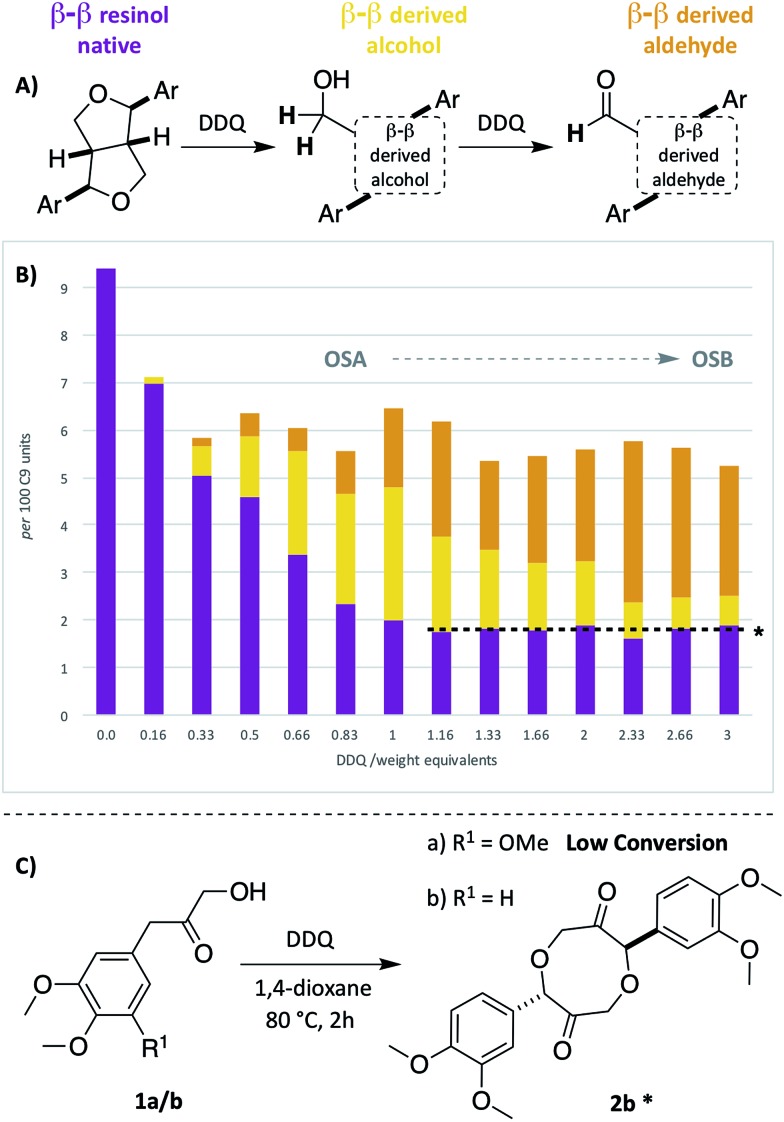
Beech lignin DDQ oxidation study: (A) proposed reactivity of β–β units within lignin. Highlighted **H** atoms (and their corresponding cross-peaks within 2D HSQC analysis) were used for semi-quantification of units within lignin. ^a^(B) Graph displaying β–β, β–β derived alcohol and β–β derived aldehyde units per 100 C9 units against DDQ weight equivalents; data repeated in triplicate, for standard error analysis, see Table S3.† All standard errors associated with values in this figure are <1%. *Dashed lines indicate a plateau where no more oxidation is notably occurring. (C) Reactivity of LBHK model **1** when treated with DDQ (see Scheme S3 and Fig. S17† for crude ^1^H NMR data from reactions). ^§^Proposed structure based on current analytical data (Fig. S17†).

As the second most abundant unit within hardwood lignins, it was important to study the fate of the β–β (resinol) unit under DDQ oxidation conditions. Previous work by Tran *et al.*^45^ has reported the susceptibility of these units in Kraft lignin to DDQ oxidation but to the best of our knowledge no reports on β–β oxidation in mild organosolv lignins exist. In this study, a decrease in the relative intensity of the signals corresponding to this unit occurred even when low weight equivalents of DDQ were used (*c.f.*Fig. 2A and B and see Fig. 5B). For example, at oxidation stage **A**, a more than 50% reduction in the signals corresponding to the β–β unit had occurred. Interestingly a new cross-peak was observed to increase in intensity as the cross-peaks for the native β–β units were decreasing. The chemical shift of this cross-peak (^1^H/^13^C 4.67/53.0 ppm, Fig. 2B) was very similar to that observed in Tran's work with Kraft lignin^45^ and the multiplicity-edited HSQC spectrum confirmed that it corresponded to a CH_2_ group most likely a primary alcohol (R–CH_2_OH). In addition, it was observed that at oxidation stage **B** a significant reduction in the intensity of this cross peak had occurred but that a new aldehyde cross-peak had appeared. The relatively intensity of the new aldehyde peak was consistent with the conversion of the initially formed primary alcohol (R–CH_2_OH) to the corresponding aldehyde (R–CHO). For this alcohol oxidation to occur with DDQ it would be expected that the primary alcohol would be allylic/benzylic or adjacent to a heteroaromatic ring.^38^

Comparison of the rate of conversion of the β–O-4 unit with that of the β–β unit at 80 °C (Fig. 4) showed that the two units are approximately equally susceptible to oxidation by DDQ. The native β–β units (purple line in Fig. 4) decreased by ∼80% within 10 minutes, an equivalent decrease to that of the native β–O-4 units (*ca.* 75%). Interestingly, at 10 minutes, the decrease in the native β–β units was not complemented by an analogous increase in β–β-derived alcohol (yellow line) and β–β-derived aldehyde (orange line) units. At present the structures of the corresponding alcohol and aldehyde are not known and so it is very difficult to speculate on the structures (and hence NMR signals) of any reaction intermediates that may be present. This issue is the focus of on-going studies and we hope to be able to provide an update on this in the near future. At extended reaction times the data support a situation in which the β–β-derived alcohol was predominantly converted to the β–β-derived aldehyde. These data emphasise how important it is that the oxidation conditions are selected carefully. For example, if the reaction had been run with 3.0 wt equiv. of DDQ for 15 minutes at 80 °C (Fig. 4A, blue band) then whilst modification of the β–O-4 units would be essentially complete, a complex situation would have existed with respect to processing the β–β units. By selecting an extended reaction time, for example 2 hours (green band in Fig. 4A), complete modification of both the β–O-4 (to benzylic oxidised units) and β–β units (to the corresponding aldehyde) has occurred.

### Reactivity of lignin-bound Hibbert's ketones

We have recently reported a detailed study on the observation of the lignin-bound Hibbert's ketones (LBHK) unit in organosolv lignins.^26^ The LBHK units are formed during acid-mediated extraction of lignin^46^–^49^ (in this case *via* the dioxasolv process). Under these conditions hydrolysis of the β–O-4 unit occurs in such a way that the LBHK is present as an end group. Whilst therefore the LBHK units are not present at high levels they are positioned in a relatively sterically accessible position in lignin and as such may interfere with planned reactions. In brief, the cross peak at ^1^H/^13^C 4.15/67.5 ppm in Fig. 2A corresponds to the γ-CH_2_ of the LBHK unit. The studies reported here have enabled us to gain a further understanding of the fate of this unit in lignin during DDQ-mediated oxidation. Whilst limited reactivity of this unit was observed at lower weight equivalents of DDQ, it was clear that the LBHK units reacted when higher numbers of equivalents were used (Fig. S6B–N†). Attempts were made to determine the reaction outcome using LBHK models **1** (Fig. 5C). In the presence of a large excess of DDQ, model **1a** (S-LBHK) was unreactive and only minor new baseline peaks were observed in the ^1^H NMR (Fig. S17a†). When G-LBHK model **1b** was treated with an excess of DDQ (3 or 5 equiv.), complete conversion of **1b** was observed. Whilst a relatively complex reaction outcome was observed (Fig. S17b† for crude ^1^H NMR data), the major product of this reaction was assigned as the dimeric structure **2b** (probably formed from trapping of a benzylic carbocation through intermolecular attack of a primary alcohol from another G-LBHK unit, Fig. 5C). Interestingly this suggests that the majority of LBHK units in our beech lignin are G-LBHK units and on reaction with DDQ, any intermediate carbocations that are formed will be trapped either (intermolecularly or intramolecularly) by primary alcohols from other units in the same or other lignin chains.

**Fig. 6:**
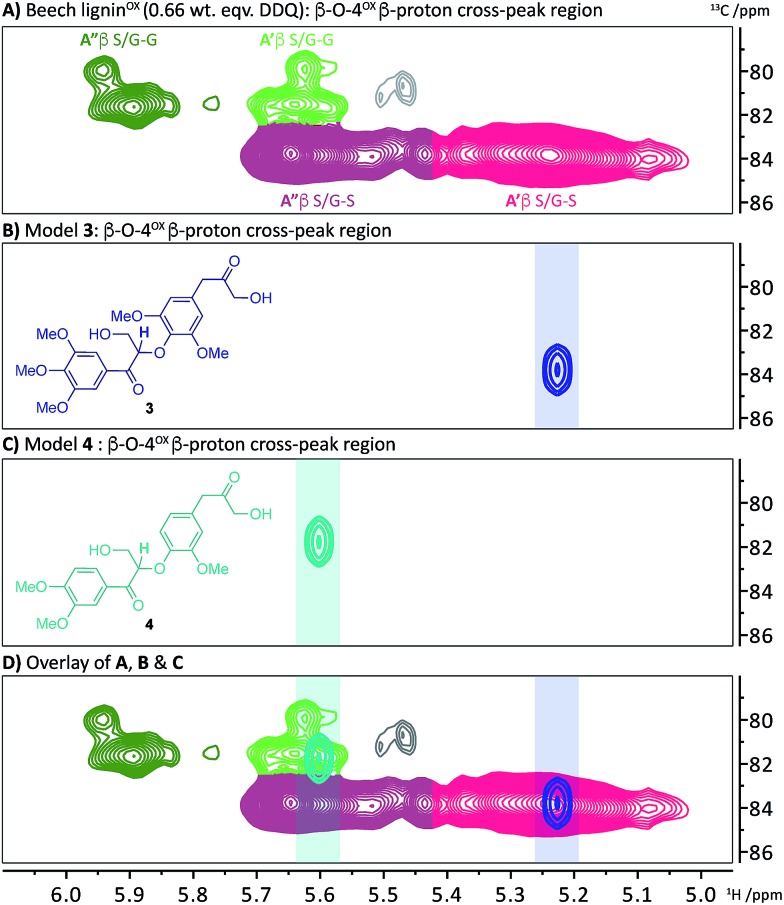
2D HSQC NMR analysis (700 MHz, *d*_6_-DMSO) of the β–O-4^α–OX^ β-proton region of: (A) beech lignin^α–OX^ (0.66 wt equiv.); (B) β–O-4^α–OX^-LBHK model **3**; (C) β–O-4^α–OX^-LBHK model **4** and; (D) an overlay of **A**, **B** and **C**. See Scheme S4† for synthesis of models **3** and **4**.

Based on the insight that not all the LBHK units are converted during the DDQ oxidation (*e.g.* the S-LBHK model **1a** did not react), it was assumed that some LBHK units would be adjacent to oxidised β–O-4 units (*e.g.*Fig. 3A(iii)). If this is the case, it was interesting to consider what the NMR chemical shift of the peak corresponding to the β-proton of the β–O-4^α–OX^ unit would be (given the lack of a corresponding benzylic ketone in the LBHK structure). To this end, model compounds **3** and **4** were synthesised (Scheme S4†) to observe where the peak corresponding to the β-proton of a β–O-4^α–OX^ unit would reside when adjacent to an LBHK end-group (or potential other non-oxidisable group). An overlay of the 2D HSQC NMR spectra (β–O-4^α–OX^ β-proton region) of beech lignin^α–OX^ and model **3** (Fig. 6A and B respectively) showed that the peak corresponding to the β-proton of model **3** (^1^H/^13^C 5.23/83.8 ppm, highlighted in **6B**) overlapped with the β–O-4^α–OX^**A′** region in beech lignin^OX^ (Fig. 6D and 2 for definition of **A′**). An analogous observation was made when comparing the peak corresponding to the β-proton of β–O-4(G)^α–OX^-LBHK model **4** (^1^H/^13^C 5.60/81.8, Fig. 6C) and beech lignin^α–OX^ (Fig. 6A) with good overlap (Fig. 6D) of the signal for model **4** with the β–O-4(G)^α–OX^**A′** region in beech lignin^α–OX^. This comparison explains why the cross-peaks corresponding to β–O-4^α–OX^**A′** units never completely disappear during the oxidation – that is due to non-(poorly)-oxidisable units being present adjacent to β–O-4 units in lignin.

### Comparison of lignin^α–OX^ preparation procedures

So far in this study, an improved understanding of the processing of β–O-4 (**A**), β–β (**C**) and LBHK units within dioxasolv lignin has been achieved. With this information in hand, a comparison of several of the current approaches for generating lignin^α–OX^ was undertaken (Table 1).

**Table 1:** Comparison of lignin^α–OX^ preparation protocols. Outcome of protocol assessed using 2D HSQC NMR and conversion assessed using semi-quantitative NMR analysis and reported as ratios with respect to the native β–O-4 unit **A** (oxidised state **A′** and **A′′**) and the β–β unit **C** (for oxidised states **C** and **D**).^38^

Method^*a*^	Temp. (time)	β–O-4 unit **A** : **A′** : **A′′**	β–β unit **C** : **D** : **E**
DDQ stoich.	r.t (14 h)	1 : 1.4 : 2.2	1 : 0.6 : 0.4
	80 °C (2 h)	1 : 1.8 : 5.5	1 : 0.1 : 2.5
DDQ cat.^8^	80 °C (14 h)	1 : 0.6 : 1.2	1 : 0.1 : 0.2
TEMPO-MsOH stoich.^30^	65 °C (3 h)	1 : 1.3 : 1.6	1 : 0 : 0^*b*^
Bobbitt's salt^*a*^ stoich.^9^	r.t. (24 h)	1 : 1.4 : 2.4	1 : 0.2 : 0.1^*b*^
	65 °C (3 h)	1 : 1.3 : 2.1	1 : 0.1 : 0.1^*b*^

^*a*^It should be noted that although ref. 9 showed the oxidant Bobbitt's salt worked on model systems, this oxidant was not used on lignin in the original study. Unfortunately, several approaches could not be tested by us^2^,^21^,^22^ for reason that included challenges inherent in the use of high pressures of oxygen gas^31^ and the requirement for the use of electrochemistry equipment in a very recent report.^32^ See Fig. S18 and Table S6 for 2D HSQC NMR spectra. **A** and **C** integral values were normalised to a value of 1 for comparison. Values reported to one decimal place.

^*b*^In these reactions the starting β–β unit partially reacted but very little or no **D** or **E** was formed.

Initial comparison of lignin oxidation using stoichiometric DDQ at r.t and 80 °C showed that by heating to 80 °C improved oxidation of the β–O-4 unit occurred (increase in ratio of **A′′** and **A′**). It was also found that units **D** and **E** were formed from the β–β unit **C** (Fig. 5A) but once again the proportion of the aldehyde **E** was highest at 80 °C. When comparing the stoichiometric and catalytic variants of the DDQ oxidation, it was notable that whilst oxidation of the β–β unit **C** did occur, the amounts of **D** and **E** were much lower in the catalytic protocol. In addition, the degree of oxidation of the β–O-4 unit was lower. A similar trend was observed for the TEMPO-MsOH oxidation protocol along with other stoichiometric TEMPO methods (Table 1, entries 3 and 4). Bobbitt's salt oxidised β–O-4 units to a comparable degree to the stoichiometric DDQ oxidation at room temperature, but whilst partial reaction of the β–β unit **C** was observed, formation of **D** and **E** did not occur.

### Application of DDQ oxidation to other hardwoods

**Fig. 7:**
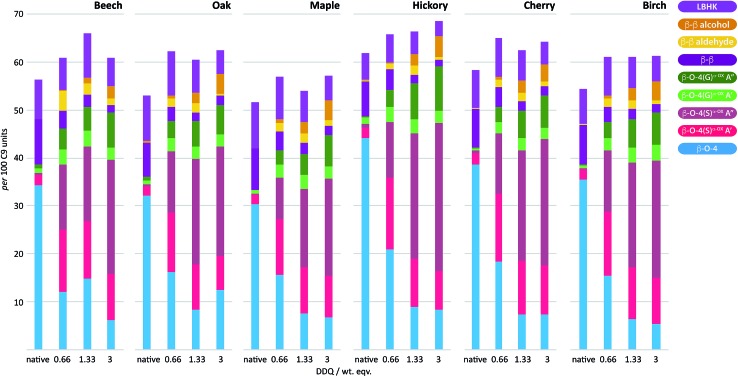
DDQ hardwood lignin oxidation scope study. Analysis of the per 100 C9 units in lignin^α–OX^*vs.* the weight equivalents of DDQ used to generate the lignin^α–OX^ in 6 different lignins; beech, oak, maple, hickory, cherry and birch. Values for per 100 C9 units were generated from 2D HSQC NMR semi-quantitative analysis of lignin samples (*vide supra*). For full data, see Table S7 and Fig. S19–S21.†

To examine the general applicability of the stoichiometric DDQ oxidation procedure, five other hardwood dioxasolv lignins were subjected to oxidation conditions using 0.66 (OSA conditions), 1.33 and 3 (OSB conditions) wt equiv. of DDQ. Reassuringly, using the OSA conditions, the ratio of the oxidised β–O-4 units (light pink *c.f.* dark pink section of bars in Fig. 7) was approximately equal when oak, maple, hickory, cherry or birch lignins were used. Traces of the β–β-derived aldehyde signal were observed under OSA conditions for these new woods (not seen for beech) but overall the native β–β and β–β-derived primary alcohol signals were dominant. Under OSB conditions essentially no native β–O-4 units remained for all the lignins used^38^ and the major unit present in all cases corresponded to oxidized β–O-4 units as shown in Fig. 3A(iii) and (iv). It should be noted that dioxasolv hickory lignin contains a significant amount of this unit under OSB conditions. As was the case for beech lignin, it was also observed across the other lignins that the quantity of LBHK apparently reduced as the amount of DDQ used increased.

**Fig. 8:**
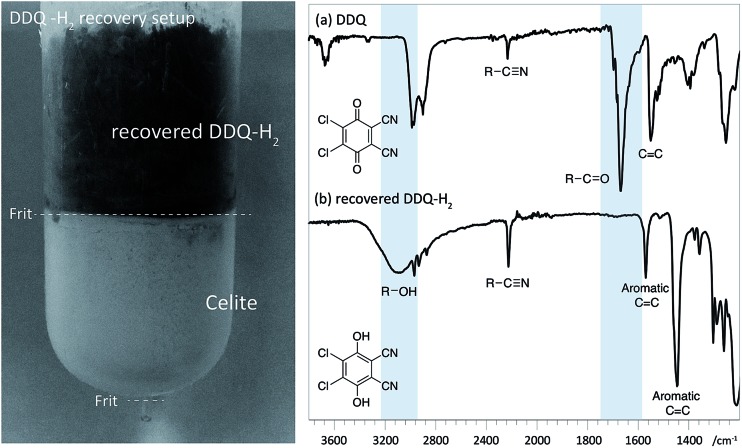
Left: Recovery of DDQ-H_2_ using celite packed fritted column (See Fig. S24 for colour image†). Right: IR spectra (cropped region) of (a) DDQ and; (b) recovered DDQ-H_2_. Characteristic differences in the spectra are highlighted with in blue.

Reassuringly, this section of the study confirms that it is possible to identify reaction conditions that deliver analogous lignin^α–OX^ materials across a range of different hardwood types. With conditions achieved that show different lignins can be oxidised to lignin^α–OX^ reproducibly (Fig. S22†) and controllably to different oxidation stages (*e.g.* OSA or OSB), our interests turned to the scalability of the oxidation procedure. To test this, a 20 gram portion of dioxasolv beech lignin was treated under our optimised conditions to generate an OSB lignin. Gratifyingly, the oxidation proceeded well and the lignin^α–OX^ isolated (97 wt% yield) was of comparable ‘quality’ (Fig. S23 for comparison†) to that isolated from our standard small scale procedure (*ca.* 300 mg). The recoverability of the DDQ-H_2_ was also assessed and analysed (Fig. 8 (ref. 38)). This recovered DDQ-H_2_ can be converted back to DDQ *via* several reported methods.^34^,^35^,^37^

### Applications of alternative states of lignin^α–OX^

As an initial attempt to correlate the influence of the structure of lignin^α–OX^ with the outcome of follow-on protocols carried out on the lignin^α–OX^, it was decided to focus on the impact (*e.g.* solubility) on the residual lignin obtained after zinc-mediated processing of this material. Whilst a wide range of methods of comparing different lignin^α–OX^ samples can be envisioned, this method was selected as in our previous work we had found that the residual lignin we obtained after the production of the aromatic monomers was completely insoluble in any organic solvent (including DMSO). This meant that it was very challenging to characterise the residual lignin (especially by preferred solution phase NMR methods).^1^ In addition, we are interested in a lignin valorisation approach that combines the successful production of aromatic monomers whilst retaining a residual lignin for use in a smart materials application. For this we envisage the need to modify the residual lignin we obtained to deliver the required materials properties. To this end, we proposed that, in addition to producing aromatic monomers, it may be useful to produce a residual lignin that still retained some native β–O-4 units. It was therefore decided to prepare lignin^α–OX^ (*e.g.* in OSA using 0.66 wt equiv. DDQ, Fig. 2B) and to carry out our zinc reductive C–O cleavage method to generate monomers and to deliver a residual lignin that retained native linkages and was soluble in organic solvents.

**Fig. 9:**
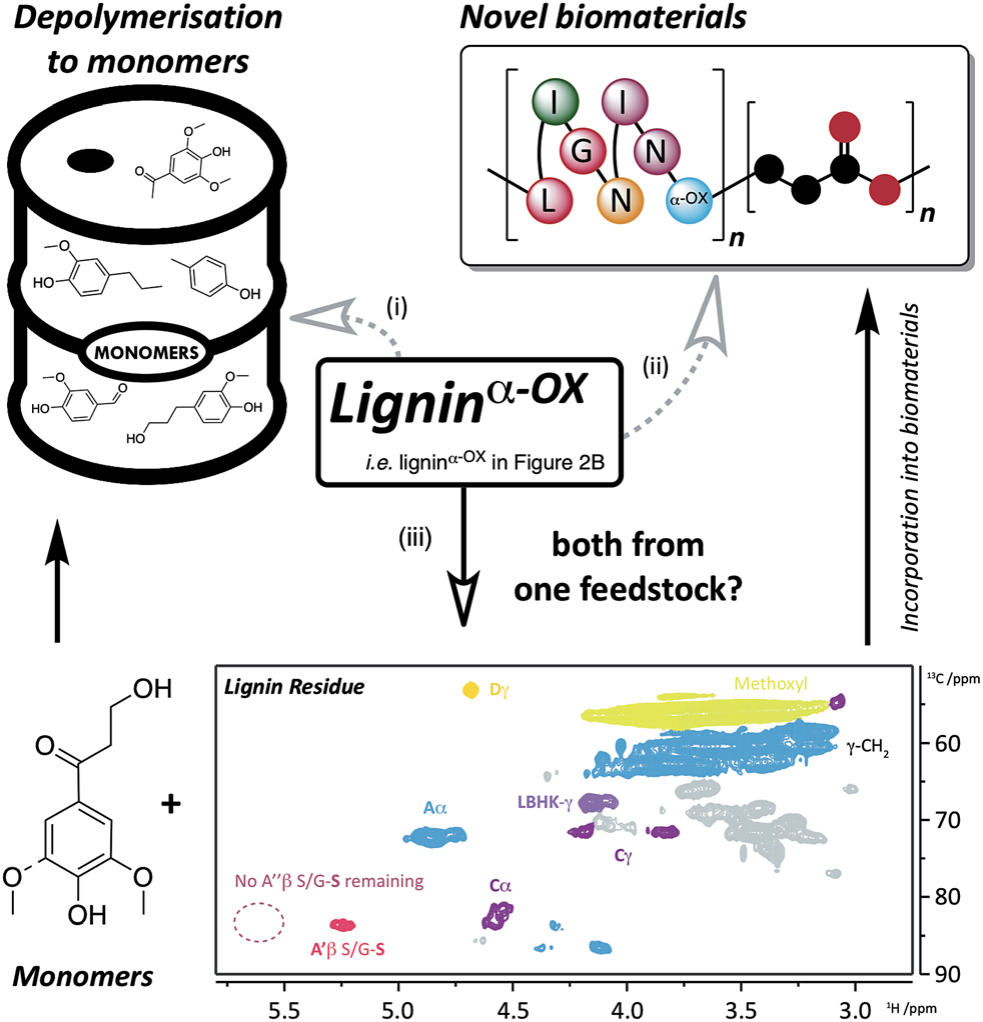
Applications and approaches to the use of lignin^α–OX^. Route (i) depolymerisation of lignin^α–OX^ to monomers (*e.g.*ref. 6, 8, 32, 50 and 51) and; route (ii) incorporation of lignin^α–OX^ into novel biomaterials (*e.g.*ref. 52 and 53). A different approach (iii) using the same feedstock to produce both monomers and biomaterials has been the key target of this work. Route (iii), using a zinc-mediated depolymerisation approach^8^ gives: monomeric keto-alcohol (Fig. S24†) and a soluble lignin residue (inset: 2D HSQC NMR analysis (700 MHz, *d*_6_-DMSO) of residual beech lignin^α–OX^ after zinc reductive cleavage. For coloured contour assignments, see Fig. 2) which through small modifications (*e.g.*ref. 54) can be used to produce novel biomaterials.

A sample of our beech lignin^α–OX^ (in OSA, 0.66 wt equiv. DDQ, *e.g.*Fig. 2B) was subjected to zinc reductive cleavage conditions and was shown to produce the keto-alcohol monomer as previously described (Fig. S25† for ^1^H NMR data).^8^ Gratifyingly, under these conditions the residual lignin was soluble in organic solvents including *d*_6_-DMSO. 2D HSQC NMR analysis of the residual lignin revealed that whilst there was a noticeable reduction in the cross-peak intensity of β–O-4^α–OX^**A′′** (Fig. 9 inset, *c.f.*Fig. 2b) as expected, cross-peaks corresponding to native β–O-4 (light blue), β–β (purple) and β–β derived alcohol (dark yellow) units were all still present. This leaves a wide range of options available for either further lignin depolymerisation strategies, *e.g.* metal triflate induced depolymerisation of the residual native β–O-4 units, or derivitisation of the residual lignin for materials applications (ref. 50–55 for examples).

## Conclusions

If we are to replace the all-important petroleum refinery (at least in part) with a biorefinery, it is essential that we continue to focus on methods of using all the components of lignocellulosic biomass including the lignin. One popular approach for depolymerising lignin to deliver pure aromatic monomers is to oxidise selectively the benzylic position of the major β–O-4 linkage in lignin. This process, by which a material referred to as lignin^α–OX^ is produced, facilitates the subsequent depolymerisation by decreasing the C–O-aryl bond strength. However, there are very limited studies on the structure of the complex material referred to as lignin^α–OX^. Here we address this issue in the hope that the information we provide will enable the development of novel methods to use this important natural resource. For example, we propose that through a detailed understanding of the structure of lignin^α–ox^, it may prove possible to establish a process in which the production of aromatic monomers is combined with the production of designer biopolymers.

Our study uses 2D HSQC analysis to characterise various forms of beech lignin^α–OX^ and provides novel insights into the reactivity of the β–O-4, β–β and lignin-bound Hibbert's ketones units under controlled oxidation conditions. In addition, we can select an oxidation protocol that forms a very similar lignin^α–OX^ from six different hardwood lignins. Importantly, this protocol is reproducible and scalable. Whilst there are many ways forward to maximise the potential of lignin, we believe the information reported here will facilitate the development of protocols that proceed *via* lignin^α–OX^.

## Conflicts of interest

There are no conflicts of interests.

## Supplementary Material

Supplementary information
