# Supplementary material for: Unravelling the enigma of ligninOX: can the oxidation of lignin be controlled?
Source: Chem Sci. 2017 Nov 9;9(3):702–11. doi: 10.1039/c7sc03520a (PMC5869806; doi:10.1039/c7sc03520a)
Supplement: Supplementary file 1 [file SC-009-C7SC03520A-s001.pdf]

Electronic Supplementary Material (ESI) for Chemical Science.
This journal is © The Royal Society of Chemistry 2017

Supporting Information for

Unravelling the Enigma of Lignin^{OX}: Can the Oxidation of Lignin be Controlled?

Haiwei Guo†,[a,b,c] Daniel M. Miles-Barrett†,[a] Andrew R. Neal,[a] Tao Zhang,[b] Changzhi Li,[b]* and Nicholas J. Westwood[a]*

[a] School of Chemistry and Biomedical Sciences Research Complex, University of St. Andrews and EaStCHEM, St. Andrews, Fife, Scotland, KY16 9ST, UK

[b] State Key Laboratory of Catalysis, Dalian Institute of Chemical Physics, Chinese Academy of Sciences, Dalian, 116023, China

[c] University of Chinese Academy of Sciences, Beijing, 100049, China

†These authors have contributed equally to this work

*Corresponding author information

Changzhi Li: *licz@dicp.ac.cn*

Nicholas Westwood: *njw3@st-andrews.ac.uk*

Table of Contents

**Pages S1-S2**	Notes referred to from Manuscript
**Pages S3-S4**	General Information & Procedures
**Pages S5-S8**	Lignin$^{\alpha\text{-OX}}$ NMR Spectra definitions and assignments
**Pages S9-S12**	Solvent & Temperature Screen for DDQ oxidation of Lignin
**Pages S13-S19**	Lignin oxidation weight equivalent study
**Pages S20-S30**	S-G/G-G-β-O-4 Oxidation Discussion
**Pages S31-S33**	G- vs. S- β-O-4 Competitive Oxidation of Model Compounds
**Pages S34-S41**	Study of LBHK model compounds with DDQ
**Pages S42-S53**	Synthesis of β-O-4-LBHK advanced model compounds
**Pages S54-S55**	Comparison of Lignin$^{\alpha\text{-OX}}$ Preparation Procedures (Manuscript Table 1)
**Pages S56-S61**	Scope Study on other Hardwood Lignins (Manuscript Figure 7)
**Page S62-S63**	Reproducibility, scalability and depolymerisation of Lignin $^{\alpha\text{-OX}}$
**Page S64**	References
**Pages S65-S90**	^{1}H, ^{13}C NMR and other spectra of novel compounds

Notes referred to from Manuscript

[a] Whilst the overall % of oxidised units do not equal the values for un-oxidised units, differences in the NMR relaxation properties of the lignin and lignin^{OX} materials may account for this but remain hard to measure.

[b] When 1 or more wt. eqvs. of DDQ was used, a plateau was reached and the signal apparently corresponding to the presence of residual native β-O-4 units could be attributed to background noise/other new cross-peaks in the 2D HSQC NMR spectra (see Figure 2C that shows that only trace cross peaks corresponding to native β-O-4 units were present when 3 wt. equivalents of DDQ was used).

[c] Unexpectedly, the apparent rate of increase of the species corresponding to the dark pink line and the light pink line in Figure 4A were approximately equal under these reaction conditions. It seems likely that the generation of signals corresponding to β-O-4$^{\alpha\text{-OX}}$ **A(S)''** (dark pink) would require population of the β-O-4$^{\alpha\text{-OX}}$ **A(S)'** (light pink) state. Perhaps at 80 °C the rates of generation and consumption of these β-O-4$^{\alpha\text{-OX}}$ **A(S)'** (light pink) state are equally rapid. When this experiment was repeated with the only change being in the reaction temperature (run at room temperature (for 14 hours) rather than 80 °C, **Figures S9-S10** and **Table S5**), our expected outcome was observed. An initial increase in β-O-4$^{\alpha\text{-OX}}$ **A(S)'** (light pink) state (**See Figure S10b**) was observed after 30 minutes, whilst a lower level of β-O-4$^{\alpha\text{-OX}}$ **A(S)''** (dark pink) was noted. This agrees with the necessity for population of the β-O-4$^{\alpha\text{-OX}}$ **A(S)'** (light pink) state prior to generation of increasing amounts of β-O-4$^{\alpha\text{-OX}}$ **A(S)''** (dark pink) state.

[d] Whilst S- β-O-4 oxidation occurs fast, analysis of G- β-O-4 oxidation in beech lignin appears to reach its maxima (*i.e.* as much G- β-O-4$^{\alpha\text{-OX}}$ **A"** that can be generated, has been), in even less time (*c.f.* Figure 4 and **Figures S9-10**). This is likely due to the increased rate of oxidation of G-benzylic alcohols over S-benzylic alcohols. To explore this further, time-course analyses were undertaken using S and G β-O-4 model compounds (See SI, **Scheme S2**, **Figures S13-S16**). These studies supported this view. The outcome of these studies on oxidation of the β-O-4 unit showed that at any given time point within the initial ten minutes of the reaction at 80 °C, G- β-O-4 model oxidation reached higher conversions than the G- β-O-4 model system to the corresponding benzylic ketone models.

[e] one possibility is that the β-β unit has been converted to the previously proposed pyran-4-one structure (see references [1–3]). Additional transformations are possible and are the subject of on-going work in our groups.

[f] Whilst only 40% of the spent DDQ was recovered as DDQ-H_2 (which can be converted back to DDQ using MnO_2 (an excess in Et_2O, stirred at r.t. for 2 hours and collected by filtration and concentration of filtrate to give DDQ in a *ca.* 54% yield based on two repeats), the residual 60% (~54% recovered as a crude mixture, 43% isolated as DDQ through a $CHCl_3$ recrystallization of the crude mixture) is recoverable from the diethyl ether precipitation step required to precipitate lignin$^{\alpha\text{-OX}}$ through concentration of the filtrate.

General Information & Procedures

Relevant ^{1}H NMR spectroscopic data and melting point analysis (if required) is provided for literature compounds. Full characterisation data consisting of ^{1}H NMR, ^{13}C NMR, 2D HSQC NMR (where applicable), IR, M.P (if possible) and HRMS are provided for all non-literature compounds.

All materials were used as received unless otherwise stated. Diisopropylamine was distilled prior to use and stored over KOH. *n*-BuLi was titrated using diphenylacetic acid. Dry solvents were acquired from an MBRAUN (MB-SPS-800) dry solvent purification machine. ^{1}H NMR and ^{13}C NMR was performed on a Bruker Ascend 400 MHz, Bruker Avance 500 MHz, Bruker Avance III 500 with nitrogen cooled broadband probe or Bruker AVANCE III HD 700 5mm ^{1}H,^{13}C,^{15}N triple resonance inverse nitrogen cooled probe (TCI Prodigy) spectrometer with solvent peak used as internal standard. Multiplicities reported as following: s = singlet, d = doublet, t = triplet, q = quartet and m = multiplet and J values are reported in Hz. Column chromatography was conducted using Davisil® silica (40-63 µm, 230-400 mesh) on a Biotage Isolera One Spektra system with ACI. Thin layer chromatography was performed using pre-coated glass plates (Silica gel 60A from Fluorochem) and visualised under UV light (254 nm) or through staining with $KMnO_4$, 4-dinitrophenylhydrazine (aldehydes) and $FeCl_3$ (phenols). IR spectra were obtained on a Shimadzu IRAffinity-1 Fourier Transform IR spectrophotometer as thin films. IR analysis was carried out using IResolution v1.50 with only characteristic peaks reported. Mass spectrometry data was acquired through the University of St Andrews School of Chemistry mass spectrometry service or EPSRC Swansea Mass Spectrometry Service. Reactions at -78 °C were achieved using acetone: dry ice. Any reactions requiring anhydrous conditions were run using oven-dried (140 °C) or flame dried glassware under N_2 atmosphere.

Lignin Dioxasolv Extraction: as previously described.[4,5]

DDQ Oxidation of Lignin

To a stirring solution of lignin (1 wt. eq.) in 1,4-dioxane (100 mg/ 2.33 mL) is added DDQ (varying wt. eq.). The solution is heated to 80 °C for 2 hours, cooled, filtered through a pad of celite and washed with 1,4-dioxane (1 volume). The filtrate is added dropwise to Et_2O (10

volumes) and the resulting precipitate is filtered and washed with excess Et_2O. Lignin$^{\alpha\text{-OX}}$ is dried to constant weight in a vacuum oven at 40 °C for 24 hours prior to analysis.

β-**O-4 model compounds S1-S2**: Synthesised as previously described.[4,5]

2D HSQC NMR Acquisition

Oven-dried lignin samples (70 mg) are dissolved in 0.6 mL of d_6-DMSO in a 1.5 mL eppendorf tube and subjected to sonication for 10 minutes at 30 °C. Samples are centrifuged at 6000 RPM for 5 minutes. Supernatant is filtered through a 0.45 µM syringe filter into an over dried NMR tube. 2D HSQC NMR spectra were acquired on a Bruker Avance III 500 with nitrogen cooled broadband probe or Bruker AVANCE III HD 700 5mm ^{1}H,^{13}C,^{15}N triple resonance inverse nitrogen cooled probe (TCI Prodigy) spectrometer. The central DMSO solvent peak was used as internal reference (δC 39.5, δH 2.49 ppm). The 1H, 13C-HSQC experiment was acquired using standard Bruker pulse sequence 'hsqcetgpsp.2' (phase-sensitive gradient-edited-2D HSQC using adiabatic pulses for inversion and refocusing). Composite pulse sequence 'garp4' was used for broadband decoupling during acquisition. 2048 data points was acquired over 12 ppm spectral width (acquisition time 170 ms) in F2 dimension using 24 scans with 1 s interscan delay and the d4 delay was set to 1.8 ms (1/4J, J = 140 Hz). A spectral width of 86 ppm (47-133 ppm) and 128 increments were acquired in F1 dimension (acquisition time 5.9 ms). The spectrum was processed using squared cosinebell in both dimensions and LPfc linear prediction (32 coefficients) in F1. Volume integration of cross peaks in the HSQC spectra was carried out using MestReNova 11.0 for Mac software and figures were prepared using Adobe Illustrator CS6 for Mac for spectral annotation.

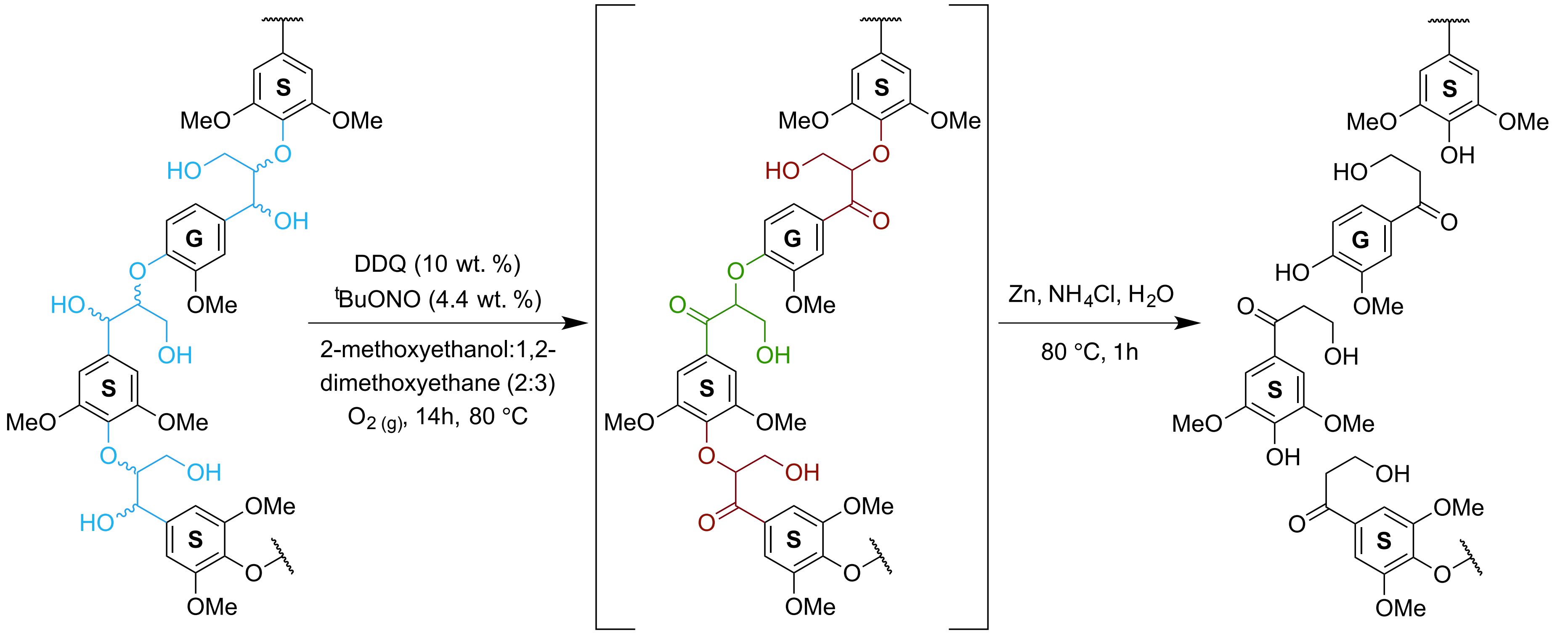

Scheme S1: Westwood group methodology for one-pot lignin depolymerisation.[5] Lignin is initially oxidised selectively using a catalytic DDQ system followed by a stoichiometric zinc mediated reductive cleavage of the C-O-aryl ether bond. The reductive cleavage of the C-O-aryl bond leads to the generation of a lignin-bound keto-alcohol, a monomeric keto-alcohol and a free phenol end-group on a lignin chain.

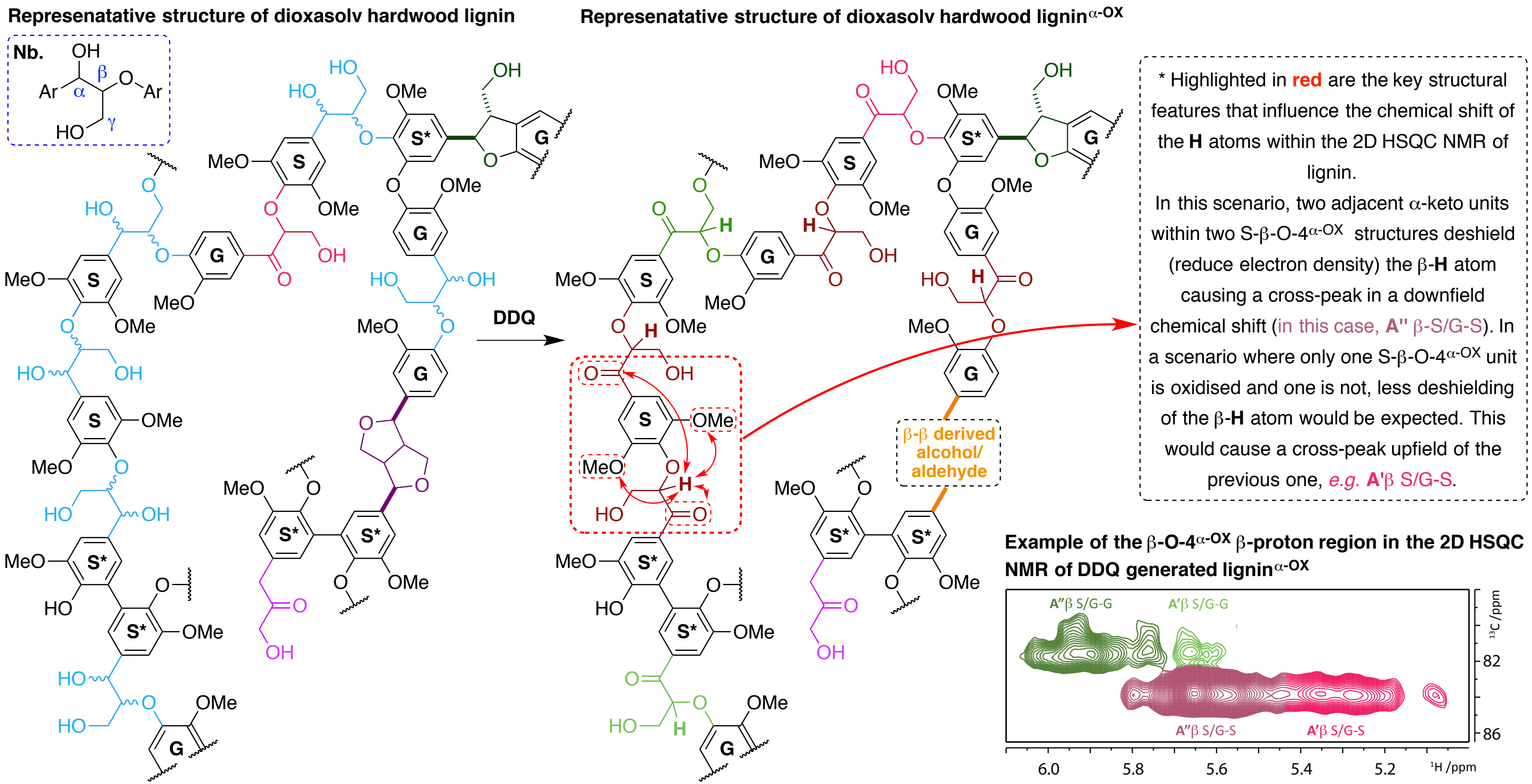

Figure S1a: Representative structure and reactivity of a hardwood dioxasolv lignin that has been selectively oxidised at the benzylic position of the β-O-4 unit. An example of a 2D HSQC NMR spectrum (selected region in insert) is shown to illustrate the key cross-peaks that are used to assess the structures within lignin and how different β-O-4$^{\alpha\text{-OX}}$ units are assigned. Cross peaks are assigned based on comparison of the chemical shifts with those of model compounds.[4,5]

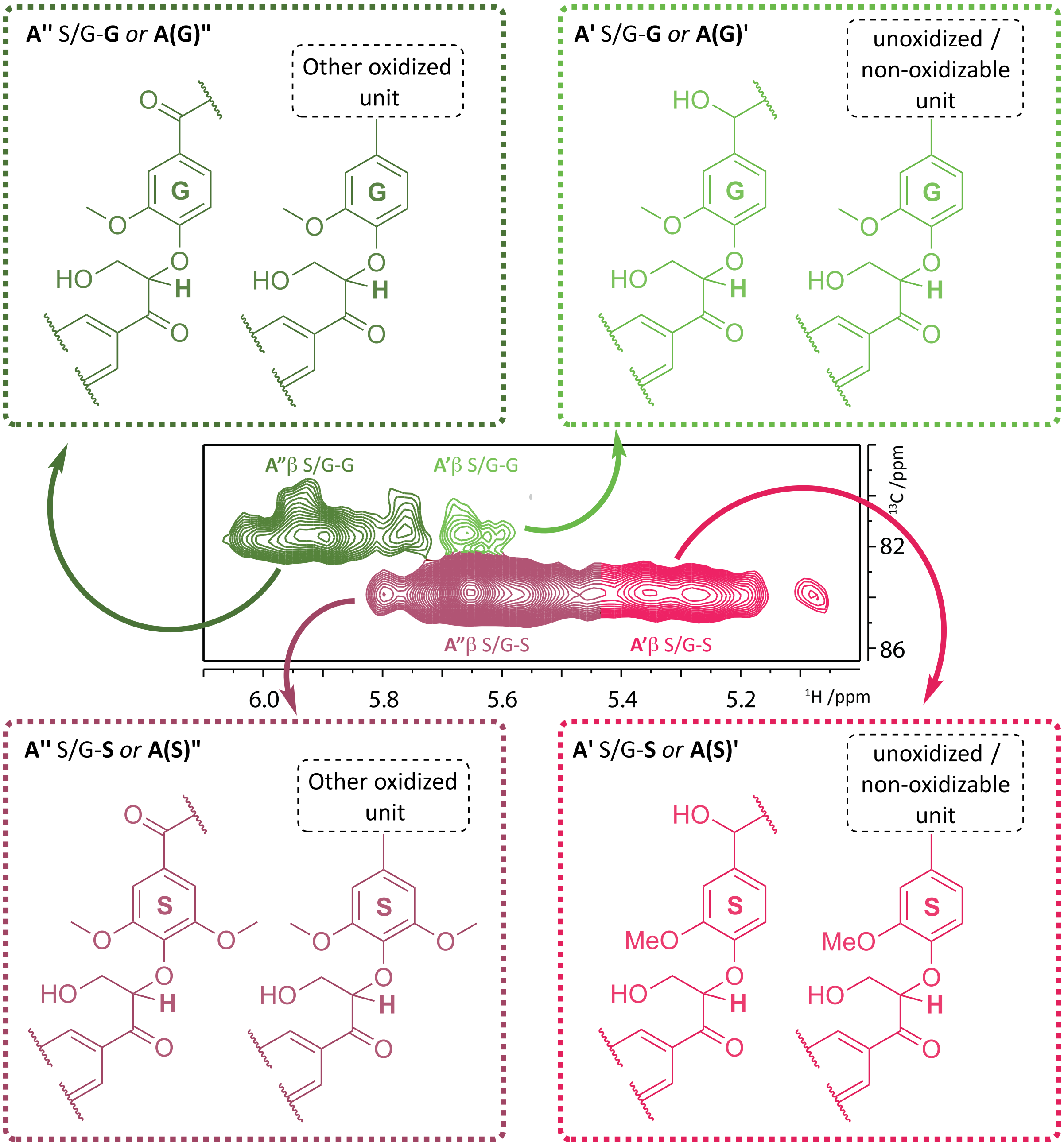

Figure S1b: A diagram explaining the different scenarios of β-O-4$^{\alpha\text{-OX}}$ units present within a lignin polymer which could give rise to an appearance of a cross-peak in the 2D HSQC NMR of lignin$^{\alpha\text{-OX}}$

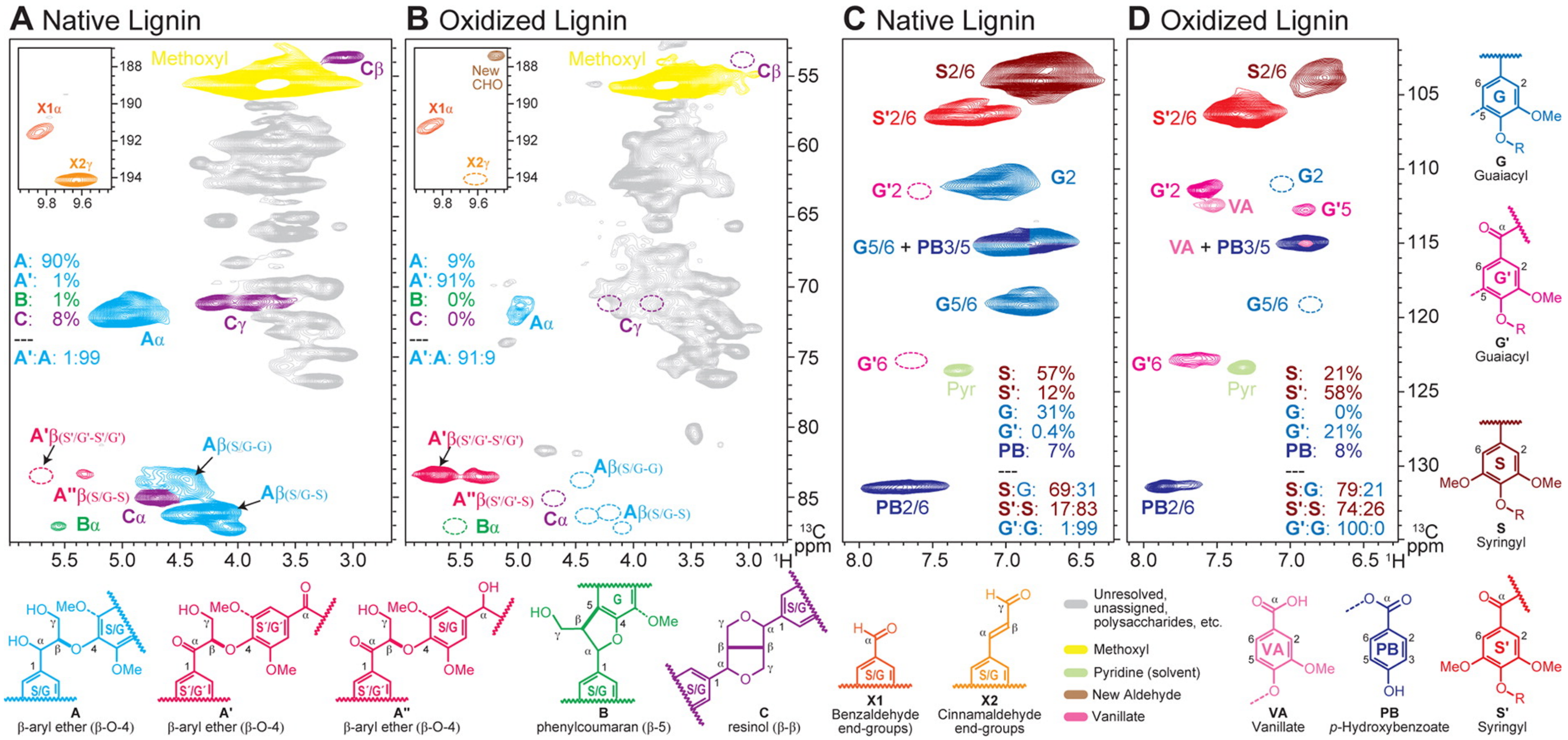

Figure S2: An example of a 2D HSQC NMR of lignin$^{\alpha\text{-OX}}$ generated using a catalytic AcNH-TEMPO system by Stahl *et al.*[6] Key changes, circled above, include near complete loss of β-O-4 units (**A**) (as expected), β-5 (**B**) (unexplained loss) and β-β (**C**) (unexplained loss). Reprinted (adapted) with permission from Stahl *et al. J. Am. Chem. Soc.*, **2013**, *135* (17), pp 6415–6418. Copyright © **2013** American Chemical Society.

Solvent & Temperature Screen for DDQ oxidation of Lignin

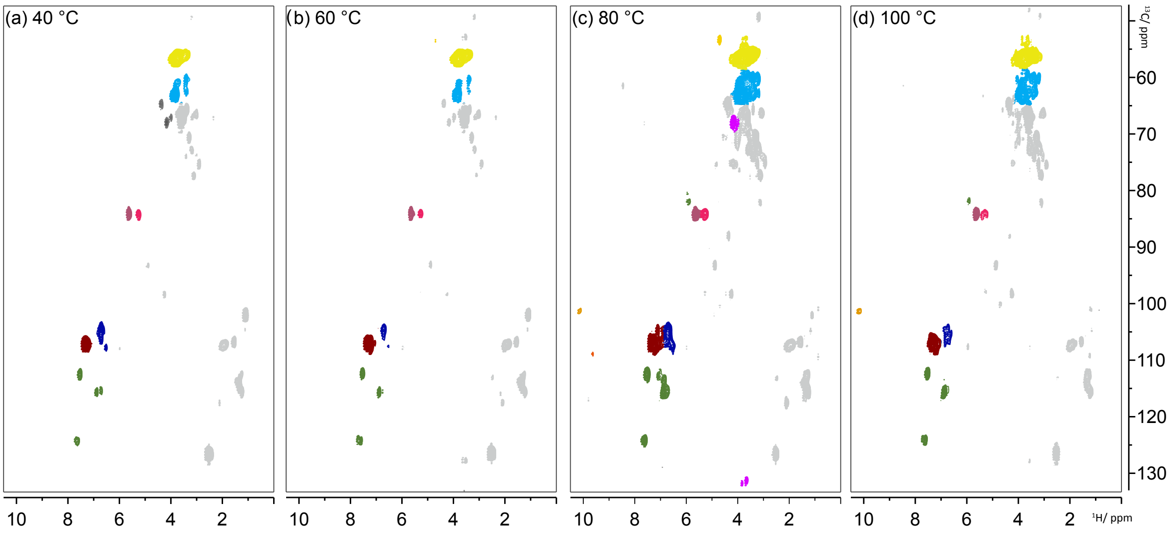

Figure S3: 2D HSQC NMR (d_6-DMSO) of beech lignin$^{\alpha\text{-OX}}$ from the DDQ oxidation of beech lignin at (a) 40 °C; (b) 60 °C; (c) 80 °C and; (d) 100 °C. Reaction conducted with 1.33 wt. eqv. of DDQ in 1,4-dioxane for 2 hours. Spectra acquired using a reduced ^{13}C sweep with of 47-133 ppm.[1] For colour coding see Figure S6 or Figure 2 in manuscript.

Table S1: Results from temperature screen of DDQ lignin oxidation

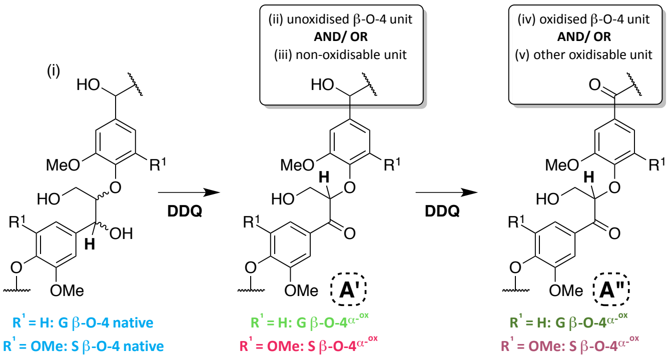

Temperature	β-O-4^{OX} in situation (ii) or (iii) above: β-O-4^{OX} in situation (iv) or (v) above	Note
40 °C	1: 1.2	-
60 °C	1: 1.6	-
**80 °C**	**1: 1.8**	Comparable results, therefore 80 °C selected
100 °C	1: 1.9	

From the temperature screen, the highest amount of the β-O-4 linkage in situations (iv) or (v) was achieved at 80 °C and 100 °C, both yielding comparable results. 80°C was therefore carried through to all the following reactions.

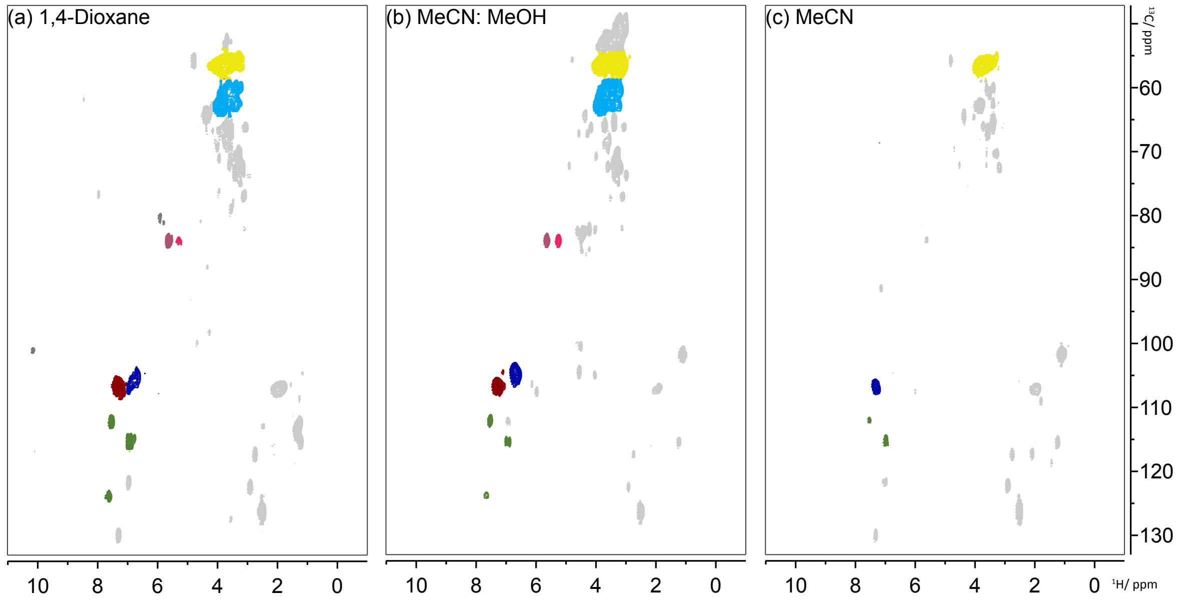

Figure S4: 2D HSQC NMR (d_6-DMSO) of beech lignin$^{\alpha\text{-OX}}$ from the DDQ oxidation of beech lignin when using (a) 1,4-dioxane; (b) MeCN: MeOH (9:1) and; (c) MeCN; as the reaction solvent. Reactions were conducted with 1.33 wt. eqv. DDQ for 2 hours at 80 °C. Spectra acquired using a reduced ^{13}C sweep with of 47-133 ppm.[1] For colour coding see Figure S6 or Figure 2 in manuscript.

Table S2: Results from solvent screen of DDQ lignin oxidation

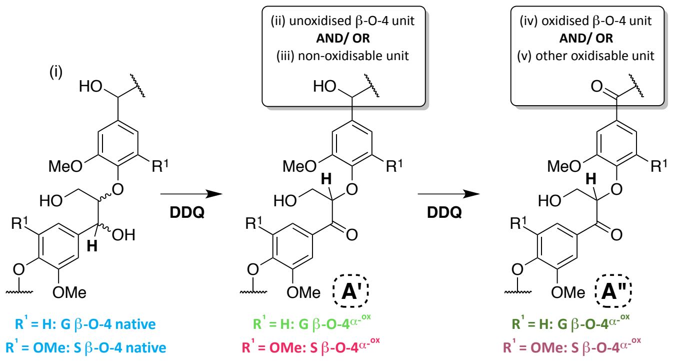

Solvent	β-O-4^{OX} in situation (ii) or (iii) above: β-O-4^{OX} in situation (iv) or (v) above	Solubility	Note
1,2-dimethoxyethane	n/a	Low	-
MeCN	1: 1.89	Medium	-
MeCN: MeOH	1: 1.23	High	Solvent incorporation
**1,4-dioxane**	**1: 2.03**	High	Highest **OSB** achieved

From the solvent screen (and more solvents in which the beech dioxasolv lignin was insoluble, not shown), 1,4-dioxane was decided on as the optimum solvent. The highest ratio of β-O-4^{OX} in situation (iv) or (v) to β-O-4^{OX} in situation (ii) or (iii) was obtained in this solvent which

should allow for more monomers to be produced by a C-O cleavage-mediated depolymerisation strategy (*e.g.* Scheme S1). The choice of non-alcoholic solvents also prevented any chance of incorporation of solvent molecules into the lignin structure.

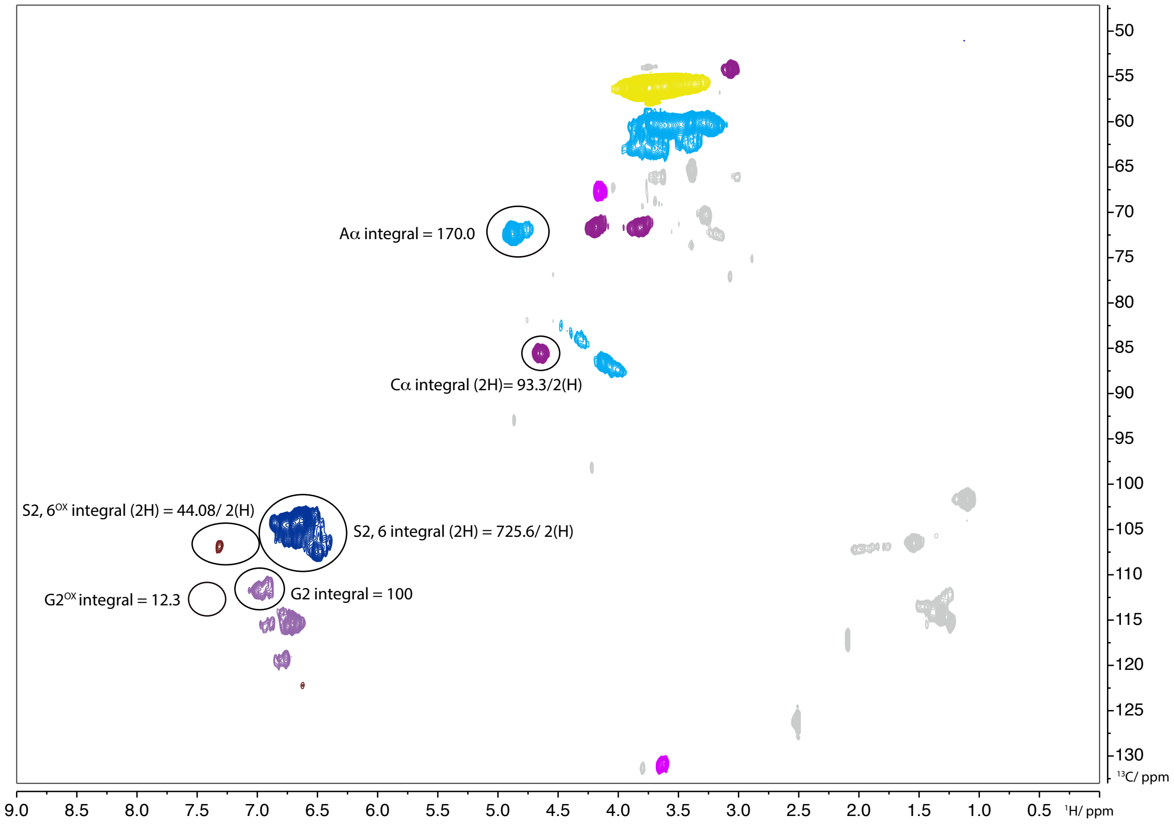

Figure S5: 2D HSQC NMR analysis (700 MHz, d_6-DMSO) with integral analysis of beech dioxasolv lignin. For assignment of structures based on colour coding, see Figure S6 or Manuscript Figure 2. Spectra acquired using a reduced ^{13}C sweep with of 47-133 ppm.[1]

$$\textbf{Equation 1: } \boldsymbol{No\ per\ 100\ C9\ units} = \frac{\boldsymbol{unit\ integral}}{\boldsymbol{G2{+}G2OX\ integral{+}(\frac{S2,6{+}S2,6OX\ integral}{2})}}\ \boldsymbol{x\ 100}$$

$$\boldsymbol{e.g.}\ \textit{for}\ \beta\text{-O-4 (A)}\boldsymbol{.}\ \boldsymbol{No\ per\ 100\ C9\ units} = \frac{\boldsymbol{170}}{\boldsymbol{100{+}12.3\ integral{+}(\frac{725.6{+}44.1}{2})}}\ \boldsymbol{x\ 100}$$

No *per* 100 β-O-4 (A) units = **34.2 units (34)**

Throughout the course of this study, pre-defined regions were used for all integration on MestReNova 11.0 for Mac.

MestReNova Integral Regions

From(f2)	To(f2)	From(f1)	To(f1)	Unit
5.131600	4.632200	69.317300	74.558200	**Aα**
5.598900	5.258300	86.078600	89.147100	**Bα**
4.836300	4.495400	83.143500	87.729200	**Cα**
4.281800	4.001700	65.700200	69.240800	**LBHKγ**
7.534400	7.003900	104.787600	108.773100	**S2,6^{OX}**
7.207500	6.791200	109.525500	113.813600	**G2**
7.044200	6.256400	101.177700	109.218300	**S2,6**
5.780600	5.423200	82.076700	85.370600	**A(S)”**
5.437300	5.110300	82.108300	85.366500	**A(S)’**
6.089900	5.769300	78.915700	82.922900	**A(G)”**
5.754900	5.459700	79.308900	82.052100	**A(G)’**
4.789500	4.579400	51.463700	54.309100	**D**
10.307300	9.966600	99.567800	102.184800	**E** (when spectrum folded)*
7.718500	7.317300	109.752800	114.106700	**G2^{OX}**
10.254300	9.970400	184.673000	189.368000	**E** (when spectrum is unfolded)§

*Spectrum acquired using a reduced ^{13}C sweep width of 47-133 ppm

§ Spectrum acquired using ^{13}C sweep width of 0-170 ppm, o2p spectral centre moved to 125 ppm from 85 ppm.

Table S3: Integral data from 2D HSQC NMR analysis of beech lignin DDQ oxidations (varied weight equivalents). In the first grouping of values ($S_{2,6}$, $S_{2,6}^{OX}$, G_2 (integral set arbitrarily to 100 units), G_2^{OX}), a value is obtained (in triplicate) for the total aromatic integral obtained from 2D HSQC NMR analysis using the integral regions described below Figure S5. The second grouping (β-O-4 native, β-O-4^{OX} A(S)', β-O-4^{OX} A(S)'', β-β resinol native, β-β derived alcohol, β-β derived aldehyde, LBHK, β-O-4^{OX} A(G)' and β-O-4^{OX} A(G)'') is reported as a *per* 100 C9 value obtained from using Equation 1 as is the norm. Raw integral values for the latter part of the table were omitted for clarity (can be back-calculated from Equation 1).

DDQ Eqv.	0.0	0.16	0.33	0.5	0.66	0.83	1	1.16	1.33	1.66	2	2.33	2.66	3
							**$S_{2,6}$**							
REPEAT 1	362.8	301.1	319.6	348.2	310.9	233.6	281.0	253.0	263.5	228.9	258.0	232.5	226.1	246.6
REPEAT 2		279.9	327.5	315.1	295.7	222.3	257.8	214.9	247.0	229.5	240.7	225.5	224.1	213.9
REPEAT 3		306.1	372.3	341.3	266.2	250.9	224.5	236.2	227.7	222.9	210.6	231.7	252.1	241.6
**STDEV**		13.9	28.4	17.5	22.7	14.4	28.4	19.1	17.9	3.7	24.0	3.8	15.6	17.6
**ST.ERROR**		8.0	16.4	10.1	13.1	8.3	16.4	11.0	10.3	2.1	13.9	2.2	9.0	10.2
							**$S_{2,6}^{OX}$**							
REPEAT 1	22.0	56.7	103.0	213.0	294.3	323.5	339.5	341.9	299.8	463.8	529.9	517.7	497.6	534.9
REPEAT 2		52.2	107.3	165.0	289.8	303.1	400.1	388.3	463.4	420.2	537.1	489.4	543.1	484.2
REPEAT 3		59.5	115.1	183.9	310.4	356.8	380.6	444.2	429.7	444.5	492.6	490.8	630.1	565.2
**STDEV**		3.7	6.1	24.2	10.8	27.1	30.9	51.2	86.4	21.8	23.9	15.9	67.3	40.9
**ST.ERROR**		2.1	3.5	14.0	6.3	15.7	17.9	29.6	49.9	12.6	13.8	9.2	38.9	23.6
							**G_2**							
REPEAT 1	100.0	100.0	100.0	100.0	100.0	100.0	100.0	100.0	100.0	100.0	100.0	100.0	100.0	100.0
REPEAT 2		100.0	100.0	100.0	100.0	100.0	100.0	100.0	100.0	100.0	100.0	100.0	100.0	100.0
REPEAT 3		100.0	100.0	100.0	100.0	100.0	100.0	100.0	100.0	100.0	100.0	100.0	100.0	100.0
							**G_2^{OX}**							
REPEAT 1	12.3	23.2	55.9	98.6	106.5	116.0	132.3	145.4	111.9	159.6	181.9	167.8	171.1	173.9
REPEAT 2		16.7	48.3	70.0	106.2	124.4	147.2	139.3	164.9	139.3	186.0	151.2	194.9	155.3

REPEAT 3		23.6	54.4	76.7	122.0	135.5	130.6	152.3	152.1	150.9	163.3	164.9	202.9	171.3
**STDEV**		3.9	4.0	15.0	9.0	9.8	9.2	6.5	27.7	10.2	12.1	8.9	16.5	10.1
**ST.ERROR**		2.2	2.3	8.6	5.2	5.7	5.3	3.8	16.0	5.9	7.0	5.1	9.5	5.8
**Total Aromatics**														
REPEAT 1	497.1	481.0	578.5	759.8	811.7	773.0	852.8	840.3	775.2	952.3	1069.8	1017.9	994.8	1055.4
REPEAT 2		448.8	583.0	650.1	791.6	749.8	905.1	842.5	975.3	889.0	1063.8	966.1	1062.1	953.3
REPEAT 3		489.2	641.7	701.8	798.5	843.2	835.7	932.7	909.5	918.2	966.5	987.3	1185.1	1078.1
**β-O-4 native**														
REPEAT 1	**34.2**	**35.6**	**29.8**	**19.7**	**12.0**	**10.0**	**6.0**	**7.8**	**14.8**	**5.6**	**6.3**	**4.0**	**6.5**	**6.2**
REPEAT 2		**36.8**	**29.6**	**23.4**	**14.5**	**10.9**	**6.8**	**7.3**	**5.8**	**9.4**	**6.6**	**7.7**	**4.8**	**5.1**
REPEAT 3		**35.9**	**30.0**	**22.2**	**12.6**	**9.3**	**9.2**	**5.6**	**6.4**	**5.6**	**8.1**	**5.8**	**6.3**	**6.6**
AVG	**34.2**	36.1	29.8	21.8	13.0	10.1	7.3	6.9	9.0	6.9	7.0	5.8	5.9	6.0
**STDEV**		0.6	0.2	1.9	1.3	0.8	1.7	1.1	5.0	2.2	1.0	1.8	1.0	0.8
**ST.ERROR**		0.4	0.1	1.1	0.8	0.5	1.0	0.7	2.9	1.3	0.6	1.0	0.6	0.5
**β-O-4^{OX} A(S)’**														
REPEAT 1	**2.3**	**5.3**	**9.4**	**12.7**	**13.1**	**11.7**	**11.5**	**12.4**	**11.9**	**9.9**	**9.5**	**10.3**	**9.4**	**9.6**
REPEAT 2		**6.2**	**11.0**	**12.7**	**13.0**	**11.5**	**10.9**	**10.3**	**9.6**	**10.6**	**10.2**	**10.0**	**9.8**	**9.0**
REPEAT 3		**6.1**	**10.0**	**13.0**	**13.3**	**11.7**	**11.6**	**10.0**	**10.7**	**10.8**	**10.9**	**9.8**	**8.0**	**9.4**
AVG	**2.3**	5.9	10.1	12.8	13.1	11.6	11.3	10.9	10.7	10.5	10.2	10.0	9.0	9.3
**STDEV**		0.5	0.8	0.2	0.1	0.1	0.4	1.3	1.2	0.5	0.7	0.3	0.9	0.3
**ST.ERROR**		0.3	0.5	0.1	0.1	0.1	0.2	0.7	0.7	0.3	0.4	0.1	0.5	0.2
**β-O-4^{OX} A(S)”**														
REPEAT 1	**0.4**	**1.1**	**2.3**	**9.3**	**13.5**	**17.5**	**19.4**	**20.6**	**15.6**	**22.4**	**24.3**	**24.1**	**23.3**	**23.8**
REPEAT 2		**0.1**	**3.0**	**7.1**	**14.7**	**17.0**	**19.1**	**21.1**	**22.1**	**22.4**	**24.1**	**24.0**	**25.2**	**24.6**
REPEAT 3		**0.9**	**2.8**	**7.5**	**15.7**	**19.1**	**20.2**	**22.1**	**22.1**	**23.3**	**25.2**	**24.0**	**25.0**	**24.7**
AVG	**0.4**	0.7	2.7	8.0	14.6	17.9	19.6	21.3	19.9	22.7	24.5	24.0	24.5	24.4
**STDEV**		0.5	0.3	1.1	1.1	1.1	0.5	0.8	3.8	0.5	0.6	0.0	1.0	0.5

**ST.ERROR**		0.3	0.2	0.7	0.6	0.6	0.3	0.4	2.2	0.3	0.3	0.0	0.6	0.3
**β-β resinol native**														
REPEAT 1	**9.4**	**6.9**	**4.2**	**4.0**	**3.7**	**2.3**	**2.3**	**1.8**	**2.5**	**1.3**	**1.8**	**1.7**	**1.7**	**1.6**
REPEAT 2		**6.8**	**5.0**	**5.3**	**3.1**	**2.3**	**1.3**	**1.8**	**1.4**	**2.2**	**2.0**	**1.4**	**2.1**	**2.2**
REPEAT 3		**7.1**	**5.9**	**4.5**	**3.3**	**2.4**	**2.3**	**1.7**	**1.5**	**1.9**	**1.8**	**1.8**	**1.6**	**1.9**
AVG	9.4	7.0	5.0	4.6	3.4	2.3	2.0	1.8	1.8	1.8	1.9	1.6	1.8	1.9
**STDEV**		0.2	0.9	0.7	0.3	0.1	0.6	0.0	0.6	0.5	0.1	0.2	0.3	0.3
**ST.ERROR**		0.1	0.5	0.4	0.2	0.0	0.3	0.0	0.4	0.3	0.1	0.1	0.2	0.2
**β-β derived alcohol**														
REPEAT 1	**-0.2**	**0.6**	**1.1**	**3.0**	**4.1**	**3.1**	**4.8**	**4.0**	**2.4**	**2.8**	**1.9**	**1.8**	**1.6**	**1.2**
REPEAT 2		**0.1**	**1.1**	**2.3**	**4.5**	**5.6**	**6.2**	**3.5**	**3.4**	**2.4**	**2.7**	**1.0**	**1.5**	**1.3**
REPEAT 3		**0.2**	**1.5**	**2.3**	**4.4**	**5.2**	**6.0**	**4.6**	**4.0**	**3.2**	**3.6**	**1.8**	**0.9**	**1.2**
AVG	-0.2	0.1	0.6	1.3	2.2	2.3	2.8	2.0	1.6	1.4	1.4	0.8	0.7	0.6
**STDEV**		0.2	0.3	0.4	0.2	1.3	0.8	0.5	0.8	0.4	0.8	0.5	0.4	0.1
**ST.ERROR**		0.1	0.2	0.2	0.1	0.8	0.4	0.3	0.5	0.2	0.5	0.3	0.2	0.0
**β-β derived aldehyde**														
REPEAT 1	**-0.1**	**0.0**	**0.3**	**0.8**	**0.3**	**1.3**	**2.0**	**3.3**	**1.1**	**2.2**	**2.5**	**4.0**	**3.4**	**2.7**
REPEAT 2		**-0.3**	**0.1**	**0.1**	**0.6**	**0.7**	**1.8**	**2.1**	**2.1**	**2.3**	**2.1**	**2.9**	**3.0**	**3.0**
REPEAT 3		**0.2**	**0.1**	**0.7**	**0.5**	**0.8**	**1.1**	**1.8**	**2.5**	**2.3**	**2.5**	**3.2**	**3.0**	**2.4**
AVG	-0.1	0.0	0.2	0.5	0.5	0.9	1.6	2.4	1.9	2.3	2.3	3.4	3.1	2.7
**STDEV**		0.3	0.1	0.4	0.2	0.4	0.5	0.8	0.7	0.1	0.2	0.5	0.2	0.3
**ST.ERROR**		0.2	0.1	0.2	0.1	0.2	0.3	0.5	0.4	0.0	0.1	0.3	0.1	0.2
**LBHK**														
REPEAT 1	**8.4**	**7.2**	**8.7**	**9.6**	**6.7**	**7.9**	**6.4**	**6.0**	**9.2**	**6.1**	**6.3**	**5.0**	**8.0**	**5.9**
REPEAT 2		**7.9**	**8.3**	**8.9**	**8.3**	**8.1**	**5.5**	**5.5**	**6.2**	**6.2**	**5.4**	**5.3**	**5.1**	**4.1**
REPEAT 3		**8.3**	**8.6**	**8.1**	**9.0**	**7.7**	**7.2**	**5.6**	**6.1**	**6.0**	**5.7**	**4.9**	**5.0**	**4.8**

AVG	8.4	7.8	8.5	8.9	8.0	7.9	6.4	5.7	7.2	6.1	5.8	5.1	6.0	4.9
**STDEV**		0.5	0.2	0.7	1.1	0.2	0.8	0.3	1.7	0.1	0.5	0.2	1.7	0.9
**ST.ERROR**		0.3	0.1	0.4	0.7	0.1	0.5	0.2	1.0	0.1	0.3	0.1	1.0	0.5
							**β-O-4^{OX} A(G)’**							
REPEAT 1	**0.9**	**1.1**	**2.3**	**2.5**	**3.2**	**3.2**	**3.3**	**2.3**	**3.4**	**4.0**	**2.6**	**3.5**	**2.4**	**2.5**
REPEAT 2		**2.3**	**2.3**	**3.4**	**3.0**	**2.5**	**2.8**	**3.0**	**2.8**	**2.7**	**2.7**	**2.2**	**3.1**	**3.1**
REPEAT 3		**1.4**	**2.3**	**2.5**	**2.8**	**2.9**	**2.3**	**2.6**	**2.3**	**3.2**	**2.6**	**3.3**	**2.9**	**2.9**
AVG	0.9	1.6	2.3	2.8	3.0	2.9	2.8	2.6	2.9	3.3	2.6	3.0	2.8	2.8
**STDEV**		0.6	0.0	0.5	0.2	0.3	0.5	0.4	0.6	0.7	0.1	0.7	0.4	0.3
**ST.ERROR**		0.4	0.0	0.3	0.1	0.2	0.3	0.2	0.3	0.4	0.0	0.4	0.2	0.2
							**β-O-4^{OX} A(G)”**							
REPEAT 1	**0.8**	**0.8**	**1.9**	**2.2**	**4.3**	**6.4**	**4.1**	**4.5**	**5.0**	**7.0**	**7.0**	**7.7**	**6.5**	**7.4**
REPEAT 2		**0.8**	**1.3**	**1.8**	**4.3**	**5.5**	**5.2**	**4.7**	**6.0**	**6.7**	**6.5**	**6.9**	**7.2**	**7.2**
REPEAT 3		**0.2**	**0.5**	**2.1**	**4.4**	**5.8**	**6.2**	**6.0**	**6.3**	**7.0**	**7.7**	**6.7**	**7.3**	**7.4**
AVG	0.8	0.6	1.2	2.0	4.3	5.9	5.2	5.0	5.8	6.9	7.1	7.1	7.0	7.4
**STDEV**		0.4	0.7	0.2	0.0	0.5	1.0	0.9	0.7	0.2	0.6	0.5	0.4	0.1
**ST.ERROR**		0.2	0.4	0.1	0.0	0.3	0.6	0.5	0.4	0.1	0.3	0.3	0.2	0.1

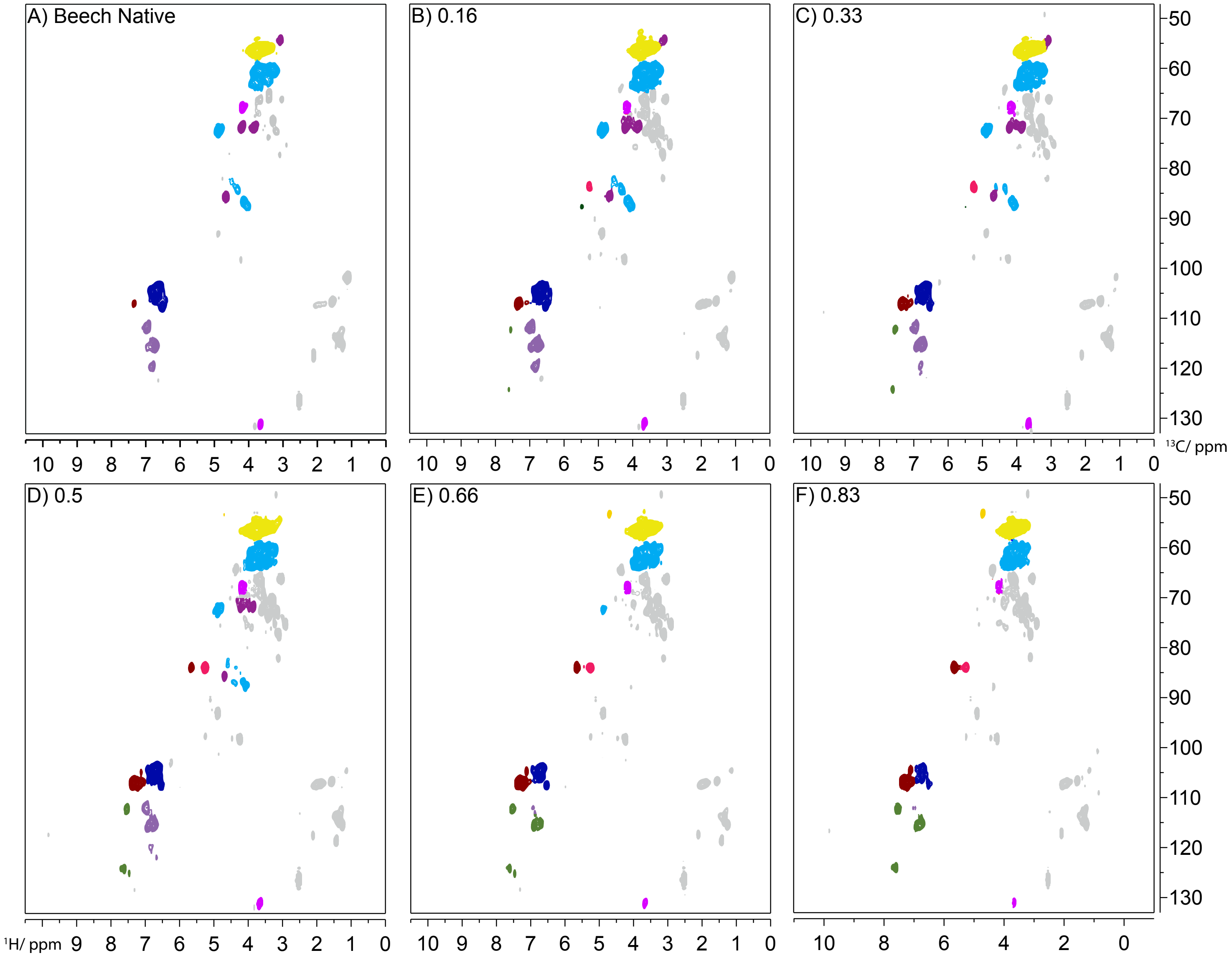

A) Beech Native
B) 0.16
C) 0.33
D) 0.5
E) 0.66
F) 0.83
¹H/ ppm
¹³C/ ppm

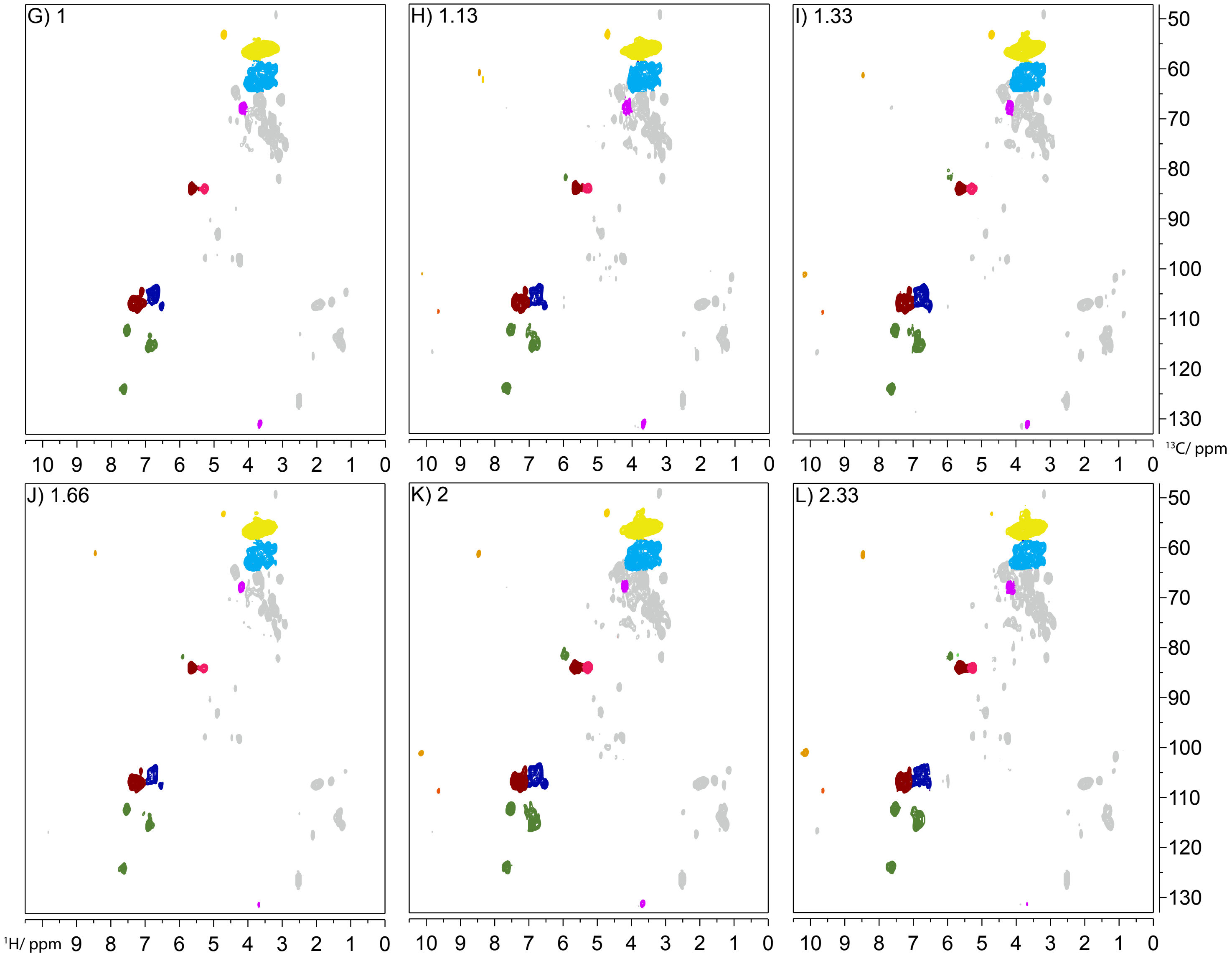

G) 1
H) 1.13
I) 1.33
J) 1.66
K) 2
L) 2.33
^{13}C/ ppm
^{1}H/ ppm

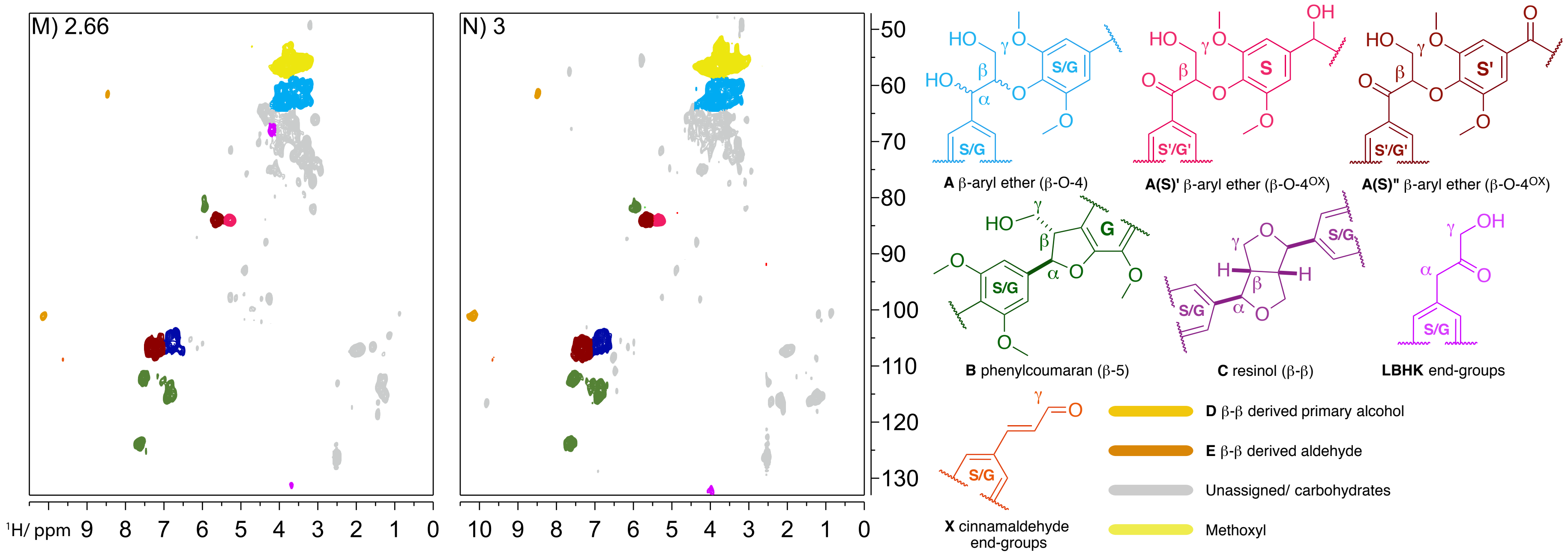

Figure S6: 2D HSQC NMR analysis (700 MHz, d_6-DMSO) of beech dioxasolv lignin (A) and beech lignin$^{\alpha\text{-OX}}$ (B-N) from DDQ weight equivalents study. An example spectrum from each wt. eqv. of DDQ (B-N) is shown. Spectra acquired using a reduced ^{13}C sweep with of 47-133 ppm.[1] Dark green units in the aromatic region refer to oxidised G aromatic units (*e.g.* G'/ G"); dark blue refer to non-oxidised S aromatic units (*e.g.* S) and dark red refer to oxidised S aromatic units (*e.g.* S'/S").

S-G/G-G-β-O-4 Oxidation Discussion

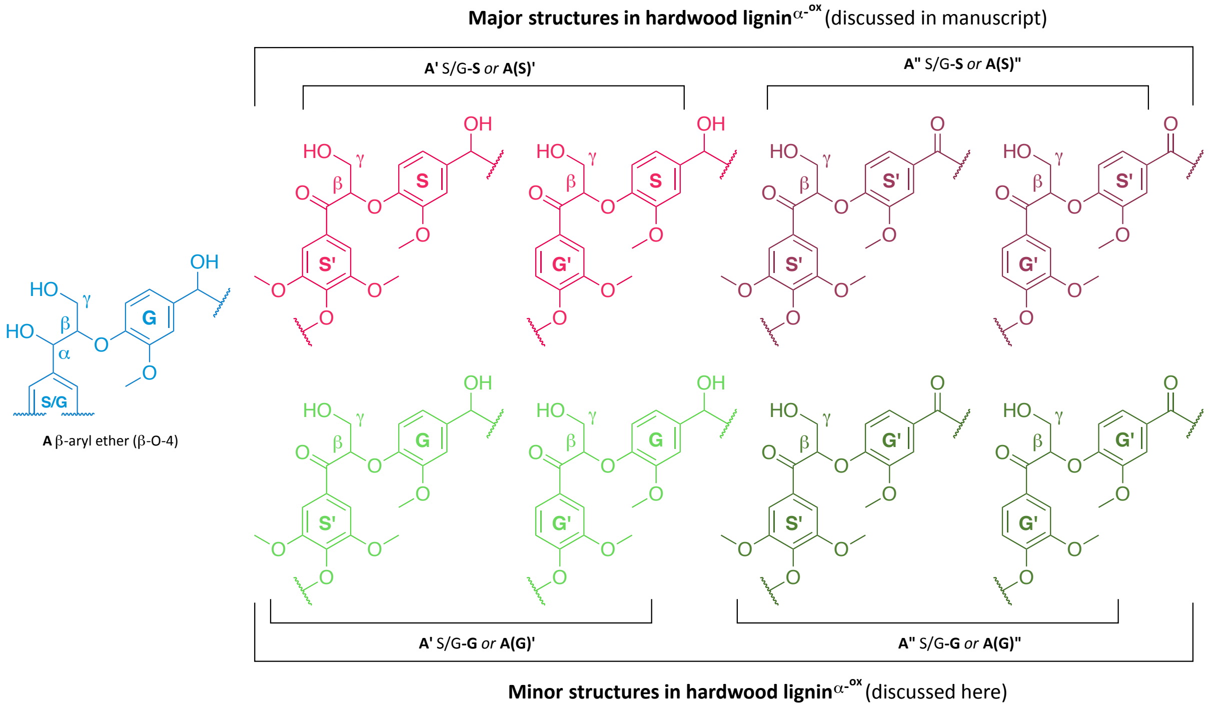

Figure S7a: Structures S/G variations of β-O-4 units. We are discussing S/G-**G** (green) in this section.

In general, in the 2D HSQC spectra we obtained, the cross peaks corresponding to the S-**S**$^{\alpha\text{-OX}}$ or G-**S**$^{\alpha\text{-OX}}$ containing β-O-4$^{\alpha\text{-OX}}$ linkages were dominant compared with the G-**G**$^{\alpha\text{-OX}}$ or S-**G**$^{\alpha\text{-OX}}$ signals (Figure S7a-b). This results from the fact that the S:G ratio was 3.3:1 for the batch of dioxasolv beech lignin (from semi-quantitative 2D HSQC NMR analysis) that was isolated for this study though this will vary depending on the extraction method. This assumes that S:G ratio in the β-O-4 units mirrors the overall S:G ratio (which may well not be the case). Despite the complexities, Figure S7b clearly demonstrates how much smaller the signals corresponding to the G-**G**/S-**G** β-O-4$^{\alpha\text{-OX}}$ linkages are compared to those corresponding to the S-**S**/G-**S** β-O-4$^{\alpha\text{-OX}}$ linkages. Given the low percentage of all unit content that the G-**G**/S-**G** β-O-4 units correspond to, it is unsurprising that the signals are not always observed by 2D HSQC NMR (*c.f.* Figure S6B-N).

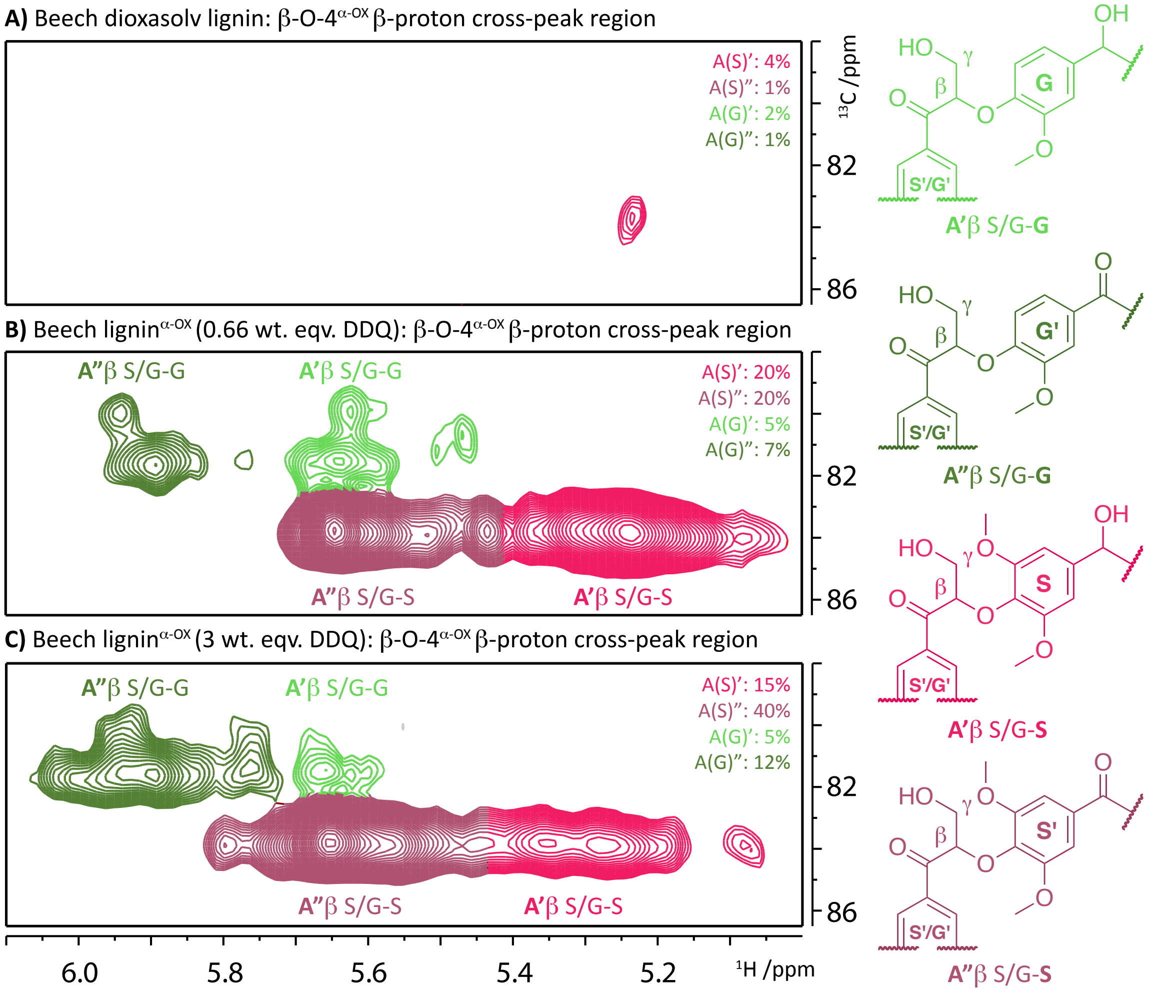

Figure S7b: 2D HSQC NMR (700 MHz, d_6-DMSO) analysis of the β-O-4$^{\alpha\text{-OX}}$ β-proton region of **A**) the starting beech dioxasolv lignin; **B**) Beech lignin$^{\alpha\text{-OX}}$ (0.66 wt. eqv. DDQ) and; **C**) Beech lignin$^{\alpha\text{-OX}}$ (3 wt. eqv. DDQ).

Examining S-G and G-G β-O-4 oxidation (see Manuscript for discussion of G-S or S-S β-O-4 unit oxidation)

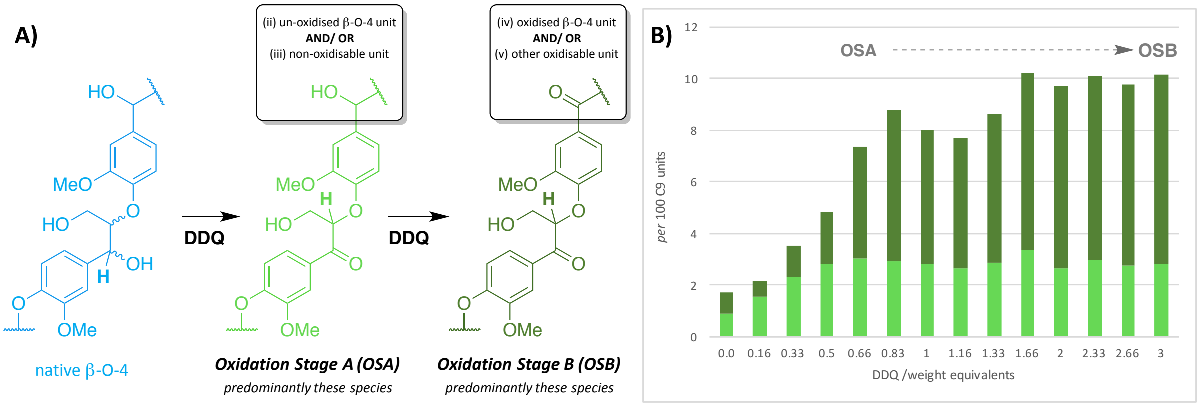

Figure S8: Beech Lignin DDQ oxidation study: **A)** Proposed reactivity of G- β-O-4 units within lignin including possible oxidized stages within lignin$^{\alpha\text{-OX}}$ of the G- β-O-4 unit. The chemical shifts of the highlighted H atoms are reliant on the substituents on the aromatic ring connected via the aryl-ether linkage (Figure S1); **B)** Graph displaying G-β-O-4$^{\alpha\text{-OX}}$ units *per* 100 C9 units against DDQ weight equivalents; Highlighted **H** atoms (and their corresponding cross-peaks within 2D HSQC analysis) were used for semi-quantification of units within lignin. Data repeated in triplicate, for standard error analysis, see Table S3. Native β-O-4 not shown for clarity.

This discussion is based on the data presented in Figure 3 in the manuscript and reproduced in Figure S8 above for ease of understanding this section. An initial increase in the quantity of oxidised β-O-4 units in the situations shown in Figures S8A(ii) or A(iii) in the lignin was observed (A(**G**)') (Fig. S8B light green bar increases on going from 0 – 0.5 wt. eqv. of DDQ (1 to 3 units *per* 100 C9 units). This was followed by a steady plateau in the size of the light green bar. Fully oxidised G-β-O-4 (A(**G**)") (in the situations shown in Figures S8A(iv) or A(v), dark green section of the bars in Figure S8B) was found to increase in quantity from 0 – 3 wt. eqv. of DDQ (see Manuscript Figure 2C for result at 3 wt. eqv. and Figure S6). Interestingly, unlike the situation for the β-O-4(S) units (light pink A(**S**)' signal), no decrease in the light green bar (A(**G**)') was seen as the dark green bar (A(**G**)") was increasing (*c.f.* Figure 3B changes in light pink and dark pink bars). A key observation noticed for most weight equivalents of DDQ used was that the dark green bar (A(**G**)") was larger than the light green bar (A(**G**)'). This led us to become interested in whether there were differences in reactivity for the G-**G**/S-**G** when compared to the S-**S**/G-**S** units. For this reason, we conducted time course analyses for the beech lignin oxidation at 80 °C and room temperature (S-**S**/G-**S** units discussed in manuscript).

G-G/S-G β-O-4 Results from the Time-course Study (S-**S**/G-**S** β-O-4 results discussed in manuscript)

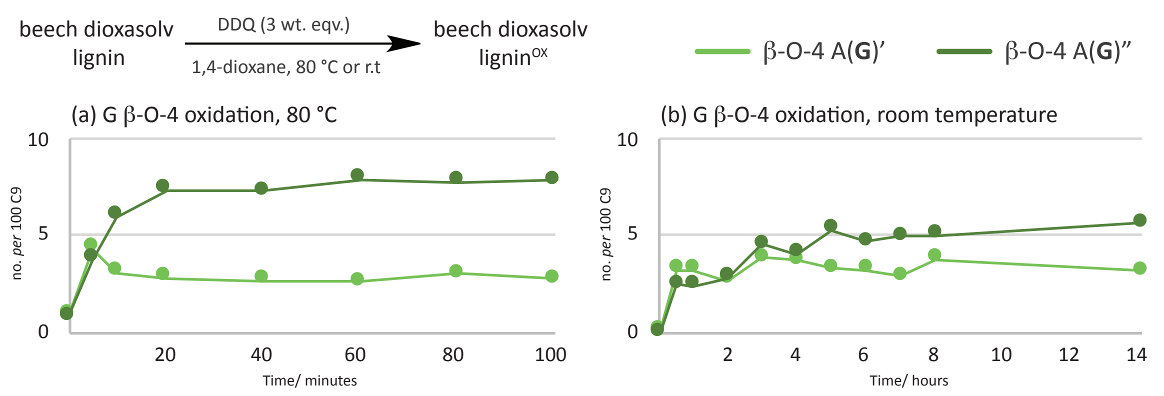

Figure S9: G-G/S-G- β-O-4 time-course analysis of beech lignin oxidation at: (a) 80 °C and; (b) room temperature. Data plotted as time vs. no *per* 100 C9 units, see full data in Tables S4 and S5.

Consumption of β-O-4 units have been omitted from Figure S9. The purpose of this time-course study was to examine differences in rates of S- and G-β-O-4 oxidation within beech lignin (for more discussion of S-β-O-4 oxidation, see manuscript). At 80 ^{o}C (Figure S9 (a)), within 10 minutes, almost equal amounts of A(**G**)' (light green) and A(**G**)" (dark green) units were observed (analogous to A(**S**)' and A(**S**)", Figure S10(a)(i)). By 20 minutes, a plateau of oxidation for G-β-O-4 units has been reached and no more A(**G**)" is observed. This was not the same for S-β-O-4 oxidation at 20 minutes as a gradual increase of A(**S**)" and corresponding decrease in A(**S**)' is still observed (Figure S10(a)(i)) over the next 80 minutes of the reaction. This provides further evidence that either (a) G-β-O-4 oxidation is faster than S-β-O-4 using these conditions or; (b) S-β-O-4 units could more often be found adjacent to other oxidizable units which are comparatively slower to be oxidised than β-O-4 units (for example, as is observed with the rapid loss of β-β resinol native units and slow increase in β-β derived alcohol and aldehyde units, Manuscript Figure 4 and Figure S10). To examine this in more detail, a competitive DDQ oxidation of model compounds **S1** and **S2** was conducted (Scheme S2).

Time Course Analysis of DDQ Oxidation of Beech lignin

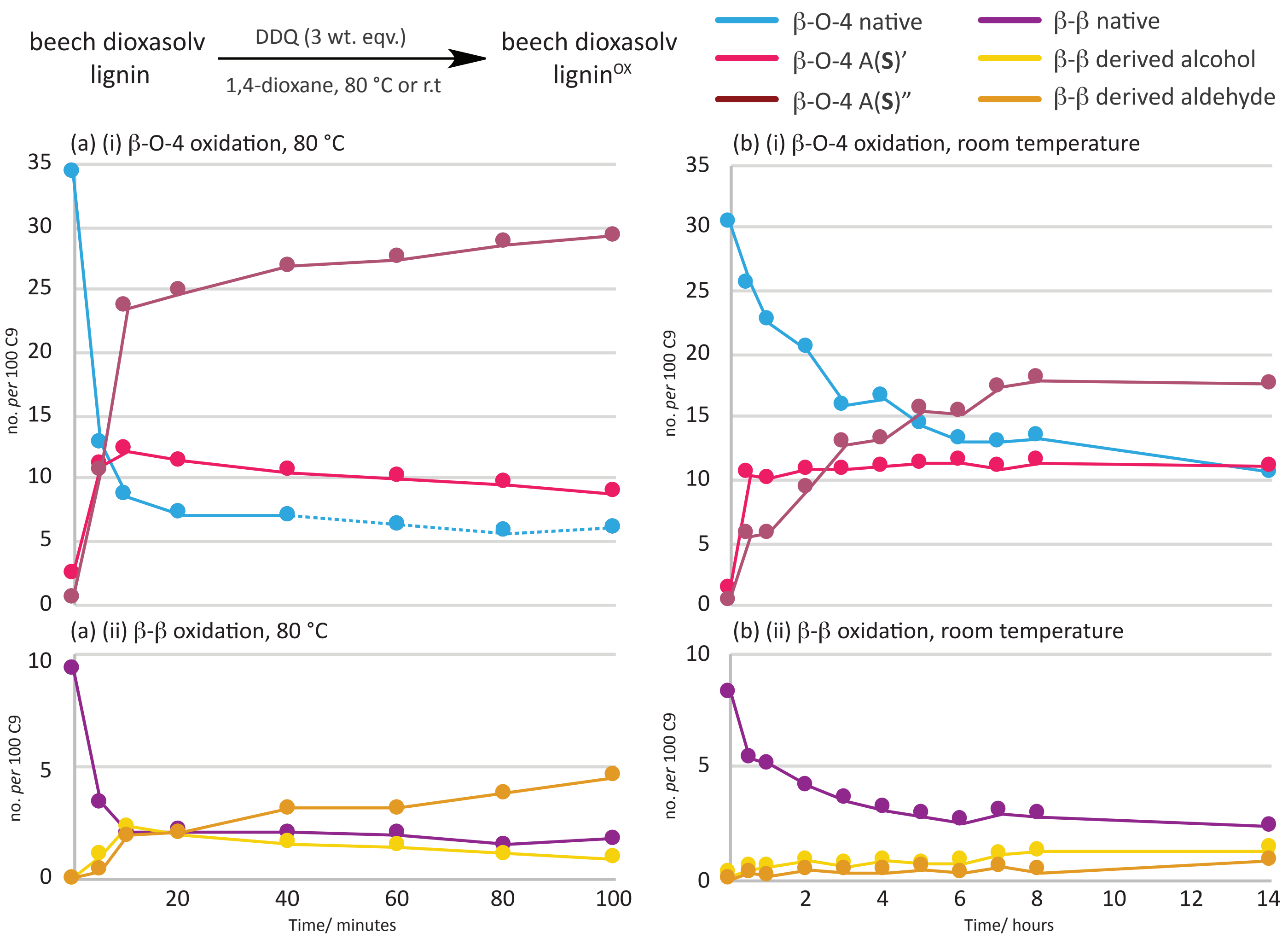

Figure S10: Graphical representations of a time-course analysis of DDQ oxidation of beech lignin at: **(a) 80 °C**; (i) β-O-4 oxidation; (ii) β-β oxidation; and; **(b) room temperature**; (i) β-O-4 oxidation; (ii) β-β oxidation. Data plotted as time vs. no *per* 100 C9 units, see full data in Table S4 and S5.

Table S4*:* Integral data from 2D HSQC NMR analysis of beech lignin DDQ oxidations (time-course study at 80 ^{o}C). In the first grouping of values ($S_{2,6}$, $S_{2,6}^{OX}$, G_2 (integral set arbitrarily to 100 units), G_2^{OX}), a value is obtained (in triplicate) for the total aromatic integral obtained from 2D HSQC NMR analysis using the integral regions described below Figure S5. The second grouping (β-O-4 native, β-O-4^{OX} A(S)', β-O-4^{OX} A(S)'', β-β resinol native, β-β derived alcohol, β-β derived aldehyde, LBHK, β-O-4^{OX} A(G)' and β-O-4^{OX} A(G)'') is reported as raw integrals and a *per* 100 C9 value obtained from using Equation 1 as is the norm.

**Time / minutes**	**0**	**5**	**10**	**20**	**40**	**60**	**80**	**100**
**$S_{2,6}$**	362.8	328.0	220.7	203.8	215.1	199.2	197.0	188.9
**$S_{2,6}^{OX}$**	22.0	212.6	358.6	404.3	471.5	485.8	523.7	538.6
**G_2**	100.0	100.0	100.0	100.0	100.0	100.0	100.0	100.0
**G_2^{OX}**	12.3	103.5	178.5	192.2	220.6	226.3	236.9	237.1
**Total Aromatics**	497.1	744.1	857.8	900.3	1007.1	1011.3	1057.6	1064.6
				**β-O-4 native**				
Raw integral		94.9	72.9	64.4	70.5	63.8	60.0	63.9
per 100 C9	**34.2**	**12.8**	**8.5**	**7.1**	**7.0**	**6.3**	**5.7**	**6.0**
				**β-O-4^{OX} A(S)'**				
Raw integral		81.7	104.0	102.3	106.2	101.4	100.6	93.6
per 100 C9	**2.3**	**11.0**	**12.1**	**11.4**	**10.5**	**10.0**	**9.5**	**8.8**
				**β-O-4^{OX} A(S)''**				
Raw integral		78.4	202.2	222.6	270.0	277.4	302.1	310.7
per 100 C9	**0.4**	**10.5**	**23.6**	**24.7**	**26.8**	**27.4**	**28.6**	**29.2**
				**β-β resinol native**				
Raw integral		25.5	17.3	18.9	20.7	19.7	15.8	18.8
per 100 C9	**9.4**	**3.4**	**2.0**	**2.1**	**2.1**	**1.9**	**1.5**	**1.8**
				**β-β derived alcohol**				
Raw integral		7.7	20.0	17.4	15.8	14.5	11.4	9.3

per 100 C9	**0.0**	**1.0**	**2.3**	**1.9**	**1.6**	**1.4**	**1.1**	**0.9**
**β-β derived aldehyde**								
Raw integral		2.2	16.2	18.4	31.3	31.8	40.0	48.6
per 100 C9	**0.0**	**0.3**	**1.9**	**2.0**	**3.1**	**3.1**	**3.8**	**4.6**
**LBHK**								
Raw integral		59.8	42.8	40.1	45.0	38.9	38.7	38.7
per 100 C9	**8.4**	**8.0**	**5.0**	**4.5**	**4.5**	**3.8**	**3.7**	**3.6**
**β-O-4^{OX} A(G)’**								
Raw integral		31.9	26.0	25.3	27.0	26.2	31.7	28.8
per 100 C9	**0.9**	**4.3**	**3.0**	**2.8**	**2.7**	**2.6**	**3.0**	**2.7**
**β-O-4^{OX} A(G)”**								
Raw integral		28.0	50.8	65.6	72.9	79.7	81.5	82.9
per 100 C9	**0.8**	**3.8**	**5.9**	**7.3**	**7.2**	**7.9**	**7.7**	**7.8**

Table S5: Integral data from 2D HSQC NMR analysis of beech lignin DDQ oxidations (time-course study at room temperature). In the first grouping of values ($S_{2,6}$, $S_{2,6}^{OX}$, G_2 (integral set arbitrarily to 100 units), G_2^{OX}), a value is obtained for the total aromatic integral obtained from 2D HSQC NMR analysis using the integral regions described below Figure S5. The second grouping (β-O-4 native, β-O-4^{OX} A(S)', β-O-4^{OX} A(S)'', β-β resinol native, β-β derived alcohol, β-β derived aldehyde, LBHK, β-O-4^{OX} A(G)' and β-O-4^{OX} A(G)'') is reported as raw integrals and a *per* 100 C9 value obtained from using Equation 1 as is the norm.

Time / hours	0	0.5	1	2	3	4	5	6	7	8	14
$S_{2,6}$	438.8	326.4	318.6	284.5	265.9	309.3	259.7	287.5	268.0	258.0	257.8
$S_{2,6}^{OX}$	13.3	141.5	142.6	176.8	226.8	263.3	261.3	279.5	297.7	298.7	315.5
G_2	100.0	100.0	100.0	100.0	100.0	100.0	100.0	100.0	100.0	100.0	100.0
G_2^{OX}	10.7	83.0	72.4	81.7	98.7	107.5	110.4	109.1	123.1	115.2	123.0
**Total Aromatics**	562.7	650.9	633.7	642.9	691.3	780.1	731.3	776.1	788.8	771.8	796.2
	**β-O-4 native**										
Raw integral	171.2	166.8	142.7	130.8	109.7	129.1	104.8	101.6	102.8	102.7	84.6
per 100 C9	**30.4**	**25.6**	**22.5**	**20.4**	**15.9**	**16.6**	**14.3**	**13.1**	**13.0**	**13.3**	**10.6**
	**β-O-4^{OX} A(S)'**										
Raw integral	7.2	68.1	64.0	69.4	75.1	86.6	82.5	88.9	86.8	88.3	88.5
per 100 C9	**1.3**	**10.5**	**10.1**	**10.8**	**10.9**	**11.1**	**11.3**	**11.5**	**11.0**	**11.4**	**11.1**
	**β-O-4^{OX} A(S)''**										
	2.1	36.6	36.4	59.1	88.8	102.9	113.4	118.5	137.0	138.2	140.2
per 100 C9	**0.4**	**5.6**	**5.7**	**9.2**	**12.8**	**13.2**	**15.5**	**15.3**	**17.4**	**17.9**	**17.6**
	**β-β native**										
Raw integral	45.8	34.8	32.0	26.6	24.0	24.0	20.3	19.6	23.2	21.9	18.3
per 100 C9	**8.1**	**5.4**	**5.0**	**4.1**	**3.5**	**3.1**	**2.8**	**2.5**	**2.9**	**2.8**	**2.3**
	**β-β derived alcohol**										
Raw integral	1.1	3.3	3.4	5.4	4.2	6.4	5.2	5.9	8.6	9.3	10.7

per 100 C9	**0.2**	**0.5**	**0.5**	**0.8**	**0.6**	**0.8**	**0.7**	**0.8**	**1.1**	**1.2**	**1.3**
**β-β derived aldehyde**											
Raw integral	0.2	2.0	1.0	2.8	2.6	2.9	3.8	2.0	4.3	2.8	6.7
per 100 C9	**0.0**	**0.3**	**0.2**	**0.4**	**0.4**	**0.4**	**0.5**	**0.3**	**0.5**	**0.4**	**0.8**
**LBHK**											
Raw integral	40.2	60.3	49.9	47.8	67.2	73.9	70.1	70.1	71.7	64.9	70.9
per 100 C9	**7.1**	**9.3**	**7.9**	**7.4**	**9.7**	**9.5**	**9.6**	**9.0**	**9.1**	**8.4**	**8.9**
**β-O-4^{OX} A(G)’**											
Raw integral	0.9	20.8	20.2	17.0	26.5	28.9	23.9	24.9	22.7	28.9	25.2
per 100 C9	**0.2**	**3.2**	**3.2**	**2.6**	**3.8**	**3.7**	**3.3**	**3.2**	**2.9**	**3.7**	**3.2**
**β-O-4^{OX} A(G)”**											
Raw integral	0.0	16.0	15.0	18.2	31.2	31.5	38.2	36.0	38.8	38.7	44.8
per 100 C9	**0.0**	**2.5**	**2.4**	**2.8**	**4.5**	**4.0**	**5.2**	**4.6**	**4.9**	**5.0**	**5.6**

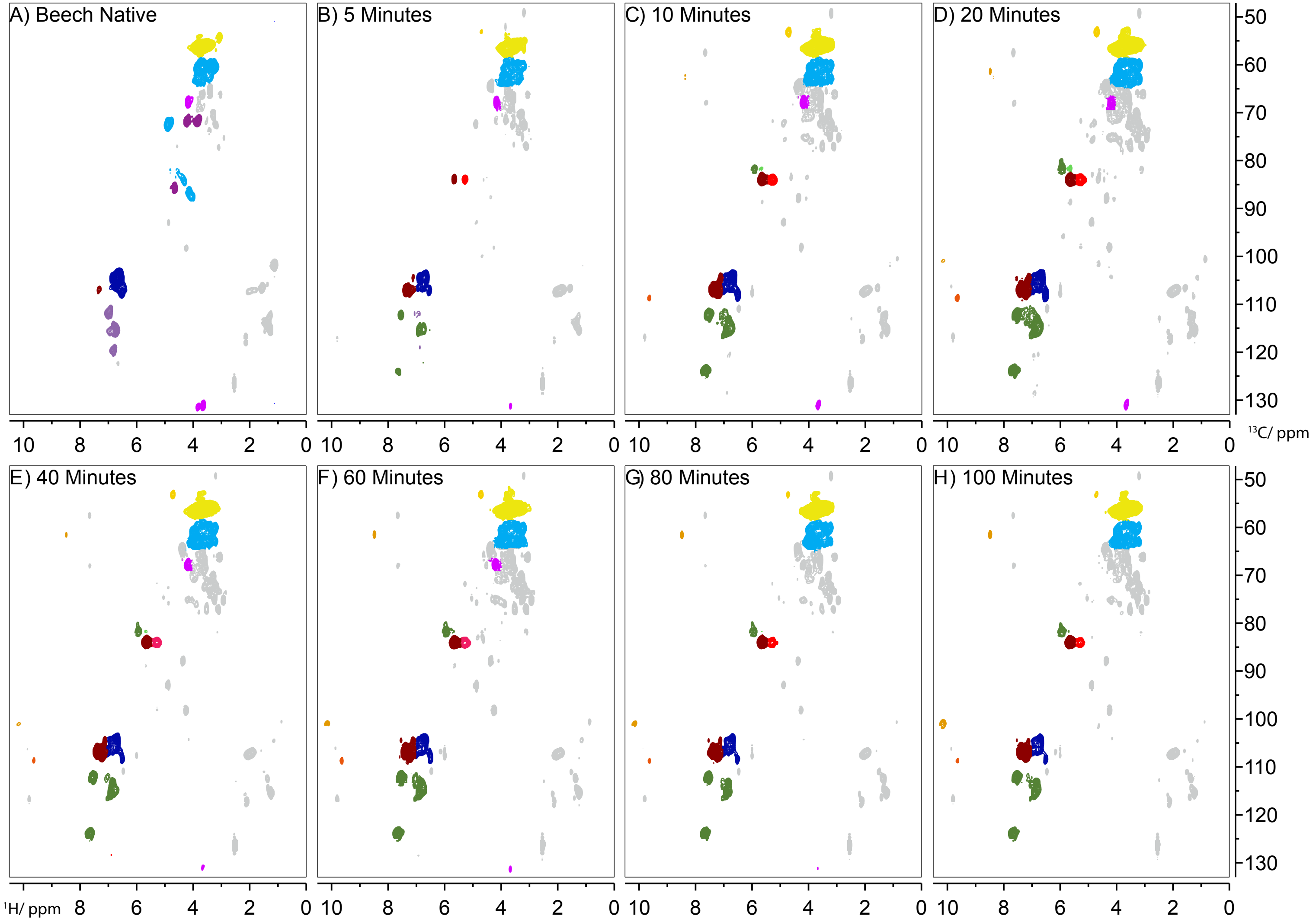

Figure S11: 2D HSQC NMR analysis (700 MHz, d_6-DMSO) of beech dioxasolv lignin (A-H, 0 -100 minutes) during time course analysis at 80 °C. Reaction conducted using 3 wt. eqv. of DDQ. For coloured contour assignment, see Figure S6.

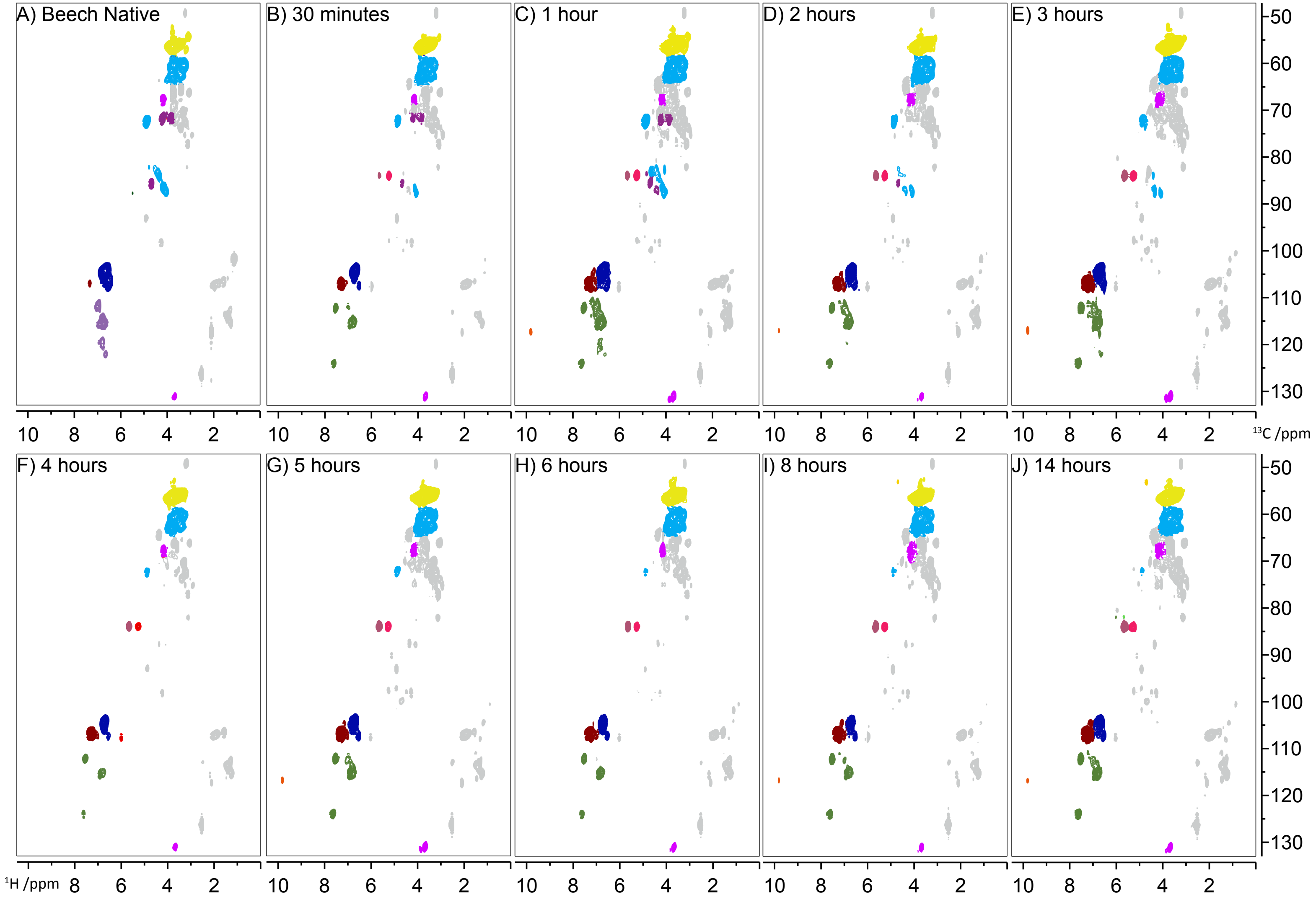

Figure S12: 2D HSQC NMR analysis (700 MHz, d_6-DMSO) of beech dioxasolv lignin during time course analysis of DDQ oxidation at room temperature. For coloured contour assignment, see Figure S6.

G- vs. S- β-O-4 Competitive Oxidation of Model Compounds

To examine whether G-G or S-S β-O-4 units convert faster under our reaction conditions, a competitive model study with G-G and S-S β-O-4 models **S1** and **S2** was conducted. A mixture of 1: 2.5 of **S1** and **S2** (25.0 mg and 75.0 mg respectively representing a ~1: 2.5 mixture) were prepared and subjected to the following conditions: DDQ (60 mg, 1 eqv.), 1,4-dioxane (4 mL, 25 mg/ mL), 80 °C. 400 µL aliquots were taken over an 8-minute period and each aliquot quenched through addition of 200 µL $NaHCO_3$ (saturated solution). Samples were transferred to a GeneVac® EZ-2 and concentrated under reduced pressure. To each sample was added 1000 µL of an internal standard solution in $CDCl_3$ (I.S: Sesamol >99%, 52.6 mg accurately weighed out into 50 mL of $CDCl_3$ in a volumetric flask), vortexed to homogenise the solution and centrifuged at 5000 rpm for 5 minutes. Samples were transferred to NMR tubes and analysed by quantitative ^{1}H NMR using the standard pulse sequence from the Bruker library (*zg*) with an interscan delay (D1) of 30 seconds.

NB. Whilst a 1: 2.5 mixture of **S1** to **S2** does not represent the exact S:G ratio found in the hardwood lignin we were using, enough of **S1** was needed to ensure signal was acquired by NMR.

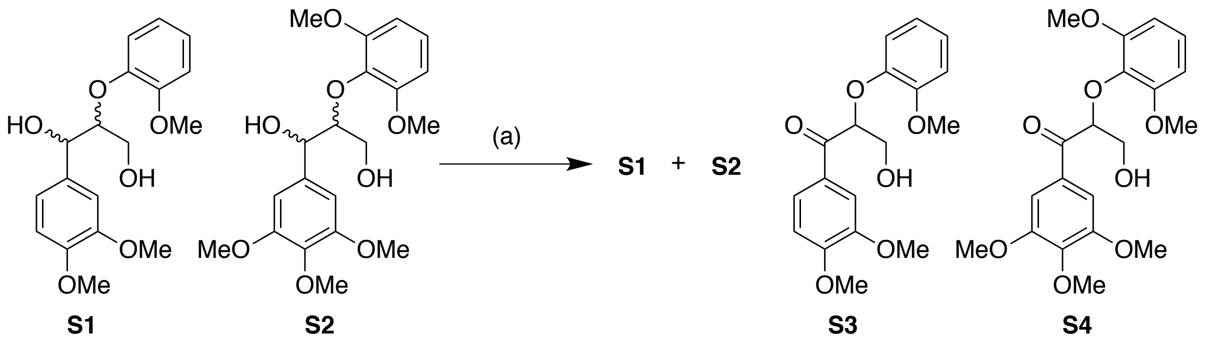

Scheme S2: Competitive DDQ oxidation of β-O-4 models **S1** and **S2.** (a) DDQ (1.0 eqv.), 1,4-dioxane (25 mg/mL), 80 °C, 8 minutes.

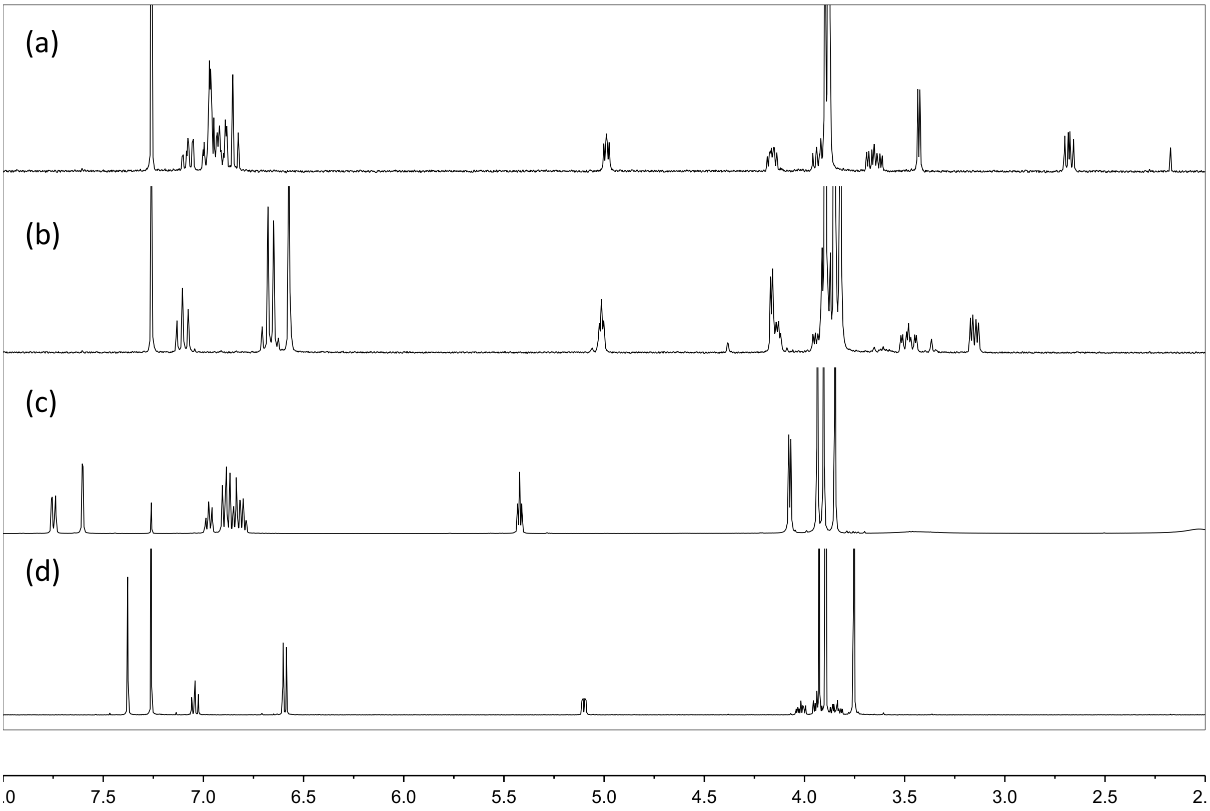

Figure S13: ^{1}H NMR spectra ($CDCl_3$) of models **S1**-**S4** (a)-(d).

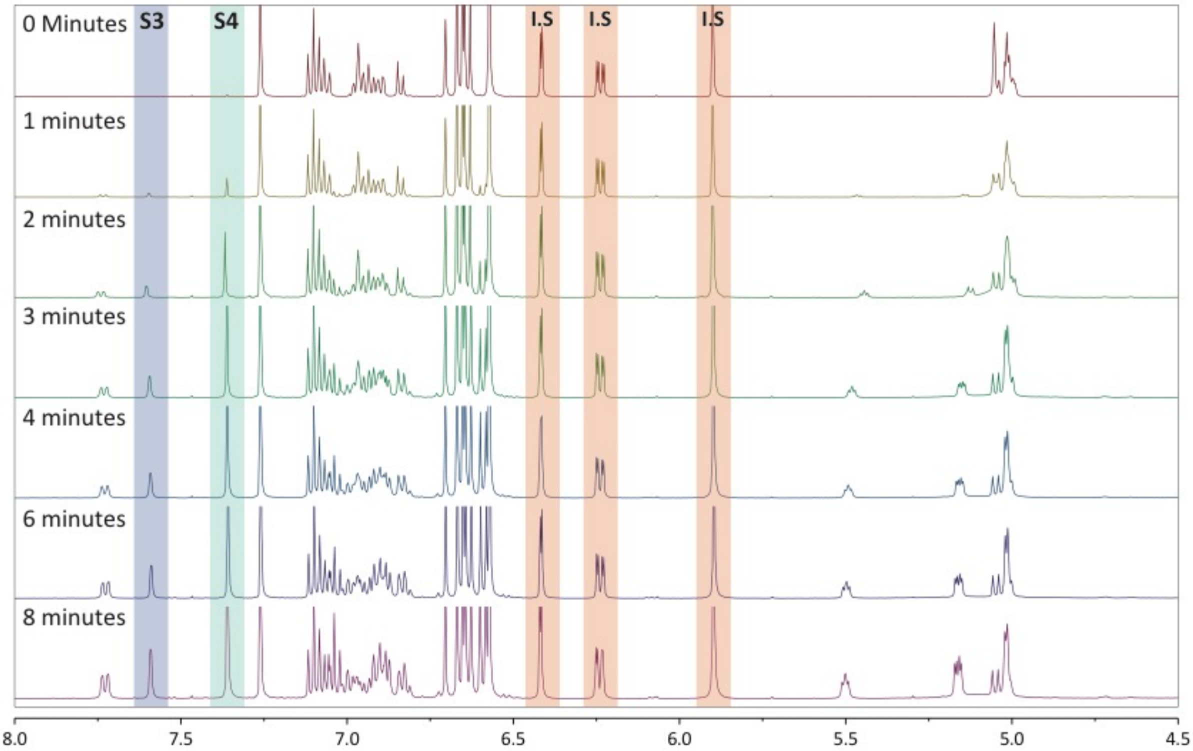

Figure S14: Example of ^{1}H NMR data from DDQ model reaction of **S1** and **S2** at 80 °C. Peak at 6.25 ppm corresponds to one of the signals in the internal standard, Sesamol.

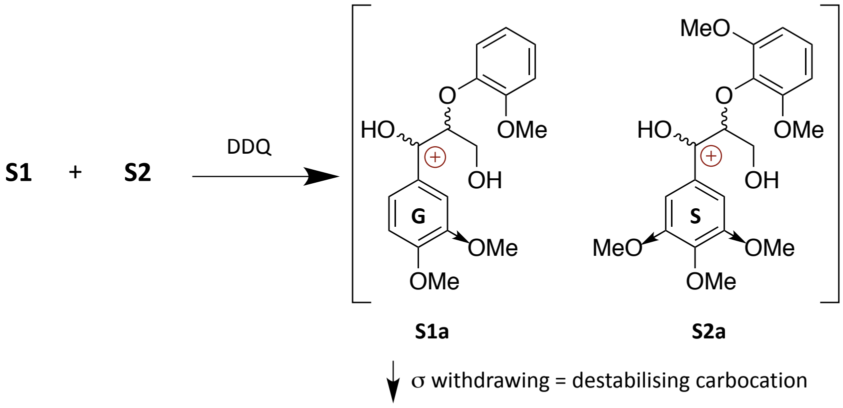

Figure S15: DDQ oxidation of **S1** and **S2** leads to proposed intermediate carbocations **S1a** and **S2a**.

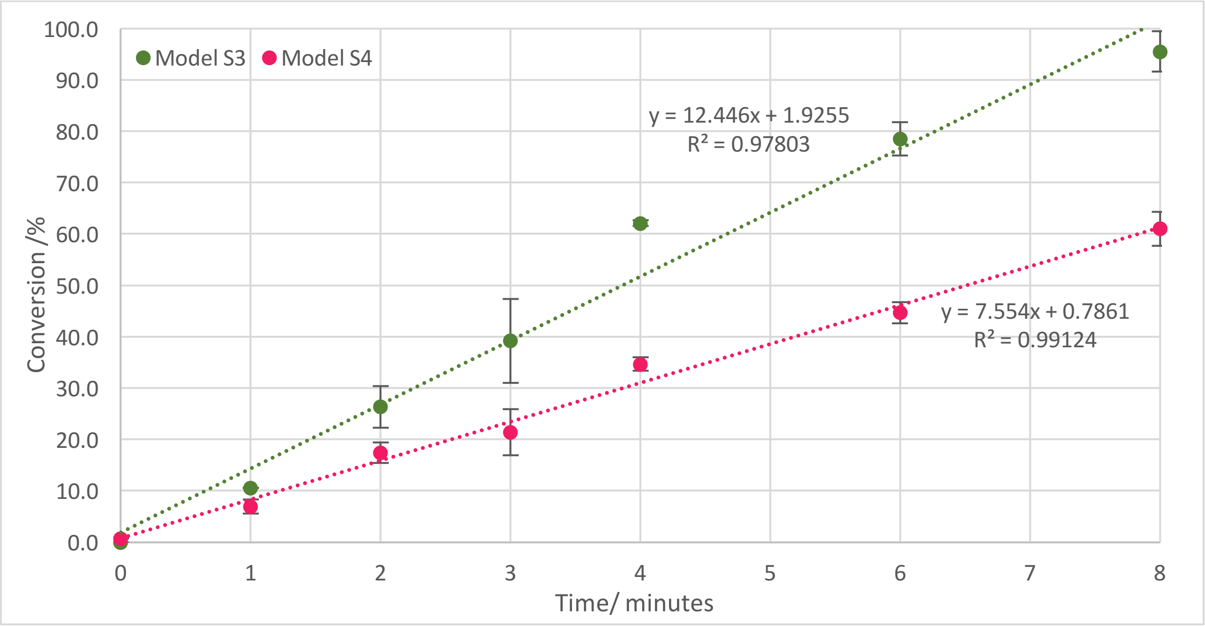

Figure S16: Graphical representation of DDQ oxidation of models **S1** and **S2** to yield **S3** (green line) and **S4** (pink line) as shown in Scheme S2.

From the competitive DDQ oxidation reaction with models **S1** and **S2** (Scheme S2), it would be predicted that at any given time point, the conversion of **S1**, the G-G β-O-4 model, would be higher due to better stability of the intermediate carbocation (**S1a** vs. **S2a**, Figure S15), than **S2**, the S-S β-O-4 model. From the time-course analysis data (Figure S16), it is observable that at any time point, the oxidation of model **S1** to model **S3** (green line) reaches higher conversion than model **S2** to **S4** (pink line). This evidence supports the observation that G-β-O-4 oxidation is faster than S-β-O-4 with much lower conversions observed over time. A low rate of S-β-O-4 oxidation may also be impacted by the rapid consumption of DDQ from G-β-O-4 oxidation.

Study of LBHK model compounds with DDQ

For synthesis of Models **1a** and **1b** see Reference [7]

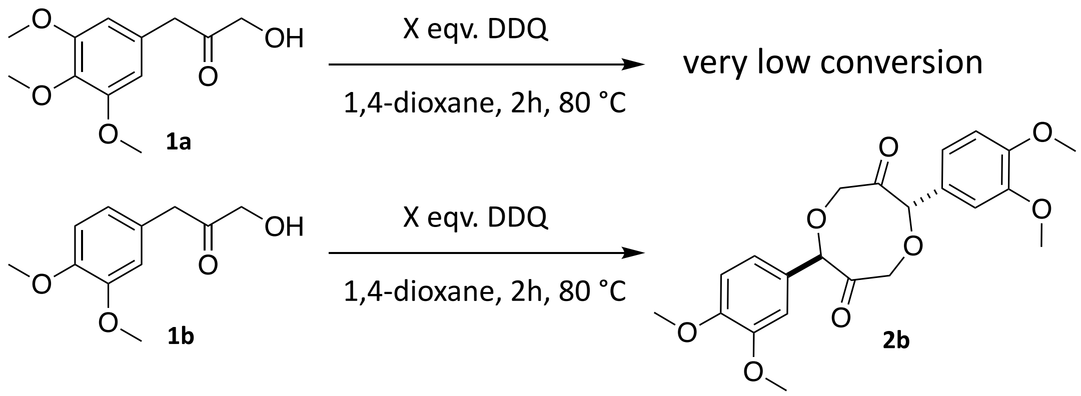

Scheme S3: Reactivity of LBHK model **1a/b** when subjected to DDQ oxidation conditions analogous to beech lignin$^{\alpha\text{-OX}}$ generation.

General reaction procedure: To a vial is added model compound **1a/b** (25 mg) in 1,4-dioxane (1 mL) is added DDQ (X eqv.) and heated to 80 °C for 2 hours. The reaction is cooled, filtered through celite and concentrated *in vacuo* to give a crude product for NMR analysis.

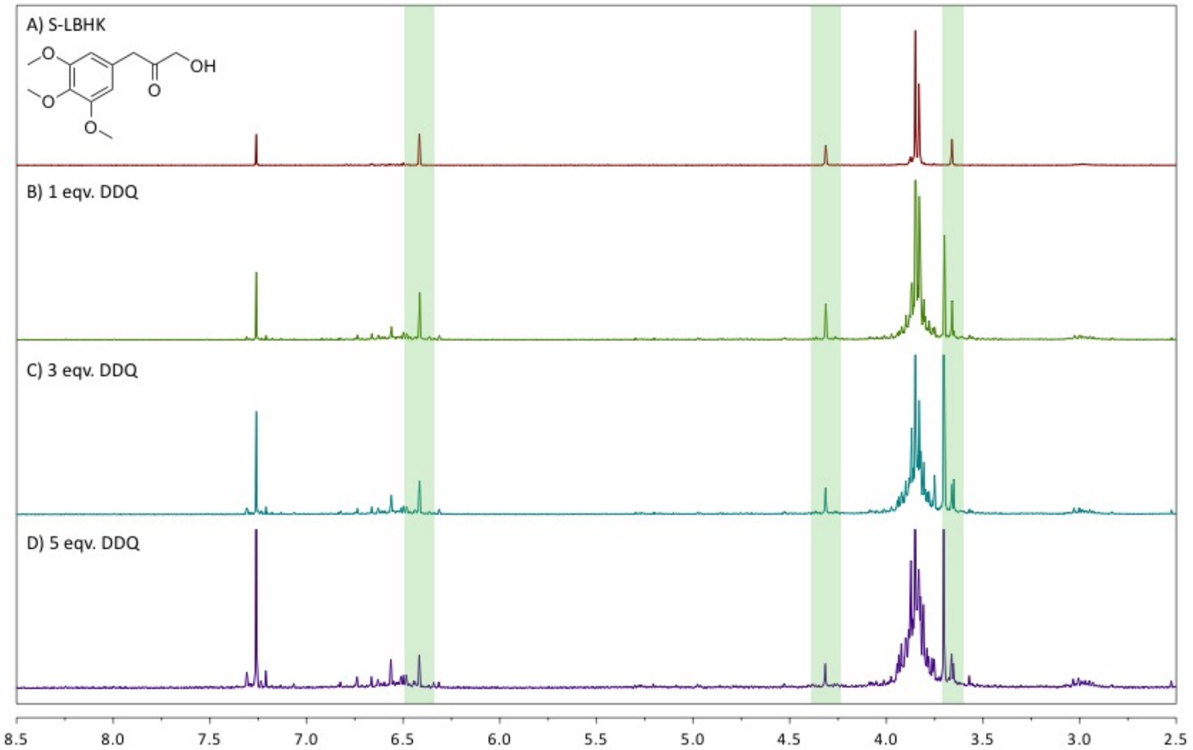

Figure S17a: S-LBHK DDQ model study: ^{1}H NMR spectra from **A**) LBHK model **1a** (S-LBHK); and crude reaction mixture with **B**) 1 eqv. DDQ; **C**) 3 eqv. DDQ and; **D**) 5 eqv. DDQ.

From analysis of S-LBHK **1a** reactions (Figure S17a), decomposition of **1a** with increasing numbers of equivalents of DDQ does appear to be occurring but to no single product.

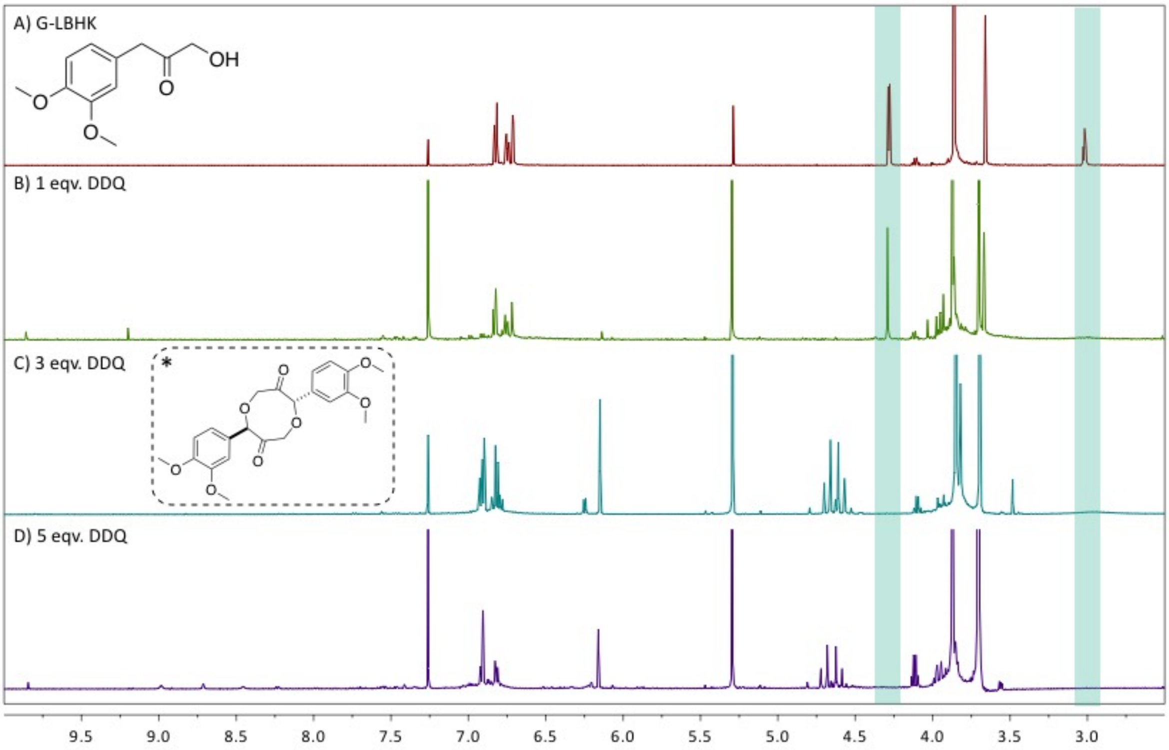

Figure S17b: G-LBHK DDQ model study: ^{1}H NMR spectra from **A**) LBHK model **1b** (G-LBHK); and crude reaction mixture with **B**) 1 eqv. DDQ; **C**) 3 eqv. DDQ and; **D**) 5 eqv. DDQ. *Proposed structure

From analysis of G-LBHK **1b** reactions (Figure S17b), the reaction with 1 eqv. of DDQ (Figure S17b: B), no/ very little conversion of **1b** is observed. With increasing numbers of DDQ (3-5 eqv.) (Figure S17b: C-D), full conversion of **1b** is observed to one major species. Attempts to purify the material generated here (silica/ alumina chromatography) led to decomposition of the product and any additional work-up steps caused decomposition (*e.g.* quenching of excess DDQ with $NaHCO_3$). Crude analysis of the mixture generated from 3 eqv. of DDQ led us to a proposed structure shown in Figure S17b: C.

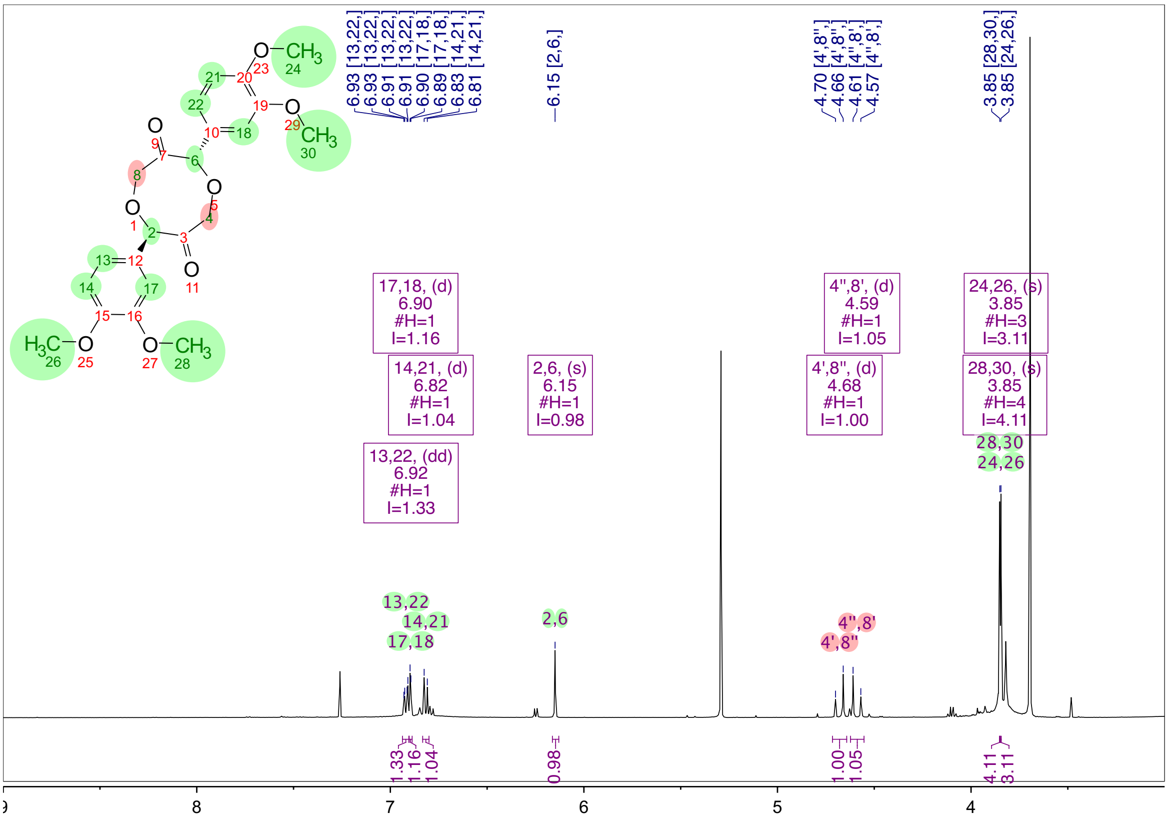

Figure S17c: ^{1}H NMR analysis of crude reaction mixture from the reaction of **1b** with 3 eqv. of DDQ yielding proposed 8-membered ring structure **2b**.

From the ^{1}H NMR (Figure S17c) (and COSY analysis, not shown) it was found that H4/4' and H8/8' are found on the same carbon with high geminal *J* values of 20 Hz. There is one major set of signals present suggesting the molecule is C2 symmetric (possibly only one diastereoisomer present). 2D multiplicity-edited HSQC NMR analysis (Figure S17d) confirms that the singlet peak at 6.15 ppm is a CH and the doublets at 4.68 and 4.59 ppm is a CH_2. The ^{13}C NMR (Figure S17e) confirms a carbonyl is still present at 204.7 ppm (similar to the starting material[7]) and corresponding HMBC analysis (Figure S17f) confirms that this carbonyl still observes H4/8 and H2/6 (and H4/8 and H2/6 observe each other) suggesting no change in the backbone to this structure. H2/6 still observes *3J* coupling to C13/22 and C17/18 again confirming no change. Strong de-shielding of H2/6 by *ca.* 2 ppm could be explained by it now being bound to an oxygen atom and adjacency to a ketone. The effect of the macrocycle may also be influencing the chemical shifts of these H-atoms.

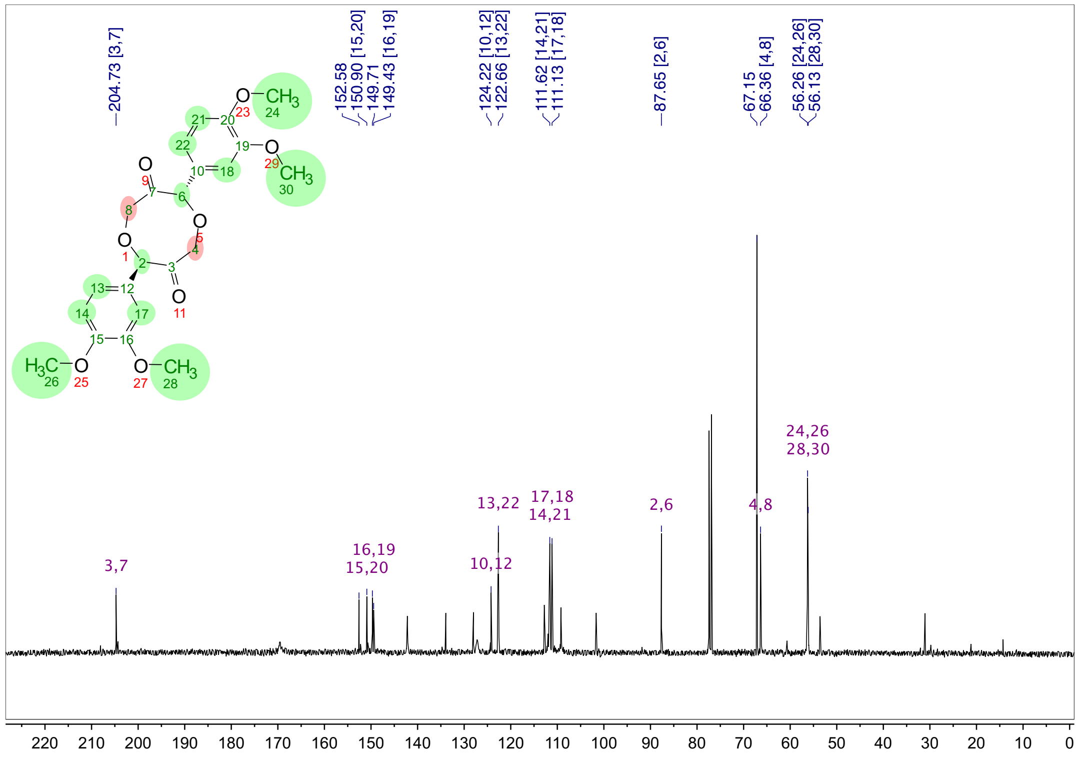

Figure S17d: ^{13}C NMR analysis of crude reaction mixture from the reaction of **1b** with 3 eqv. of DDQ yielding proposed 8-membered ring structure **2b**.

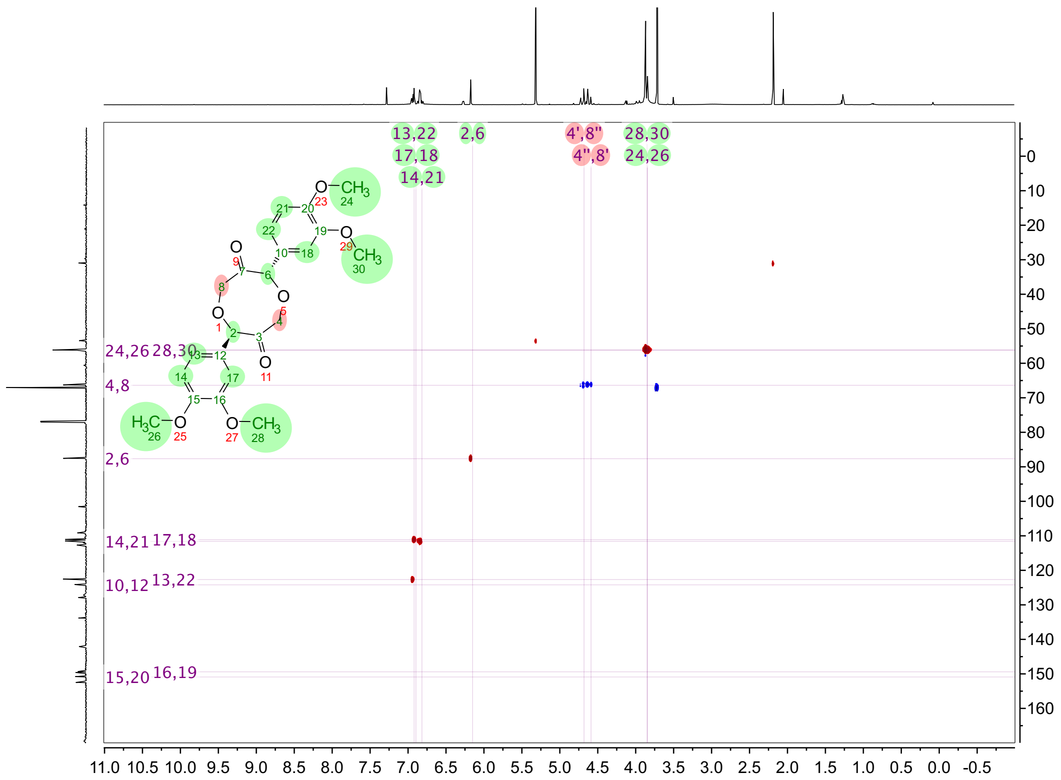

Figure S17e: 2D HSQC NMR analysis of crude reaction mixture from the reaction of **1b** with 3 eqv. of DDQ yielding proposed 8-membered ring structure **2b**.

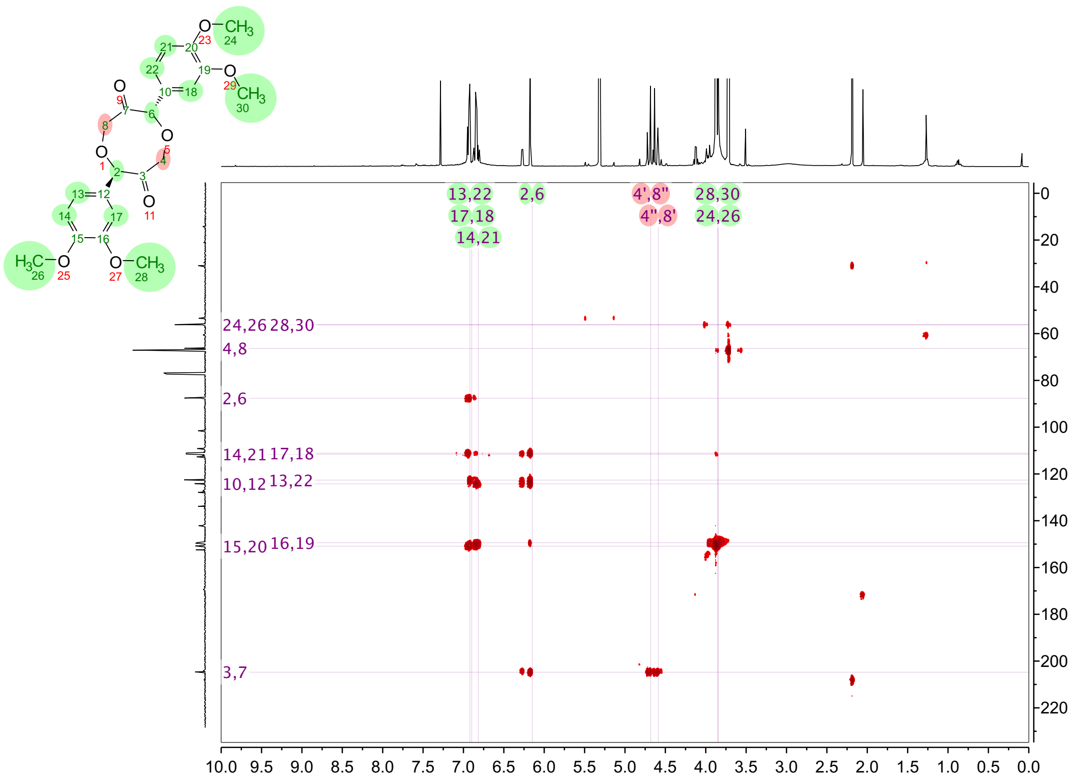

Figure S17f: 2D HSQC NMR analysis of crude reaction mixture from the reaction of **1b** with 3 eqv. of DDQ yielding proposed 8-membered ring structure **2b**.

HRMS analysis led to the following: **HRMS** (ESI+) *m/z* [M + 2H$^+$] calcd. for $C_{22}H_{26}O_8^{2+}$ 209.0808, found 209.0809. A possible DDQ mechanism involving formation of a benzylic cation *via* H-abstraction followed by intermolecular trapping with the primary alcohol of another molecule of **1b** (Figure S17g **A**) examines how **2b** could be formed. A possible fragmentation in the MS could have led to the found mass of 209.0809 Da (Figure S17g **B**). Additional HRMS analysis led to the following result: **HRMS** (ASAP+) *m/z* [M + H$^+$] calcd. for $C_{22}H_{25}O_8^+$ 417.1550, found 417.1549.

IR analysis led to the following result: **IR** (FTIR)ν_{max}: 2966, 2939, 1734, 1695, 1681, 1560, 1514, 1442 cm^{-1}; the carbonyl shift is consistent for a carbonyl in an 8-membered ring structure.

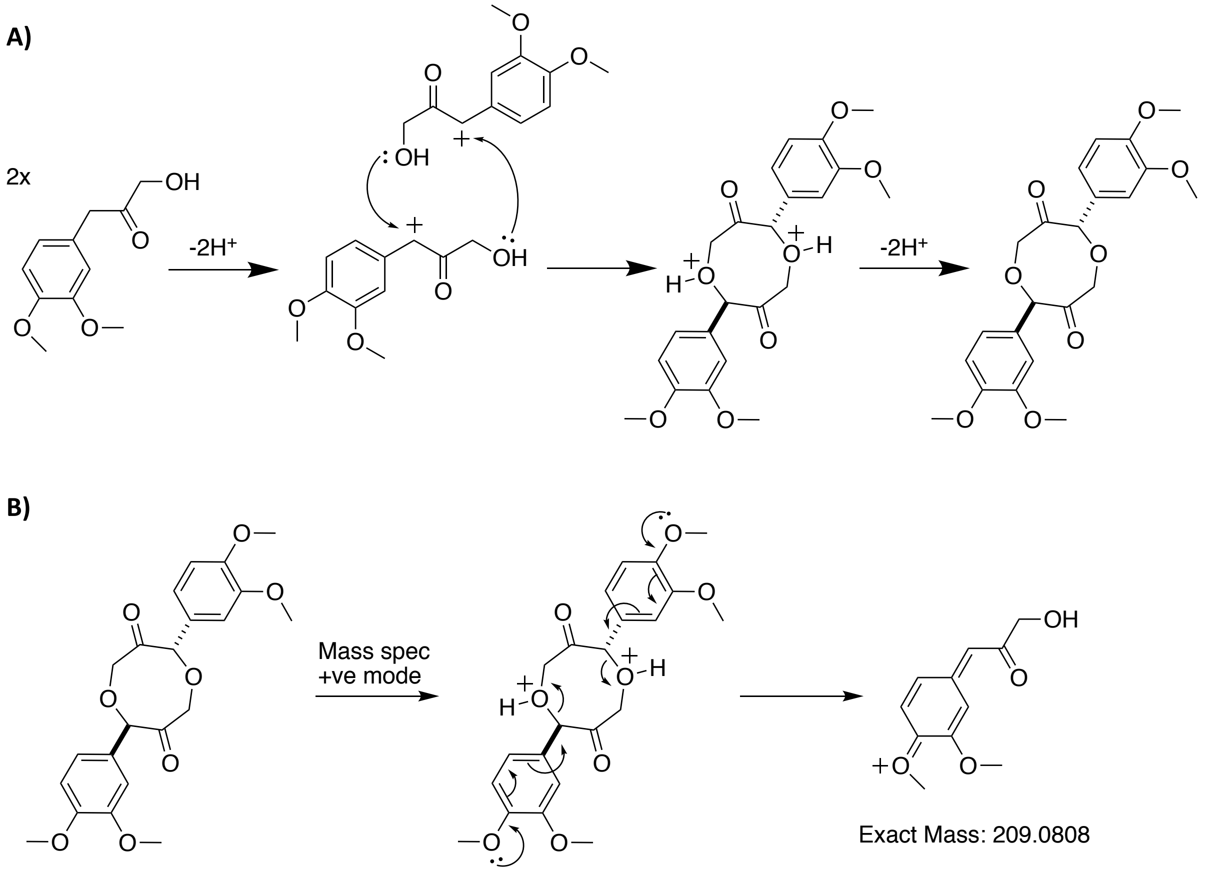

Figure 17g: **A**) Proposed mechanism leading to the formation of **2b** using DDQ and; **B**) possible rationale for HRMS result.

Pseudo-2D DOSY NMR Analysis of Compound 2b

Initial analysis of **2b** led to two possible structures being proposed, a 4-membered ring and an 8-membered ring (Figure S17h). In an attempt to distinguish between the two structures, molecular weight prediction using DOSY NMR (a non-destructive technique) was conducted based on recent work by *Morris et al.*[8]

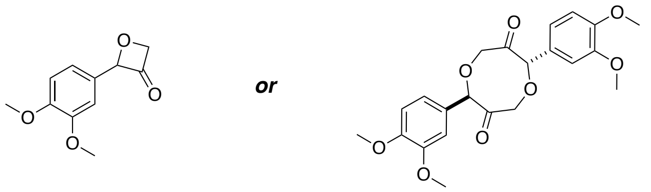

Figure S17h: Two proposed structures of compound **2b**.

A known compound, **1b**, was analysed by DOSY NMR alongside a doped sample of **1b** with a crude mixture of **2b**. The crude reaction mixture (containing 10 mg of **2b**) was dissolved in 0.7 mL of d_4-MeOD and was doped with **1b** (10 mg). DOSY measurements were carried out on Bruker Avance III 700 MHz spectrometer equipped with a $^{1}H/^{13}C/^{15}N$ TCI cryoprobe (Prodigy) using the *ledbpgp2s* pulse sequence from the Bruker library. Diffusion parameters Δ and δ were optimised prior to the 2D experiment. 32 magnetic field gradient amplitudes from 5.9 to 57.9 G cm^{-1} were used and incremented in equal steps. The probe temperature was maintained at 298 K and the sample was left to thermally equilibrate for 15 minutes inside the magnet prior to acquisition. Molecular weights of the solutes were predicted as previously reported by Morris *et al.*[8]

2D DOSY NMR analysis (Figure S17i) of model **1b** (highlighted in green) yielded a predicted molecular weight of 188.9 g mol^{-1} (actual M_W of 210.2 g mol^{-1}). 2D DOSY NMR analysis of model **2b** (Figure S17i, highlighted in red) doped with **1b** (green) showed a major difference in predicted molecular weights of **1b** and **2b**. Compound **2b** was predicted to have a molecular weight of 384.2 g mol^{-1} (actual M_W of 416.4 g mol^{-1}). This result suggests the 8-membered ring is more likely to be present in solution than the 4-membered ring. Unfortunately, due to the instability of the compound, further analysis (*e.g.* X-ray crystallographic analysis) at this time is not possible.

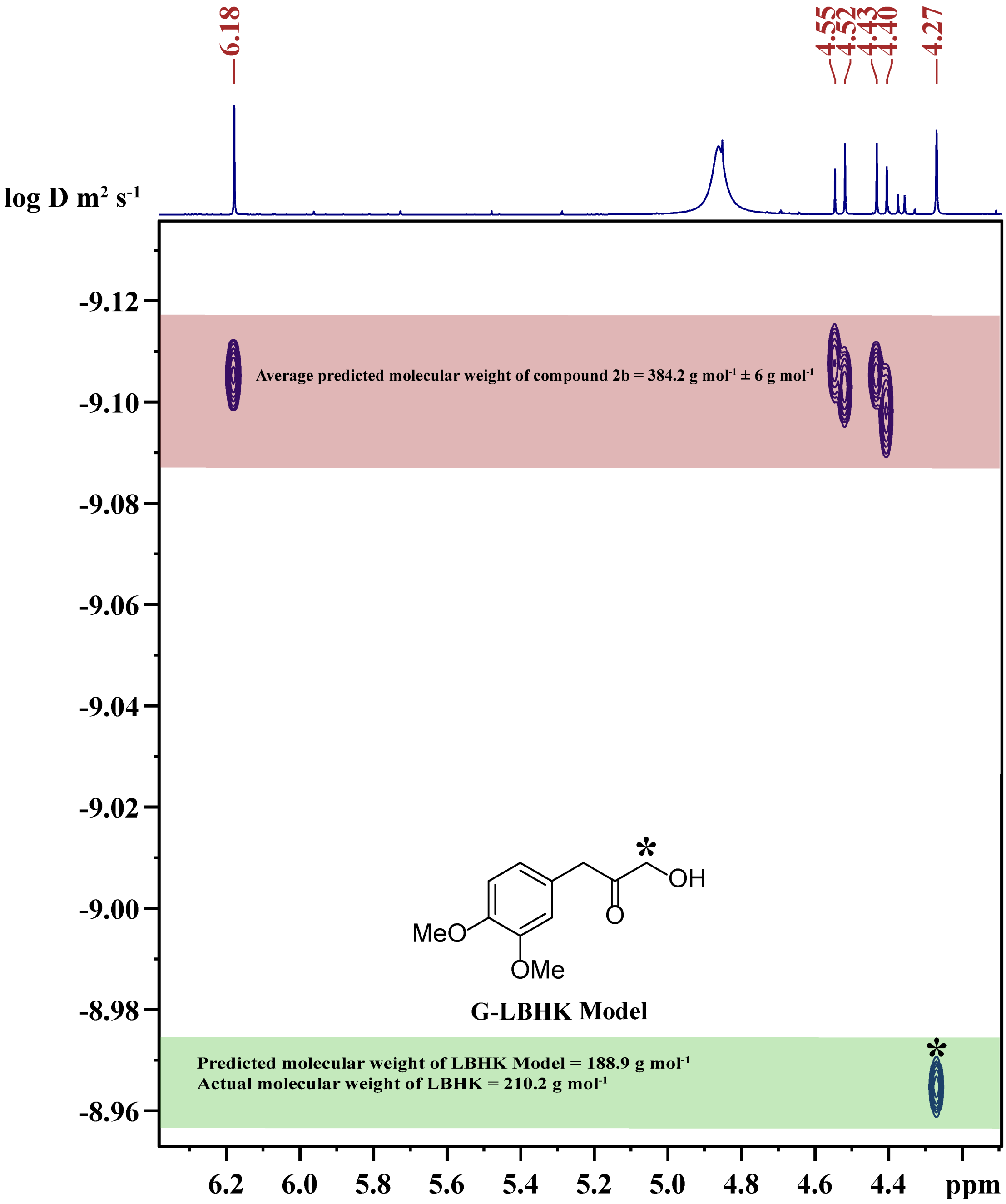

Figure S17i: *Pseudo*-2D DOSY NMR analysis of G-LBHK model (**1b**) (diffusion coefficient highlighted in green) and compound **2b** (diffusion coefficient highlighted in red). * highlighted position on model **1b** refers to the signal in the ^{1}H NMR spectrum used to acquire the diffusion coefficient of that molecule. Only one peak was selected on **1b** due to overlap of other peaks.

Synthesis of β-O-4-LBHK advanced model compounds

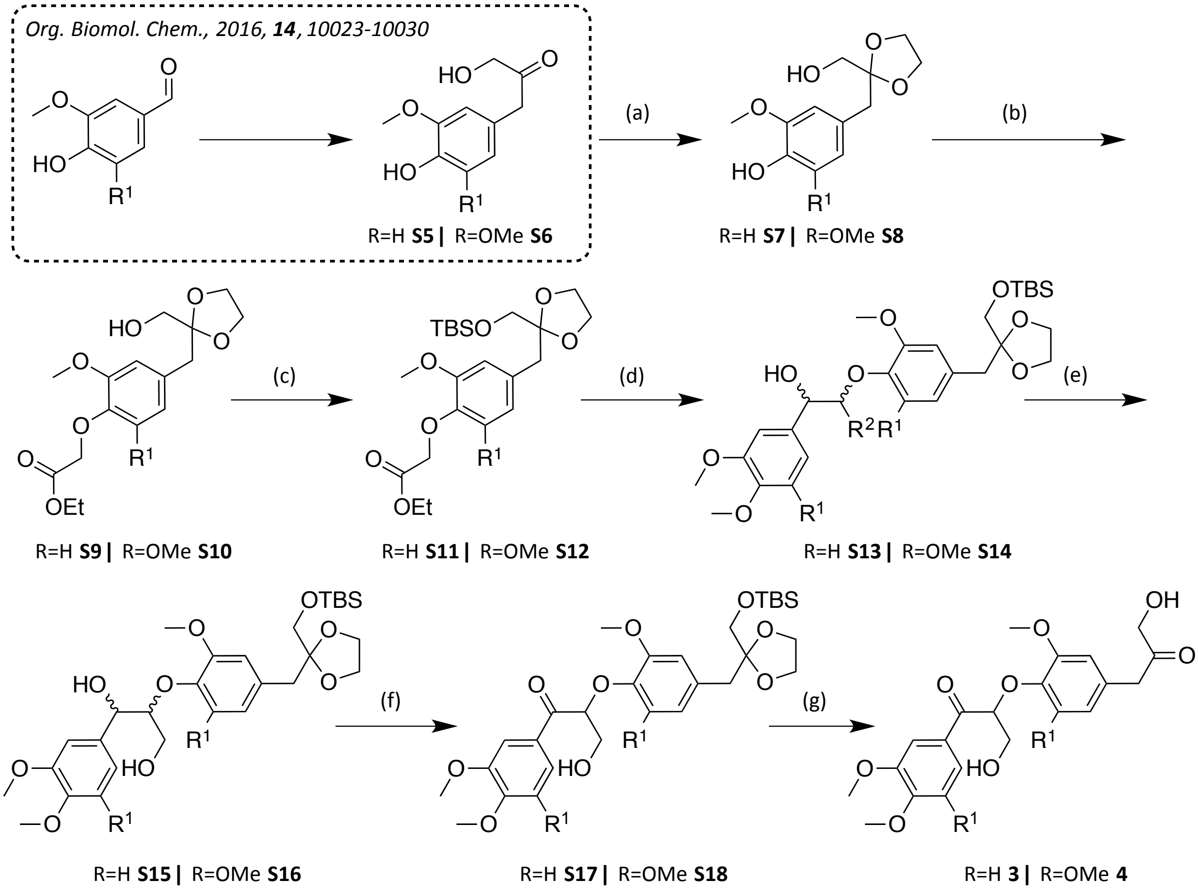

Scheme S4: Synthesis of β-O-4-LBHK model compounds **3** and **4**. Reaction conditions: (a) CSA, 1,2-ethanediol, toluene, reflux, 2h; (b) ethyl bromoacetate, K_2CO_3, acetone, reflux, 1h; (c) TBS-Cl, imidazole, DMAP, dry DCM, r.t. 16h; (d) LDA, dry THF, 3,4-dimethoxybenzaldehyde (for **S13**), 3,4,5-trimethoxybenzaldehyde (for **S14**), -78 °C, 2h; (e) $NaBH_4$, EtOH, MeOH, r.t., 16h; (f) DDQ, DCM, r.t. 16h; (g) I_2, acetone, r.t. 16h. $R^2 = CO_2Et$.

ethyl 2-(4-((2-(((*tert*-butyldimethylsilyl)oxy)methyl)-1,3-dioxolan-2-yl)methyl)-2-methoxyphenoxy)acetate (**S11**)

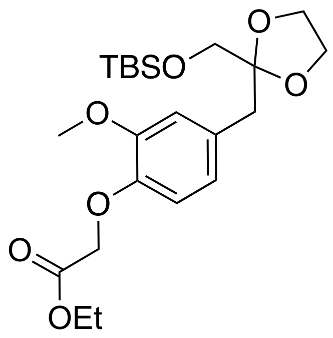

To a stirred solution of **S5** (0.356 g, 1.81 mmol) in toluene (20 mL) is added campher-10-sulfonic acid (0.021 g, 0.05 eqv.) and ethylene glycol (0.50 mL, 5.00 eqv.) and heated at reflux for 2 hours. Upon completion, mixture is cooled and quenched with H_2O, washed with brine, dried over $MgSO_4$ and concentrated *in vacuo* to give crude **S7**. To a solution of crude **S7** (0.436 g, 1.81 mmol) in acetone (30 mL) is added K_2CO_3 (0.301 g, 1.20 eqv.) and ethyl bromoacetate (0.24 mL, 1.2 eqv.) and heated to reflux for 1 hour. Upon completion, mixture is filtered through celite and concentrated *in vacuo* to yield crude **S9** as a yellow oil. To a solution of crude **S9** (0.592 g, 1.81 mmol) in DCM (10 mL) is added imidazole (0.247 g, 2.00 eqv.), DMAP (0.011 g, 0.05 eqv.) and TBS-Cl (0.348 g, 1.30 eqv.) and stirred for 16 hours. Upon completion, reaction is quenched with NH_4Cl (saturated solution), washed with H_2O, brine, dried over $MgSO_4$ and concentrated *in vacuo*. Crude **S11** is purified by flash column chromatography (10-30% ethyl acetate in petroleum ether) to give pure **S11** as a colourless oil (0.399 g, 50% over 3 steps).

IR (FTIR)ν_{max}: 2954, 1761 1734, 1606, 1514, 1463 cm^{-1}; **HRMS** (NSI+) *m/z* [M + NH_4^+] calcd. for $C_{22}H_{40}O_7Si^+$ 458.2569, found 458.2558; **^{1}H NMR** (700 MHz, Chloroform-*d*) δ = 6.89 (d, *J*=1.9, 1H), 6.78 (dd, *J*=8.3, 1.9, 1H), 6.73 (d, *J*=8.2, 1H), 4.65 (s, 2H), 4.25 (q, *J*=7.1, 2H), 3.88 – 3.84 (m, 5H), 3.65 – 3.61 (m, 2H), 2.89 (s, 2H), 1.28 (t, *J*=7.1, 3H), 0.91 (s, 9H), 0.07 (s, 6H). **^{13}C NMR** (176 MHz, $CDCl_3$) δ 169.29, 149.08, 145.99, 130.87, 122.92, 114.75, 113.88, 110.25, 66.83, 66.21, 65.60, 61.36, 56.02, 40.59, 26.08, 14.32, -5.19.

ethyl 2-(4-((2-(((*tert*-butyldimethylsilyl)oxy)methyl)-1,3-dioxolan-2-yl)methyl)-2,6-dimethoxyphenoxy)acetate (**S12**)

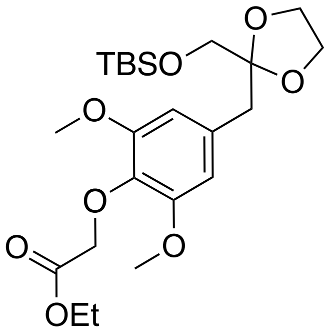

To a stirred solution of **S6** (0.235 g, 1.03 mmol) in toluene (20 mL) is added campher-10-sulfonic acid (0.014 g, 0.05 eqv.) and ethylene glycol (0.29 mL, 5.00 eqv.) heated at reflux for 2 hours. Upon completion, mixture is cooled and quenched with H_2O, washed with brine, dried over $MgSO_4$ and concentrated *in vacuo.* To a solution of crude **S8** (0.201 g, 0.74 mmol) in acetone (20 mL) is added K_2CO_3 (0.123 g, 1.20 eqv) and ethyl bromoacetate (0.1 mL, 1.20 eqv.) and heated to reflux for 1 hour. Upon completion, mixture is filtered through celite and concentrated *in vacuo* to yield crude **S10** as a yellow oil. To a solution of crude **S10** (0.265 g, 0.74 mmol) in DCM (10 mL) is added imidazole (0.102 g, 2.00 eqv.), DMAP (0.005 g, 0.05 eqv.) and TBS-Cl (0.14 g, 1.25 eqv.) and stirred for 16 hours. Upon completion, reaction is quenched with NH_4Cl (saturated solution), washed with H_2O, brine, dried over $MgSO_4$ and concentrated *in vacuo*. Crude **S12** is purified by column chromatography (10-30% ethyl acetate in petroleum ether) to give pure **S12** as a colourless oil (0.206 g, 42% over 3 steps).

IR (FTIR)ν_{max}: 2950, 2354, 1975, 1759, 1593, 1506 cm^{-1}; **HRMS** (NSI+) *m/z* [M + H^+] calcd. for $C_{23}H_{39}O_8Si^+$ 471.2409, found 471.2397; ^{1}**H NMR** (500 MHz, $CDCl_3$) δ = 6.53 (s, 2H), 4.59 (s, 2H), 4.26 (q, *J*=7.1, 2H), 3.90 – 3.84 (m, 2H), 3.82 (s, 6H), 3.67 – 3.62 (m, 2H), 3.50 (s, 2H), 2.88 (s, 2H), 1.30 (t, *J*=7.1, 3H), 0.91 (s, 9H), 0.07 (s, 6H). ^{13}**C NMR** (126 MHz, $CDCl_3$) δ 169.79, 152.19, 134.97, 132.53, 110.23, 107.91, 69.92, 66.24, 65.61, 61.02, 56.24, 41.27, 26.07, 18.52, 14.40, 1.16, -5.22.

ethyl 2-(4-((2-(((*tert*-butyldimethylsilyl)oxy)methyl)-1,3-dioxolan-2-yl)methyl)-2-methoxyphenoxy)-3-(3,4-dimethoxyphenyl)-3-hydroxypropanoate (**S13**)

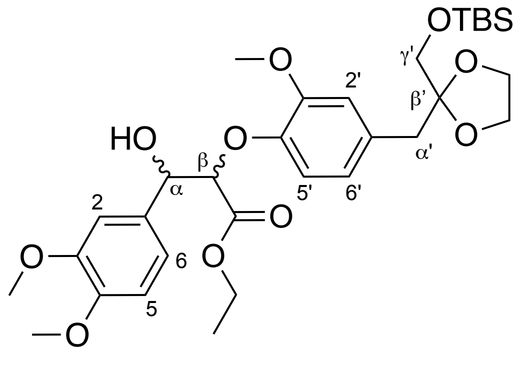

General procedure from Reference [5]. **S11** (0.372 g, 0.84 mmol, 1.00 eqv.), 3,4-dimethoxybenzaldehyde (0.147 g, 0.88 mmol, 1.05 eqv.), THF (5 mL). LDA in THF (6 mL): *n*-BuLi (0.45 mL of 2.30 M, 1.20 eqv.); Diisopropylamine (0.15 mL, 1.30 eqv.). Reaction left at -78 °C for 2 hours. Product obtained as a viscous oil after column chromotgraphy (10-40% ethyl acetate in petroleum ether) to give **S13** (0.209 g, 41%) as a mixture of diastereoisomers (*d.r.* 2.57:1, measured by integration of peak at 5.13 ppm (dd) for major diastereoisomer and peak at 5.04 ppm (dd) for minor diasteroisomer).

IR (FTIR)ν_{max}: 2968, 2358, 1749, 1739, 1683, 1508, 1261 cm^{-1}; **HRMS** (NSI+) *m/z* [M + NH_4^+] calcd. for $C_{31}H_{50}O_{10}Si^+$ 624.3198, found 624.3209; **^{1}H NMR** (700 MHz, $CDCl_3$) δ = 7.05 (d, *J*=2.1, 0.72H, H2 *major*), 6.97 (m, 1H, H6 *major/minor*), 6.95 – 6.74 (m, 5H), 5.13 (dd, *J*=6.2, 4.8, 0.72H, Hα *major*), 5.04 (dd, *J*=7.4, 2.6, 0.28H, Hα *minor*), 4.71 (d, *J*=4.8, 0.72H, Hβ *major*), 4.43 (d, *J*=7.3, 0.28H, Hβ *minor*), 4.13 (q, *J*=7.1, 1.44H, $OC\underline{H}_2CH_3$ *major*), 4.09 – 4.00 (m, 0.56H, $OC\underline{H}_2CH_3$ *minor*), 3.88- 3.85 (m, 11H, 3 x OMe *major/minor*, 1 x O-$C\underline{H}_2C\underline{H}_2$-O *major/minor*), 3.66 – 3.62 (m, 0.56H, 1 x O-$C\underline{H}_2C\underline{H}_2$-O *minor*), 3.64 – 3.59 (m, 1.44H, 1 x O-$C\underline{H}_2C\underline{H}_2$-O major), 3.49 (s, 0.56H, Hγ' *minor*), 3.48 (s, 1.44H, Hγ' *major*), 2.89 (s, 0.56H, Hα' *minor*), 2.89 (s, 1.44H, Hα' *major*), 1.15 (t, *J*=7.1, 2.16H, $OCH_2C\underline{H}_3$ *major*), 1.06 (t, *J*=7.1, 0.84H, $OCH_2C\underline{H}_3$ *minor*), 0.91 (m, 9H, Si-$(CH_3)_2(C\underline{H}_3)_3$m *major/minor*), 0.07 (m, 6H, Si-$(C\underline{H}_3)_2(CH_3)_3$, *major/minor*). **^{13}C NMR** (176 MHz, $CDCl_3$) δ 169.62 ($\underline{C}O_2Et$ *minor*), 169.50 ($\underline{C}O_2Et$ *major)*, 150.05, 149.82, 149.20, 149.05, 148.86, 148.81, 146.03, 145.94, 132.58, 132.55, 131.81, 130.64, 123.46, 123.36, 119.78, 119.40, 118.65, 117.94, 114.88, 114.86, 110.82, 110.79, 110.22, 110.20, 110.08, 86.05 (Cβ *minor*), 84.43 (Cβ *major*), 75.04 (Cα *minor*), 73.92 (Cα *major*), 66.15 (Cγ' *major*), 66.07

($\underline{C}\gamma'$ *minor*), 65.57 (2C, O-$\underline{C}H_2\underline{C}H_2$-O), 61.33 (O$\underline{C}H_2CH_3$ *major*), 61.30 (O$\underline{C}H_2CH_3$ *minor*), 56.08 (OMe), 56.03 (OMe), 56.00 (2C, 2 x OMe), 55.96 (OMe), 55.93 (OMe), 40.65 ($\underline{C}\alpha'$*major/minor*), 26.06 (Si-$(CH_3)_2(\underline{C}H_3)_3$, *major/minor*), 14.24 ($OCH_2\underline{C}H_3$ *major*), 14.09 ($OCH_2\underline{C}H_3$ *minor*), -5.20 (Si-$(\underline{C}H_3)_2(CH_3)_3$, *major/minor*).

ethyl 2-(4-((2-(((*tert*-butyldimethylsilyl)oxy)methyl)-1,3-dioxolan-2-yl)methyl)-2,6-dimethoxyphenoxy)-3-hydroxy-3-(3,4,5-trimethoxyphenyl)propanoate (**S14**)

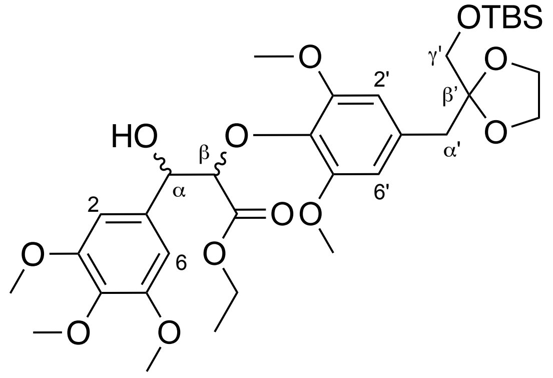

General procedure from Reference [5]. **S12** (0.236 g, 0.50 mmol, 1.00 eqv.), 3,4,5-trimethoxybenzaldehyde (0.101 g, 0.52 mmol, 1.05 eqv.), THF (4 mL). LDA in THF (5 mL): *n*-BuLi (0.26 mL of 2.3 M, 1.20 eqv.); Diisopropylamine (0.09 mL, 1.30 eqv.). Reaction left at -78 °C for 2 hours. Product obtained as a viscous oil after column chromotgraphy (20-40% ethyl acetate in petroleum ether) to give **S13** (0.229 g, 69%) as a mixture of diastereoisomers (*d.r.* 4.1:1, measured by integration of peak at 4.67 ppm (d) for major diastereoisomer and peak at 4.01 ppm (d) for minor diasteroisomer).

IR (FTIR)ν_{max}: 2975, 2358, 1975, 1749, 1734, 1593, 1506, 1458 cm^{-1}; **HRMS** (NSI+) *m/z* [M + NH_4^+] calcd. for $C_{33}H_{50}NO_{12}Si^+$ 684.3410, found 684.3410; **^{1}H NMR** (700 MHz, $CDCl_3$) δ = 6.67 (s, 1.6H, $\underline{H}$2,6 *major*), 6.61 (s, 0.4H, $\underline{H}$2,6 *minor*), 6.58 (s, 1.6H, $\underline{H}$2',6' *major*), 6.56 (s, 0.4H, $\underline{H}$2,6 *minor*), 4.94 – 4.89 (m, 1H, $\underline{H}\alpha$ *major/minor*), 4.71 (d, *J*=7.9, 0.8H, α-O$\underline{H}$ *major*), 4.67 (d, *J*=3.9, 0.8H, $\underline{H}\beta$ major), 4.15 – 3.98 (m, 2H, $OC\underline{H}_2CH_3$ *major/minor*), 4.01 (d, *J*=8.5, 0.2H, $\underline{H}\beta$ minor), 3.89 – 3.79 (m, 17H, 5 x OMe, 1 x O-$C\underline{H}_2C\underline{H}_2$-O *major/minor*), 3.68 – 3.60 (m, 2H, 1 x O-$C\underline{H}_2C\underline{H}_2$-O *major/minor*), 3.51 (m, 2H, $\underline{H}\gamma'$ *major/minor*), 2.95 – 2.86 (m, 2H, $\underline{H}\alpha'$ *major/minor*), 1.07 (t, *J*=7.1, 2.4H, $OCH_2C\underline{H}_3$ *major*), 1.04 (t, *J*=7.1, 0.6H, $OCH_2C\underline{H}_3$ *minor*), 0.92 (s, 9H, Si-$(CH_3)_2(C\underline{H}_3)_3$ *major/minor*), 0.08 (s, 6H, Si-$(C\underline{H}_3)_2(CH_3)_3$, *major/minor*). **^{13}C NMR** (176 MHz, $CDCl_3$) δ 169.67 ($\underline{C}O_2Et$ *minor)*, 168.90 ($\underline{C}O_2Et$ *major)*, 153.52, 153.25, 153.07, 151.95,

151.68, 137.59, 136.73, 135.79, 134.80, 134.68, 133.40, 133.32, 133.24, 110.19 (Cβ'), 107.90 (2C, S2',6' *major*), 107.69 (2C, S2',6' *minor*), 103.92 (C2,6 *major*), 103.89 (C2,6 *minor*), 89.88 (Cβ *minor*), 85.95 (Cβ *major*), 75.98 (Cα *minor*), 73.93 (Cα *major*), 66.25 (Cγ' *major*), 66.07 (Cγ' *minor*), 65.62 (2C, O-CH_2CH_2-O) , 61.01 (*p*-OMe *minor*), 60.94 (*p*-OMe *major*) , 60.87 (OCH_2CH_3 *minor*), 60.69 (OCH_2CH_3 *major*), 56.26, 56.23, 56.15, 41.34 (Cα' *major/minor*), 26.07 (Si-$(CH_3)_2(CH_3)_3$, *major/minor*), 14.23 (OCH_2CH_3 *major*), 14.20 (OCH_2CH_3 *minor)*, -5.17 (Si-$(CH_3)_2(CH_3)_3$, *major/minor*).

2-(4-((2-(((*tert*-butyldimethylsilyl)oxy)methyl)-1,3-dioxolan-2-yl)methyl)-2-methoxyphenoxy)-1-(3,4-dimethoxyphenyl)propane-1,3-diol (**S15**)

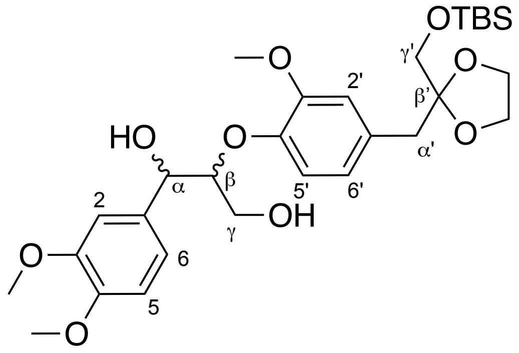

To a stirred solution of **S13** (0.196 g, 0.32 mmol, 1.00 eqv) in EtOH (5 mL) is added $NaBH_4$ (0.061 g, 5.00 eqv.) followed by the slow addition of MeOH (0.2 mL, 15.00 eqv.) and stirred for 16 hours. Upon completion, mixture is quenched with NH_4Cl (saturated solution), extracted with ethyl acetate (3 x), washed with water, brine, dried and concentrated *in vacuo* to give pure **S15** (0.117 g, 64%), as a colourless oil, with no need for purification and as a mixture of diastereoisomers (*d.r.* 3:1 measured by integration of peak at 4.13 ppm (ddd) for major diastereoisomer and peak at 3.98 ppm (dt) for minor diasteroisomer).

IR (FTIR)ν_{max}: 2985, 2900, 2358, 1558, 1516, 1456 cm^{-1}; **HRMS** (NSI+) *m/z* [M + NH_4^+] calcd. for $C_{29}H_{48}NO_9Si^+$ 582.3098, found 582.3104; **^{1}H NMR** (700 MHz, $CDCl_3$) δ = 7.03 – 6.82 (m, 6H), 4.99 – 4.96 (m, 1H, Hα *major/minor*), 4.13 (ddd, *J*=8.0, 5.1, 3.5, 0.75H, Hβ *major*), 3.98 (dt, *J*=7.9, 3.4, 0.25H, Hβ *minor*), 3.93 – 3.87 (m, 3H, O-CH_2CH_2-O, 1 x Hγ *major*), 3.89 – 3.86 (m, 10H, 3 x OMe, 1 x Hγ *minor*), 3.66 (m, 2H, O-CH_2CH_2-O), 3.62 (ddd, *J*=11.9, 7.9, 3.5, 0.75H, 1 x Hγ *major*), 3.51 – 3.49 (m, 2H, Hγ' *major/minor*), 3.45 (ddd, *J*=12.3, 8.6, 3.7, 0.25H, Hγ *minor*),

2.92 (m, 2H, H̲α' *major/minor*), 2.76 – 2.72 (m, 0.25H, γ-OH̲ minor), 2.72 (dd, *J*=8.0, 5.3, 0.75H, γ-OH̲ *major*), 0.92 (s, 9H, Si-$(CH_3)_2(C\underline{H}_3)_3$, *major/minor*), 0.11 – 0.03 (m, 6H, Si-$(C\underline{H}_3)_2(CH_3)_3$, *major/minor*). **^{13}C NMR** (176 MHz, $CDCl_3$) δ 151.13, 150.79, 149.20, 149.15, 149.01, 148.55, 146.07, 145.33, 133.02, 132.42, 132.19, 124.09, 124.05, 120.70, 119.79, 118.42, 114.77, 111.11, 110.23 (C̲β'), 109.96, 109.18, 89.83 (C̲β *minor*), 87.75 (C̲β *major*), 74.05 (C̲α *minor),* 72.70 (C̲α *major*), 66.03 (C̲γ' *major/minor*), 65.58 (2C, O-$\underline{C}H_2\underline{C}H_2$-O) , 61.08 (C̲γ *minor*), 60.80 (C̲γ *major*), 56.06 (OMe), 40.71 (C̲α' *major*), 40.68 (C̲α' *minor*), 26.07 (Si-$(CH_3)_2(\underline{C}H_3)_3$, *major/minor*), -5.18 (Si-$(\underline{C}H_3)_2(CH_3)_3$, *major/minor*).

2-(4-((2-(((*tert*-butyldimethylsilyl)oxy)methyl)-1,3-dioxolan-2-yl)methyl)-2,6-dimethoxyphenoxy)-1-(3,4,5-trimethoxyphenyl)propane-1,3-diol (**S16**)

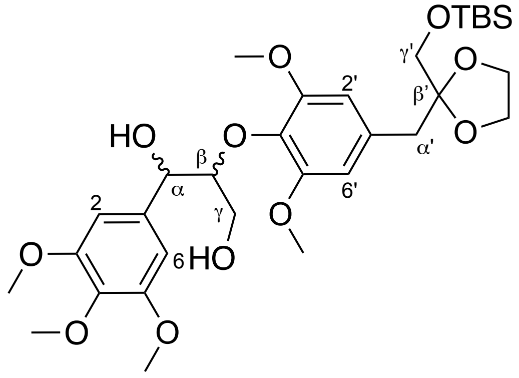

To a stirred solution of **S14** (0.212 g, 0.32 mmol, 1.00 eqv) in EtOH (5 mL) is added $NaBH_4$ (0.060 g, 5.00 eqv.) followed by the slow addition of MeOH (0.19 mL, 15.00 eqv.) and stirred for 16 hours. Upon completion, mixture is quenched with NH_4Cl (saturated solution), extracted with ethyl acetate (3 x), washed with water, brine, dried and concentrated *in vacuo* to give pure **S16** (0.139 g, 70%), as a colourless oil, with no need for purification and as a mixture of diastereoisomers (*d.r.* 5.6:1 measured by integration of peak at 5.00 ppm (t) for major diastereoisomer and peak at 5.02 ppm (d) for minor diasteroisomer).

IR (FTIR)ν_{max}: 2968, 2885, 2355, 1558, 1521, 1456 cm^{-1}; **HRMS** (NSI+) *m/z* [M + NH_4^+] calcd. for $C_{31}H_{52}NO_{11}Si^+$ 642.3304, found 642.3299; **^{1}H NMR** (700 MHz, $CDCl_3$) δ = 6.70 (s, 0.3H, H̲2,6 *minor*), 6.63 (s, 1.7H, H̲2',6' *major*), 6.61 (s, 0.3H *minor,* H̲2',6' *minor*), 6.57 (s, 1.7H, H̲2,6 *major*), 5.02 (d, *J*=8.8, 0.15H, H̲α *minor*), 5.00 (t, *J*=3.4, 0.85H, H̲α *major*), 4.18 (d, *J*=3.1, 0.85H, α-OH̲ *major*), 4.14 – 4.10 (m, 1H, H̲β *major*), 3.91 (m, 2H, O-$C\underline{H}_2C\underline{H}_2$-O major/minor), 3.89 (m,

1H, 1 x Hγ *major*), 3.87 (s, 5.1H, 2 x OMe *major*), 3.87 (s, 0.9H 2 x OMe *minor*), 3.85 (s, 5.1H, 2 x OMe *major*), 3.83 (s, 0.45H, *p*-OMe *minor*), 3.82 (s, 2.55H, *p*-OMe *major*), 3.75 – 3.70 (m, 2H, O-CH_2CH_2-O *major/minor*), 3.70 – 3.67 (m, 0.3H, O-CH_2CH_2-O *minor*), 3.58 (dt, *J*=12.5, 3.4, 0.15H, 1 x Hγ *minor*), 3.52 (s, 1.7H, Hγ' *major*), 3.51 (s, 0.3H, Hγ' *minor*), 3.46 (ddd, *J*=11.6, 8.4, 2.7, 1H, 1 x Hγ *major*), 3.41 (dd, *J*=9.8, 3.8, 0.15H, γ'-OH minor), 3.34 (ddd, *J*=12.4, 9.8, 2.5, 0.15H, 1 x Hγ *minor*), 3.18 (dd, *J*=8.5, 3.6, 1H, γ-OH *major*), 2.97 – 2.91 (m, 2H, Hα' *major/minor*), 0.93 (s, 9H, Si-$(CH_3)_2(CH_3)_3$, *major/minor*), 0.08 (s, 6H, Si-$(CH_3)_2(CH_3)_3$, *major/minor*). **^{13}C NMR** (176 MHz, $CDCl_3$) δ 153.39 (2C, C3/C5), 153.32 (2C, C3/C5), 152.80 (C4), 152.44 (C4), 137.18, 135.13, 133.45, 133.39, 110.25 (Cβ'), 107.95 (C2'/C6' *major*), 107.85 (C2'/C6' *major*), 104.34 (C2/C6 *minor*), 102.80 (C2/C6 *major*), 89.00 (Cβ *minor*), 87.15 (Cβ *major*), 74.55 (Cα *minor*), 72.78 (Cα *major*), 66.03 (Cγ' *minor*), 65.89 (Cγ' *major*), 65.60 (O-CH_2CH_2-O *major/minor*), 61.01 (*p*-OMe *major*), 60.98 (*p*-OMe *minor)*, 60.76 (Cγ *major*), 60.67 (Cγ *minor*), 56.29 (*o*-OMe *major/minor*), 41.33 (Cα' major), 41.28 (Cα' minor), 26.07 (Si-$(CH_3)_2(CH_3)_3$, *major/minor*), -5.16 (Si-$(CH_3)_2(CH_3)_3$, *major/minor*).

2-(4-((2-(((*tert*-butyldimethylsilyl)oxy)methyl)-1,3-dioxolan-2-yl)methyl)-2-methoxyphenoxy)-1-(3,4-dimethoxyphenyl)-3-hydroxypropan-1-one (**S17**)

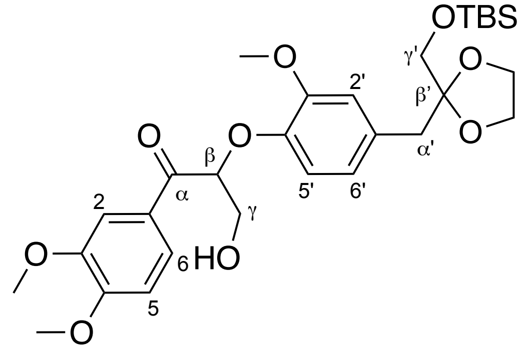

To a stirred solution of **S15** (0.102 g, 0.18 mmol, 1.00 eqv.) in DCM (5 mL) is added DDQ (0.049 g, 1.20 eqv.) and stirred for 16 hours. Upon completion, mixture is quenched with $NaHCO_3$ (saturated solution), extracted with DCM (3x), washed with $NaHCO_3$ (sat. solution), brine, dried over Na_2SO_4, concentrated *in vacuo* to give **S17** (0.101 g, 100%) as a colourless oil.

IR (FTIR)ν_{max}: 2972, 2357, 1683, 1558, 1508, 1456 cm^{-1}; **HRMS** (NSI+) *m/z* [M + H^+] calcd. for $C_{29}H_{43}O_9Si^+$ 563.2676, found 563.2674; **^{1}H NMR** (700 MHz, Chloroform-*d*) δ = 7.74 (dd, *J*=8.5,

2.0, 1H, H6), 7.60 (d, *J*=2.0, 1H, H2), 6.90 – 6.87 (m, 2H, H2',H5), 6.79 (d, *J*=8.2, 1H, H5'), 6.74 (dd, *J*=8.2, 1.9, 1H, H6'), 5.38 (t, *J*=5.2, 1H, Hβ), 4.06 (d, *J*=5.2, 2H, Hγ), 3.95 (s, 3H, OMe), 3.92 (s, 3H OMe), 3.86 – 3.83 (m, 5H, 1 x OMe, 1 x O-C$\underline{H}_2$C$\underline{H}_2$-O), 3.64 – 3.58 (m, 2H, 1 x O-C$\underline{H}_2$C$\underline{H}_2$-O), 3.48 (s, 2H, Hγ'), 2.88 (s, 2H, Hα'), 0.91 (s, 9H, Si-$(CH_3)_2$(C$\underline{H}_3)_3$), 0.06 (s, 6H, Si-(C$\underline{H}_3)_2$$(CH_3)_3$). **^{13}C NMR** (176 MHz, $CDCl_3$) δ 195.24 (Cα), 154.04, 149.95, 149.31, 145.51, 132.28, 128.17, 123.80, 123.48, 118.11, 114.91, 111.04, 110.18, 84.74 (Cβ), 66.12 (Cγ'), 65.57 (O-$\underline{C}$$H_2$$\underline{C}$$H_2$-O), 63.78 ($\underline{C}$γ), 56.28 (OMe), 56.14 (OMe), 55.97 (OMe), 40.63 ($\underline{C}$α'), 26.06 (Si-$(CH_3)_2$($\underline{C}$$H_3)_3$), -5.19 (Si-($\underline{C}$$H_3)_2$$(CH_3)_3$).

2-(4-((2-(((*tert*-butyldimethylsilyl)oxy)methyl)-1,3-dioxolan-2-yl)methyl)-2,6-dimethoxyphenoxy)-3-hydroxy-1-(3,4,5-trimethoxyphenyl)propan-1-one (**S18**)

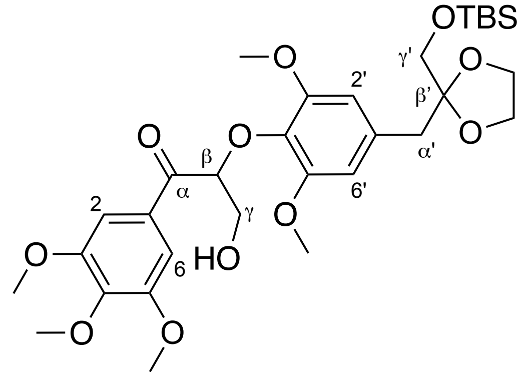

To a stirred solution of **S16** (0.131 g, 0.21 mmol, 1.00 eqv.) in DCM (5 mL) is added DDQ (0.050 g, 1.05 eqv.) and stirred for 16 hours. Upon completion, mixture is quenched with $NaHCO_3$ (saturated solution), extracted with DCM (3x), washed with $NaHCO_3$ (sat. solution), brine, dried over Na_2SO_4, concentrated *in vacuo* to give **S17** (0.110 g, 84%) as a colourless oil.

IR (FTIR)ν_{max}: 2987, 2900, 2358, 1687, 1558, 1506, 1456 cm^{-1}; **HRMS** (NSI+) *m/z* [M + H^+] calcd. for $C_{31}H_{47}O_{11}Si^+$ 623.2888, found 623.3893; **^{1}H NMR** (700 MHz, $CDCl_3$) δ = 7.38 (s, 2H, $\underline{H}$2, 6), 6.56 (s, 2H, $\underline{H}$2',6'), 5.08 (dd, *J*=7.2, 3.1, 1H, $\underline{H}$β), 4.00 (dd, *J*=12.3, 7.3, 1H, $\underline{H}$γ), 3.92 (s, 3H, OMe), 3.90 (s, 6H, 2 x OMe), 3.87 (m, 2H, O-C$\underline{H}_2$C$\underline{H}_2$-O), 3.85 – 3.80 (m, 1H, $\underline{H}$γ), 3.73 (s, 6H, 2 x OMe), 3.70 – 3.61 (m, 2H, O-C$\underline{H}_2$C$\underline{H}_2$-O), 3.50 (s, 2H, $\underline{H}$γ'), 2.94 – 2.85 (m, 2H, $\underline{H}$α'), 0.92 (s, 9H, Si-$(CH_3)_2$(C$\underline{H}_3)_3$), 0.07 (s, 6H, Si-(C$\underline{H}_3)_2$$(CH_3)_3$). **^{13}C NMR** (176 MHz, $CDCl_3$) δ 195.68 ($\underline{C}$α), 153.12, 152.00, 142.82, 134.84, 133.20, 130.92, 110.19, 107.88, 106.54, 87.41 ($\underline{C}$β), 66.10

(C̲γ'), 65.59 (O-C̲H$_2$C̲H$_2$-O), 63.44 (C̲γ), 61.10 (*p*-OMe), 56.47 (2C, 2 x OMe), 56.11 (2C, 2 x OMe), 41.28 (C̲α'), 26.06 (Si-(CH$_3$)$_2$(C̲H$_3$)$_3$), -5.18 (Si-(C̲H$_3$)$_2$(CH$_3$)$_3$).

1-(3,4-dimethoxyphenyl)-3-hydroxy-2-(4-(3-hydroxy-2-oxopropyl)-2-methoxyphenoxy)propan-1-one (**3**)

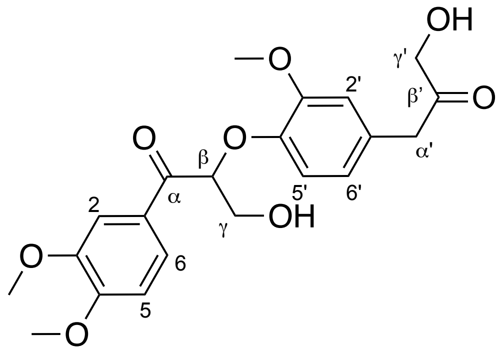

To a stirred solution of **S17** (0.101 g, 0.18 mmol) in acetone (5 mL) is added I_2 (0.009 g, 0.20 eqv.) and stirred for 16 hours. Upon completion, mixture is quenched with $Na_2S_2O_3$ (sat. solution), extracted with ethyl acetate (3x), washed with $Na_2S_2O_3$ (sat. solution), brine, dried over Na_2SO_4 and concentrated *in vacuo*. Product obtained as a viscous oil after column chromotgraphy (0-5% MeOH in DCM) to give **2** (0.068 g, 94%).

IR (FTIR)ν_{max}: 2985, 2900, 1734, 1716, 1683, 1593, 1508 cm^{-1}; **HRMS** (NSI+) *m/z* [M + H$^+$] calcd. for $C_{21}H_{25}O_8^+$ 405.1549, found 405.1550; **^{1}H NMR** (700 MHz, $CDCl_3$) δ = 7.73 (dd, *J*=8.4, 2.0, 1H, H6), 7.60 (d, *J*=2.0, 1H, H2), 6.89 (d, *J*=8.4, 1H, H̲5), 6.84 (d, *J*=8.1, 1H, H̲5'), 6.75 (d, *J*=2.0, 1H, H̲2'), 6.66 (dd, *J*=8.1, 2.0, 1H, H̲6'), 5.40 (dd, *J*=6.0, 4.6, 1H, H̲β), 4.28 (d, *J*=4.7, 2H, H̲γ'), 4.07 (dd, *J*=7.5, 4.5, 2H, H̲γ), 3.95 (s, 3H, OMe), 3.92 (s, 3H, OMe), 3.85 (s, 3H, OMe), 3.65 (s, 2H, H̲α'), 2.97 (t, *J*=4.8, 1H, γ'-OH̲), 2.90 (dd, *J*=7.5, 5.8, 1H, γ-OH̲). **^{13}C NMR** (176 MHz, $CDCl_3$) δ 207.35 (Cβ'), 194.83 (Cα), 154.15, 150.78, 149.39, 146.48, 128.10, 128.04, 123.74, 121.98, 118.69, 113.28, 110.98, 110.23, 84.51 (Cβ), 67.74 (C̲γ'), 63.89 (C̲γ), 56.30 (OMe), 56.15 (OMe), 56.03 (OMe), 45.47 (Cα').

^{1}H NMR (700 MHz, DMSO-*d*$_6$) δ = 7.80 (dd, *J*=8.5, 2.0, 1H, H̲6), 7.52 (d, *J*=2.0, 1H, H̲2), 7.08 (d, *J*=8.5, 1H, H̲5), 6.80 (d, *J*=1.9, 1H, H̲2'), 6.67 (d, *J*=8.2, 1H, H̲5'), 6.58 (dd, *J*=8.2, 1.9, 1H, H̲6'), 5.60 (t, *J*=5.0, 1H, H̲β), 5.18 (t, *J*=5.8, 1H, γ'-OH̲), 5.12 (t, *J*=5.9, 1H, γ-OH̲), 4.12 (d, *J*=5.8, 2H, H̲γ'), 3.87 (t, *J*=6.0, 2H, H̲γ), 3.85 (s, 3H, OMe), 3.79 (s, 3H, OMe), 3.72 (s, 3H, OMe), 3.61 (s,

2H, Hα'). **^{13}C NMR** (176 MHz, DMSO-d_6) δ 208.44 (Cβ'), 195.21 (Cα), 153.46, 148.85, 148.57, 145.63, 127.90, 127.81, 123.33 (C6), 121.49 (C6'), 114.30 (C5'), 114.05 (C2'), 110.93 (C5), 110.68 (C2), 81.35 (Cβ), 67.19 (Cγ'), 62.54 (Cγ), 55.80 (OMe), 55.52 (OMe), 55.45 (OMe), 44.02 (Cα').

3-hydroxy-2-(4-(3-hydroxy-2-oxopropyl)-2,6-dimethoxyphenoxy)-1-(3,4,5-trimethoxyphenyl)propan-1-one (**4**)

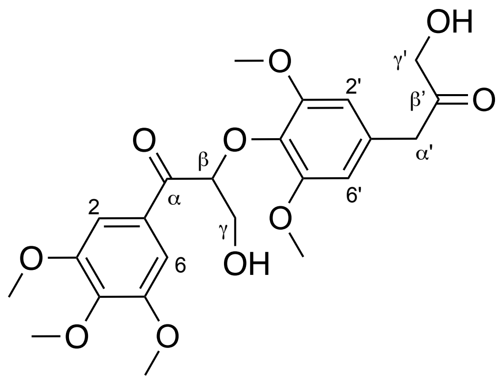

To a stirred solution of **S18** (0.101 g, 0.16 mmol) in acetone (5 mL) is added I_2 (0.008 g, 0.20 eqv.) and stirred for 16 hours. Upon completion, mixture is quenched with $Na_2S_2O_3$ (sat. solution), extracted with ethyl acetate (3x), washed with $Na_2S_2O_3$ (sat. solution), brine, dried over Na_2SO_4 and concentrated *in vacuo*. Product obtained as a viscous oil after column chromotgraphy (0-5% MeOH in DCM) to give **3** (0.037 g, 49%).

IR (FTIR)ν_{max}: 2965, 2904, 1737, 1720, 1683, 1583, 1504 cm^{-1}; **HRMS** (NSI+) *m/z* [M + H$^+$] calcd. for $C_{23}H_{29}O_{10}^+$ 465.1761, found 465.1762; **^{1}H NMR** (700 MHz, $CDCl_3$) δ = 7.35 (s, 2H, H2,6), 6.43 (s, 2H, H2',6'), 5.08 (dd, *J*=7.4, 2.7, 1H, Hβ), 4.32 (d, *J*=4.8, 2H, Hγ'), 3.99 (ddd, *J*=10.3, 7.5, 3.1, 1H, 1 x Hγ), 3.93 (s, 3H, OMe), 3.90 (s, 6H, 2 x OMe), 3.86 – 3.80 (m, 1H, 1 x Hγ), 3.74 (s, 6H, 2 x OMe), 3.67 (s, 2H, Hα'), 2.98 (t, *J*=4.8, 1H, γ'-OH). **^{13}C NMR** (176 MHz, $CDCl_3$) δ 207.02 (Cβ'), 195.27 (Cα), 153.16 (2C, C3, C5), 152.85 (2C, C3',5'), 142.92 (C4), 135.78 (C4'), 130.73 (C1), 129.20 (C1'), 106.45 (4C, C2, C6 & C2', C6'), 87.44 (Cβ), 67.82 (Cγ'), 63.52 (Cγ), 61.11 (OMe), 56.48 (2C, OMe), 56.22 (2C, OMe), 45.98 (Cα').

^{1}H NMR (700 MHz, DMSO-d_6) δ = 7.31 (s, 2H, H2, 6), 6.51 (s, 2H, H2', 6'), 5.23 (dd, *J*=6.6, 5.2, 1H, Hβ), 5.15 (s, 1H, OH), 4.72 (s, 1H, OH), 4.16 (s, 2H, Hγ'), 3.78 (m, 11H, 2 x Hγ, 3 x OMe), 3.66 (s, 2H, Hα'), 3.62 (s, 6H, 2 x OMe). **^{13}C NMR** (176 MHz, DMSO-d_6) δ 208.20 (Cβ'), 195.75

(C̲α), 152.58, 151.99, 141.81, 134.44, 131.21, 130.26, 106.90 (C2',6'), 106.17 (C2, 6), 83.36 (Cβ'), 67.26 (C̲γ'), 62.07 (C̲γ), 60.18 (OMe), 56.02 (2 x OMe), 55.83 (2 x OMe), 44.71 (C̲α').

Comparison of Lignin$^{\alpha\text{-OX}}$ Preparation Procedures (Manuscript Table 1)

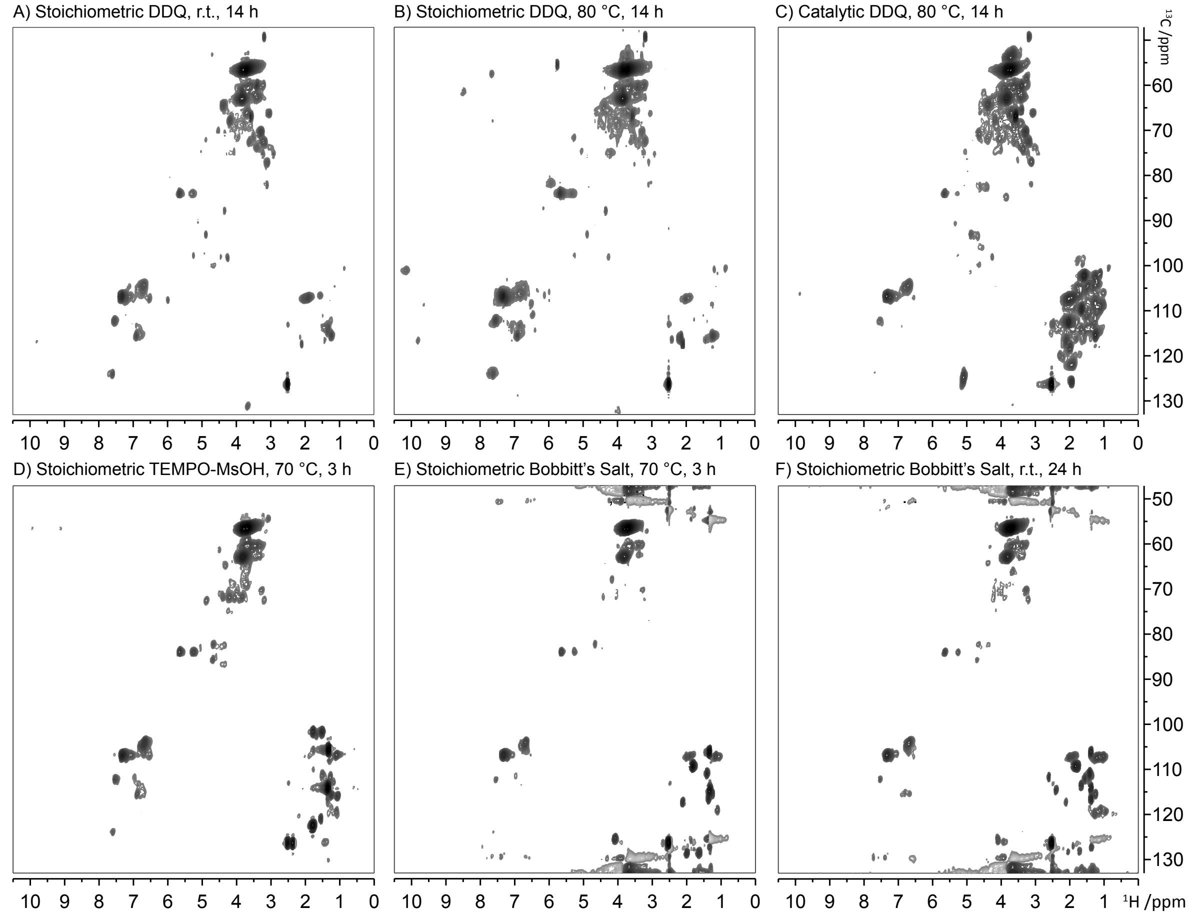

Figure S18: 2D HSQC NMR analysis (700 MHz, d_6-DMSO) of beech lignin$^{\alpha\text{-OX}}$ from reactions of beech dioxasolv lignin (A-F) using different oxidation conditions (see Manuscript Table 1). For assignment of peaks see Figure S6.

Table S6: Ratio values for units **A**, **A'**, **A"** (normalised to 1 for **A**), and **C**, **D**, **E** (normalised to 1 for **C**). Values calculated from using semi-quantitative 2D HSQC NMR on data observed in Figure S18.

**Method**		**A**	**A'**	**A"**	**C**	**D**	**E**
DDQ Stoichiometric, r.t	**S**		1.05	1.66			
14 hours	**G**	1	0.3	0.53	**1.0**	**0.6**	**0.4**
	Total		**1.35**	**2.19**			
DDQ Stoichiometric 80 °C	**S**		1.33	4.17			
2 hours	**G**	1	0.44	1.29	**1.0**	**0.1**	**2.5**
	Total		**1.77**	**5.46**			
Cat. DDQ 80 °C	**S**		0.36	0.84			
14 hours	**G**	1	0.27	0.38	**1.0**	**0.1**	**0.2**
	Total		**0.63**	**1.22**			
TEMPO-MsOH 70 °C	**S**	1	1.02	1.29	**1.0**	**0.0**	**0.0**
3 hours	**G**		0.23	0.26			

	Total		**1.25**	**1.55**			
Bobbitt's Salt. 70 °C	**S**		0.83	1.53			
3 hours	**G**	1	0.36	0.59	**1.0**	**0.1**	**0.1**
	Total		**1.19**	**2.12**			
B.S. r.t	**S**		1.11	1.92			
24 hours	**G**	1	0.27	0.51	**1.0**	**0.2**	**0.1**
	Total		**1.38**	**2.43**			

Scope Study on other Hardwood Lignins (Manuscript Figure 7)

Oak, Maple, Hickory, Cherry and Birch lignins were all extracted using a dioxasolv procedure (General procedures). For 2D HSQC NMR spectra and data from Beech oxidation, See Figure S6 and Table S3.

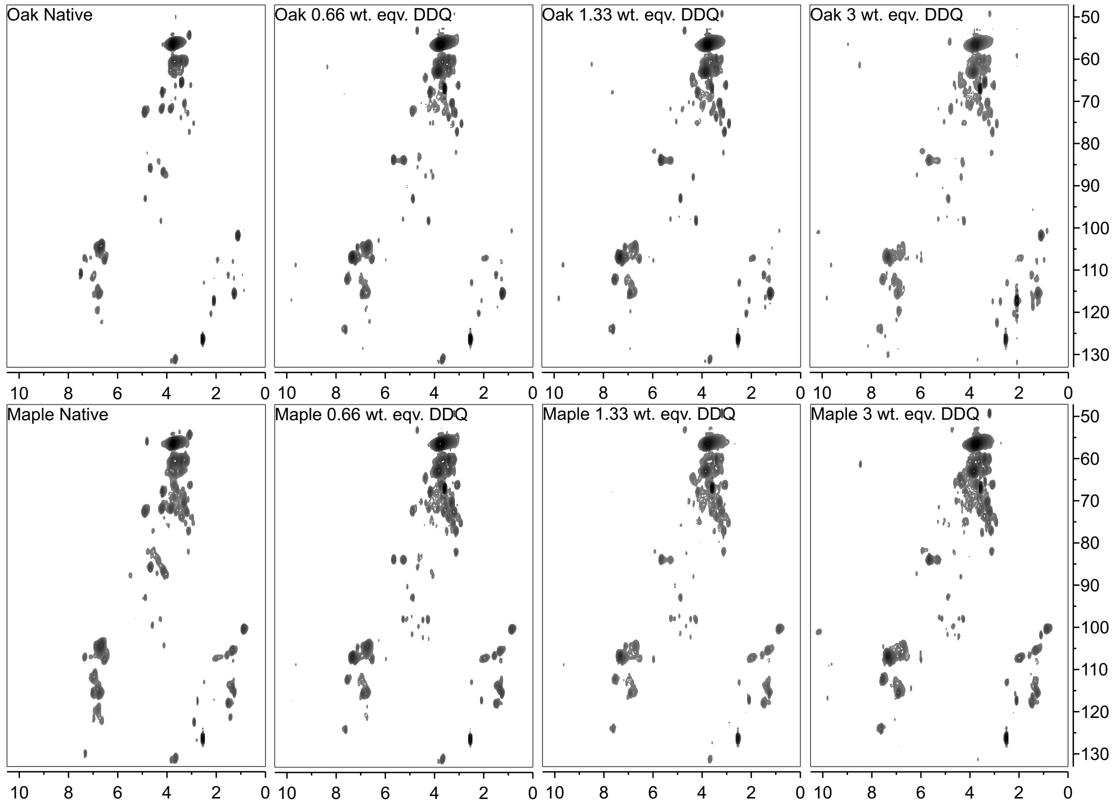

Figure S19: 2D HSQC NMR analysis (700 MHz, d_6-DMSO) of Oak Lignin and Oak Lignin $^{\alpha\text{-OX}}$; Maple Lignin and Maple Lignin$^{\alpha\text{-OX}}$

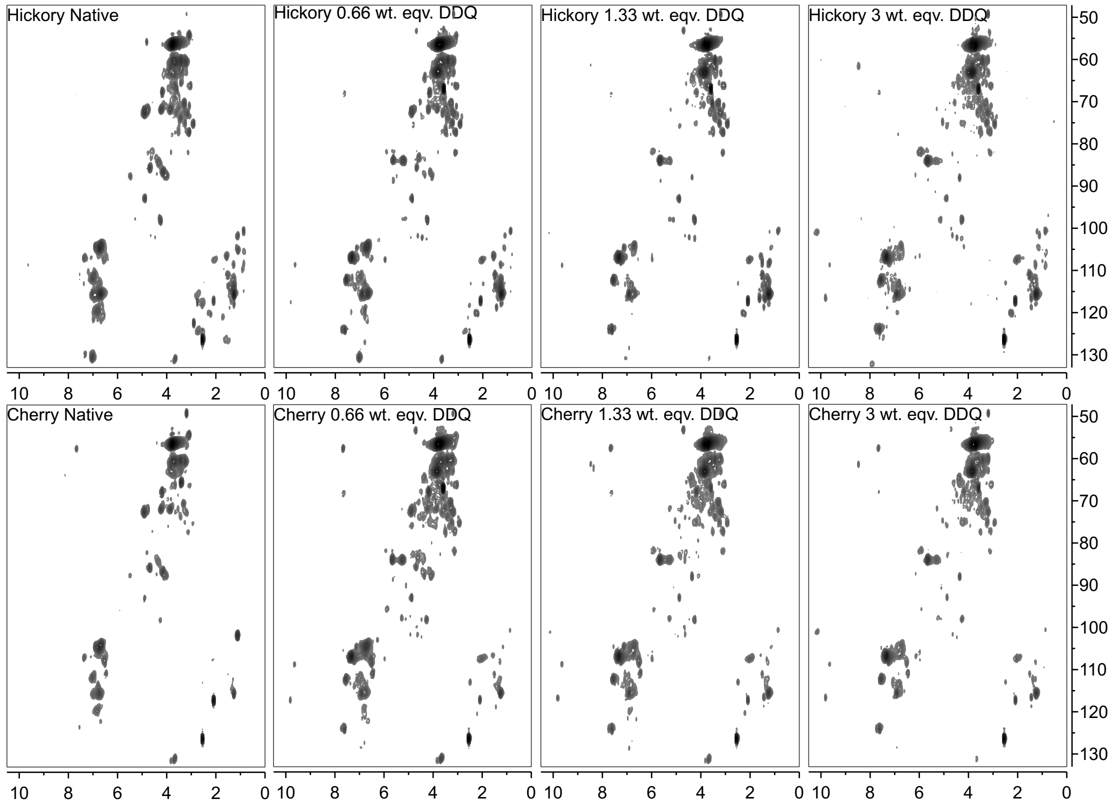

Figure S20: 2D HSQC NMR analysis (700 MHz, d_6-DMSO) of Hickory Lignin and Hickory Lignin $^{\alpha\text{-OX}}$; Cherry Lignin and Cherry Lignin$^{\alpha\text{-OX}}$

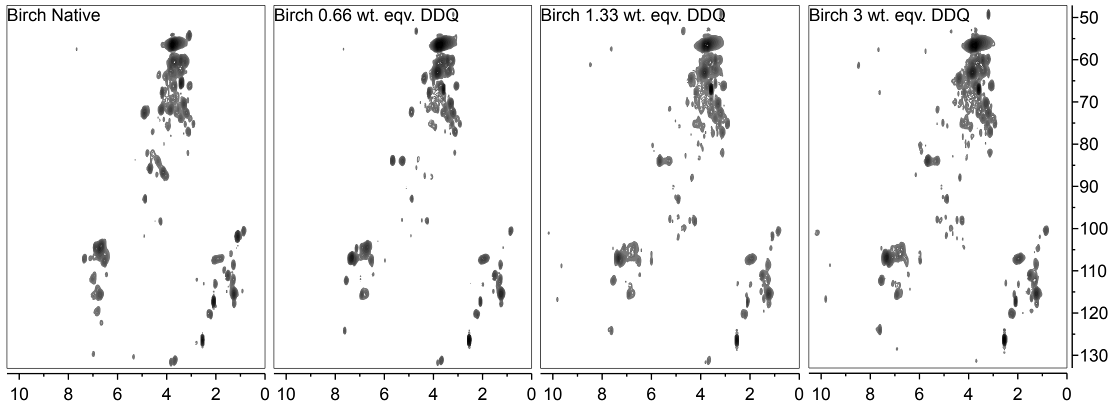

Figure S21: 2D HSQC NMR analysis (700 MHz, d_6-DMSO) of Birch Lignin and Birch Lignin $^{\alpha\text{-OX}}$

Table S7: Integral data from 2D HSQC NMR analysis of hardwood lignin DDQ oxidations (scope study on different lignins with varying weight equivalents of DDQ). In the first grouping of values ($S_{2,6}$, $S_{2,6}^{OX}$, G_2 (integral set arbitrarily to 100 units), G_2^{OX}), a value is obtained for the total aromatic integral obtained from 2D HSQC NMR analysis using the integral regions described below Figure S5. The second grouping (β-O-4 native, β-O-4^{OX} A(S)’, β-O-4^{OX} A(S)’’, β-β resinol native, β-β derived alcohol, β-β derived aldehyde, LBHK, β-O-4^{OX} A(G)’ and β-O-4^{OX} A(G)’’) is reported as raw integrals and a *per* 100 C9 value obtained from using Equation 1 as is the norm.

	**Beech**				**Oak**				**Maple**			
	**Native**	**0.66**	**1.33**	**3**	**Native**	**0.66**	**1.33**	**3**	**Native**	**0.66**	**1.33**	**3**
**$S_{2,6}$**	362.8	310.9	263.5	246.6	335.4	286.13	178.33	107.49	301.9	258.9	206.1	198.1
**$S_{2,6}^{OX}$**	22.0	294.3	299.8	534.9	33.4	254.0	343.7	266.1	24.3	189.6	321.3	414.9
**G_2**	100.0	100.0	100.0	100.0	100.0	100.0	100.0	100.0	100.0	100.0	100.0	100.0
**G_2^{OX}**	12.3	106.5	111.9	173.9	53.8	121.6	137.0	102.1	13.7	78.7	132.0	160.8
**Aromatics**	497.1	811.7	775.2	1055.4	522.5	761.6	759.0	575.7	439.9	627.2	759.4	873.8
**β-O-4 native**												
Raw integral					167.9	123.2	63.6	71.5	133.6	98.1	57.0	59.8
per 100 C9	**34.2**	**12**	**14.8**	**6.2**	**32.1**	**16.2**	**8.4**	**12.4**	**30.4**	**15.6**	**7.5**	**6.8**
**β-O-4^{OX}’ A(S)’**												
Raw integral					10.8	94.7	71.3	41.5	8.6	72.9	74.1	75.0
per 100 C9	**2.3**	**13.1**	**11.9**	**9.6**	**2.1**	**12.4**	**9.4**	**7.2**	**2.0**	**11.6**	**9.8**	**8.6**
**β-O-4^{OX} A(S)”**												
Raw integral					2.1	96.8	167.6	131.0	1.4	54.5	124.0	177.5
per 100 C9	**0.4**	**13.5**	**15.6**	**23.8**	**0.4**	**12.7**	**22.1**	**22.8**	**0.3**	**8.7**	**16.3**	**20.3**
**β-β native**												
Raw integral					37.0	22.4	12.6	11.2	38.4	23.9	17.1	17.6
per 100 C9	**9.4**	**3.7**	**2.5**	**1.6**	**7.1**	**2.9**	**1.7**	**1.9**	**8.7**	**3.8**	**2.2**	**2.0**

**β-β derived alcohol**												
Raw integral					0.0	14.4	14.7	2.5	0.0	10.9	14.6	10.1
per 100 C9	**0**	**4.1**	**2.4**	**1.2**	**0.0**	**1.9**	**1.9**	**0.4**	**0.0**	**1.7**	**1.9**	**1.2**
**β-β derived aldehyde**												
Raw integral					2.3	4.5	16.7	24.1	0.0	5.2	18.6	37.0
per 100 C9	**0**	**0.3**	**1.1**	**2.7**	**0.4**	**0.6**	**2.2**	**4.2**	**0.0**	**0.8**	**2.4**	**4.2**
**LBHK**												
Raw integral					49.3	69.7	52.4	27.3	41.6	55.4	49.6	44.6
per 100 C9	**8.4**	**6.7**	**9.2**	**5.9**	**9.4**	**9.1**	**6.9**	**4.7**	**9.5**	**8.8**	**6.5**	**5.1**
**β-O-4^{OX} A(G)'**												
Raw integral					3.5	22.0	19.1	15.1	3.1	17.0	21.5	22.4
per 100 C9	**0.9**	**3.2**	**3.4**	**2.5**	**0.7**	**2.9**	**2.5**	**2.6**	**0.7**	**2.7**	**2.8**	**2.6**
**β-O-4^{OX} A(G)''**												
Raw integral					3.7	26.1	40.9	34.6	0.0	18.7	33.8	55.6
per 100 C9	**0.8**	**4.3**	**5**	**7.4**	**0.7**	**3.4**	**5.4**	**6.0**	**0.0**	**3.0**	**4.5**	**6.4**
β-5												
					19.9	17.8	9.5	4.8	17.8	12.9	12.2	7.7
per 100 C9	**3.5**	**1**	**6.7**	**0.9**	**3.8**	**2.3**	**1.3**	**0.8**	**4.0**	**2.1**	**1.6**	**0.9**

	**Hickory**				**Cherry**				**Birch**			
	**Native**	**0.66**	**1.33**	**3**	**Native**	**0.66**	**1.33**	**3**	**Native**	**0.66**	**1.33**	**3.00**
**$S_{2,6}$**	178.7	201.1	140.5	136.8	318.0	314.2	218.4	225.6	522.3	460.0	328.8	287.5
**$S_{2,6}^{OX}$**	15.9	145.5	340.3	427.3	23.7	237.6	380.5	525.1	38.2	413.4	616.5	698.8

**G_2**	100.0	100.0	100.0	100.0	100.0	100.0	100.0	100.0	100.0	100.0	100.0	100.0
**G_2^{OX}**	16.8	121.9	209.1	254.3	15.2	100.2	134.9	190.2	12.8	109.2	143.8	154.7
**Aromatics**	311.4	568.5	789.9	918.4	456.9	752.0	833.8	1040.9	673.3	1082.6	1189.1	1241.0
**β-O-4 native**												
Raw integral	137.7	119.6	70.6	76.4	176.7	138.6	61.5	77.4	239.5	166.7	76.4	67.8
per 100 C9	**44.2**	**21.0**	**8.9**	**8.3**	**38.7**	**18.4**	**7.4**	**7.4**	**35.6**	**15.4**	**6.4**	**5.5**
**β-O-4^{OX} A(S)’**												
Raw integral	6.9	84.9	79.4	74.6	11.0	105.9	93.4	105.9	13.3	145.6	128.7	120.1
per 100 C9	**2.2**	**14.9**	**10.0**	**8.1**	**2.4**	**14.1**	**11.2**	**10.2**	**2.0**	**13.4**	**10.8**	**9.7**
**β-O-4^{OX} A(S)”**												
Raw integral	2.4	65.8	206.0	283.9	2.1	95.2	191.1	273.1	1.8	137.9	259.3	302.5
per 100 C9	**0.8**	**11.6**	**26.1**	**30.9**	**0.5**	**12.7**	**22.9**	**26.2**	**0.3**	**12.7**	**21.8**	**24.4**
**β-β native**												
Raw integral	22.9	24.5	13.4	12.5	37.1	31.0	16.7	19.4	54.9	35.1	25.3	22.7
per 100 C9	**7.4**	**4.3**	**1.7**	**1.4**	**8.1**	**4.1**	**2.0**	**1.9**	**8.1**	**3.2**	**2.1**	**1.8**
**β-β derived alcohol**												
Raw integral	0.3	6.7	15.9	5.7	0.8	11.6	14.2	10.2	1.3	19.1	21.2	10.6
per 100 C9	**0.1**	**1.2**	**2.0**	**0.6**	**0.2**	**1.5**	**1.7**	**1.0**	**0.2**	**1.8**	**1.8**	**0.9**
**β-β derived aldehyde**												
Raw integral	0.5	1.6	18.8	39.9	0.0	5.2	21.1	38.2	0.6	6.2	29.7	48.9
per 100 C9	**0.1**	**0.3**	**2.4**	**4.3**	**0.0**	**0.7**	**2.5**	**3.7**	**0.1**	**0.6**	**2.5**	**3.9**
**LBHK**												
Raw integral	17.2	33.2	36.2	29.2	36.0	60.7	52.5	48.7	48.1	86.7	77.0	64.9
per 100 C9	**5.5**	**5.8**	**4.6**	**3.2**	**7.9**	**8.1**	**6.3**	**4.7**	**7.1**	**8.0**	**6.5**	**5.2**

						**β-O-4^{OX} A(G)’**						
Raw integral	4.2	18.1	23.2	23.2	2.0	16.7	21.3	25.8	3.7	27.9	37.0	41.1
per 100 C9	**1.4**	**3.2**	**2.9**	**2.5**	**0.4**	**2.2**	**2.6**	**2.5**	**0.6**	**2.6**	**3.1**	**3.3**
						**β-O-4^{OX} A(G)”**						
Raw integral	0.3	19.7	60.3	83.7	0.8	23.9	47.9	69.4	2.3	35.6	71.1	81.5
per 100 C9	**0.1**	**3.5**	**7.6**	**9.1**	**0.2**	**3.2**	**5.7**	**6.7**	**0.3**	**3.3**	**6.0**	**6.6**
						**β-5**						
Raw integral	16.3	19.7	13.0	10.1	17.2	14.0	12.8	11.6	16.2	13.5	10.8	10.1
per 100 C9	**5.2**	**3.5**	**1.6**	**1.1**	**3.8**	**1.9**	**1.5**	**1.1**	**2.4**	**1.2**	**0.9**	**0.8**

Reproducibility, scalability and depolymerisation of Lignin $^{\alpha\text{-OX}}$

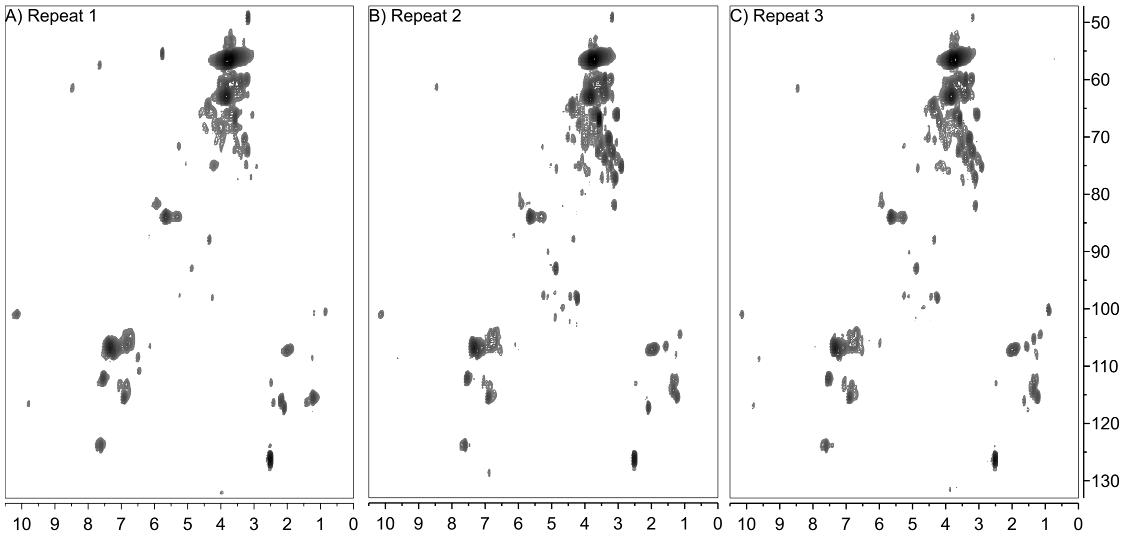

Figure S22: Reproducibility of lignin oxidation. 2D HSQC NMR analysis (700 MHz, d_6-DMSO) of beech lignin$^{\alpha\text{-OX}}$ generated from using 3 wt. eqv of DDQ oxidation. A) Repeat 1; B) Repeat 2 and; C) Repeat 3.

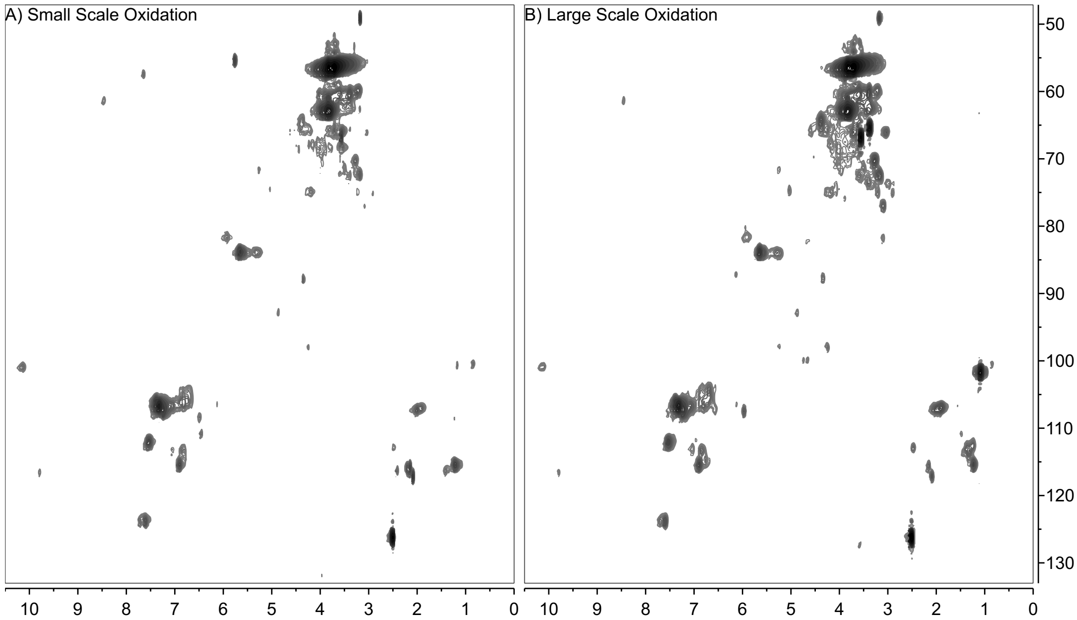

Figure S23: 2D HSQC NMR analysis (700 MHz, d_6-DMSO) of (A) Small scale oxidation (300 mg scale) and; (B) large scale oxidation (20 g scale).

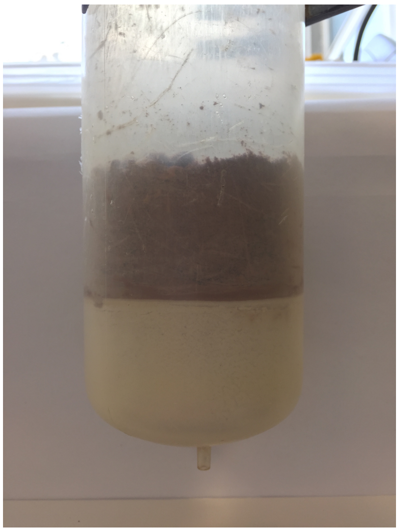

Figure S24: Recovery of DDQ-H_2 using celite packed fritted column. Celite is the bottom layer. DDQ-H_2 has deposited above the celite.

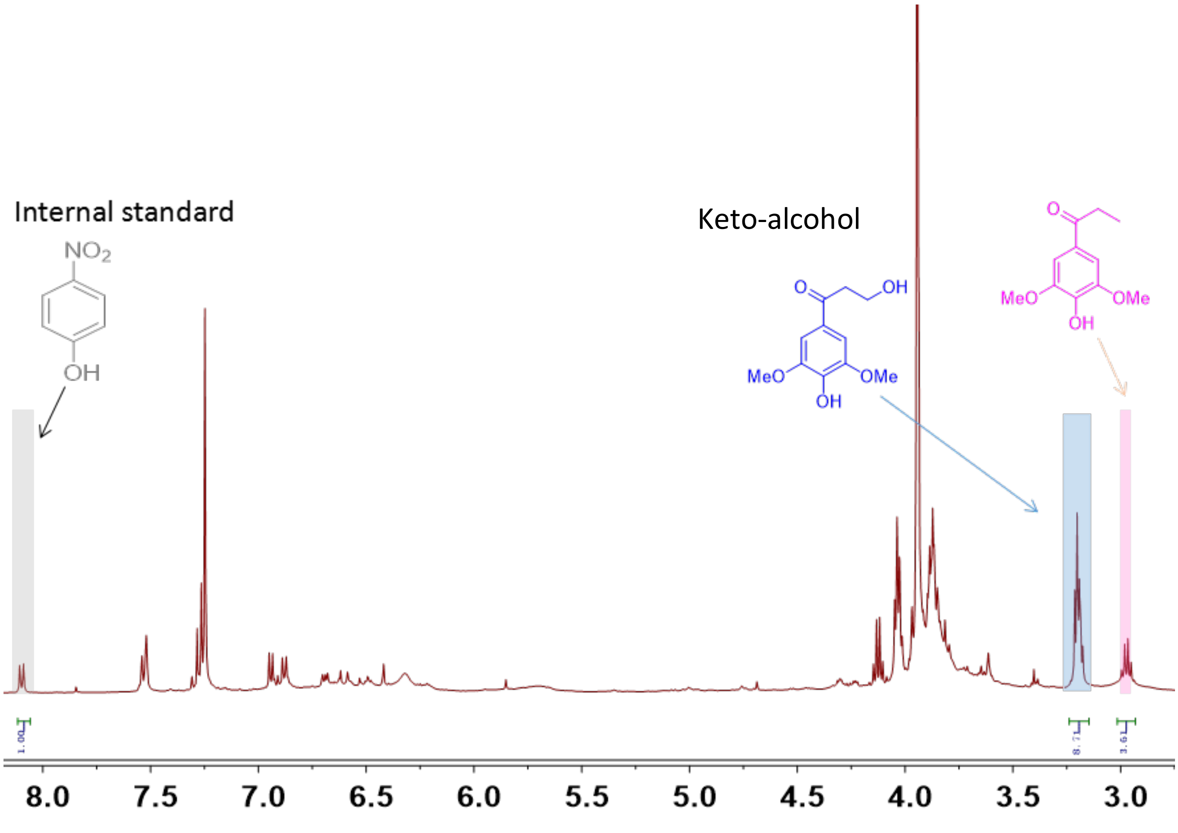

Figure S25: ^{1}H NMR ($CDCl_3$) of the crude reaction mixture from the zinc reductive cleavage of beech lignin$^{\alpha\text{-OX}}$. Highlighted are peaks corresponding to the keto-alcohol monomer as previously reported.[5]

References

1 F. Tran, C. S. Lancefield, P. C. J. Kamer, T. Lebl and N. J. Westwood, *Green Chem.*, 2014, **17**, 244–249.

2 R. S. Ward, A. Pelter, I. R. Jack, P. Satyanarayana, B. V Gopala Rao and P. Subrahmanyam, *Tetrahedron Lett.*, 1981, **22**, 4111–4114.

3 R. Venkateswarlu, C. Kamakshi, P. V. Subhash, S. G. A. Moinuddin, M. P. Gowri, R. S. Ward, A. Pelter, M. B. Hursthouse, S. J. Coles and M. E. Light, *Tetrahedron*, 2005, **61**, 8956–8961.

4 D. M. Miles-Barrett, J. R. D. Montgomery, C. S. Lancefield, D. B. Cordes, A. M. Z. Slawin, T. Lebl, R. Carr and N. J. Westwood, *ACS Sustain. Chem. Eng.*, 2016, **5**, 1831–1839.

5 C. S. Lancefield, O. S. Ojo, F. Tran and N. J. Westwood, *Angew. Chem. Int. Ed. Engl.*, 2015, **54**, 258–262.

6 A. Rahimi, A. Azarpira, H. Kim, J. Ralph and S. S. Stahl, *J. Am. Chem. Soc.*, 2013, **135**, 6415–8.

7 D. M. Miles-Barrett, A. R. Neal, C. Hand, J. R. D. Montgomery, I. Panovic, O. S. Ojo, C. S. Lancefield, D. B. Cordes, A. M. Z. Slawin, T. Lebl and N. J. Westwood, *Org. Biomol. Chem.*, 2016, **14**, 10023–10030.

8 R. Evans, Z. Deng, A. K. Rogerson, A. S. McLachlan, J. J. Richards, M. Nilsson and G. A. Morris, *Angew. Chemie - Int. Ed.*, 2013, **52**, 3199–3202.

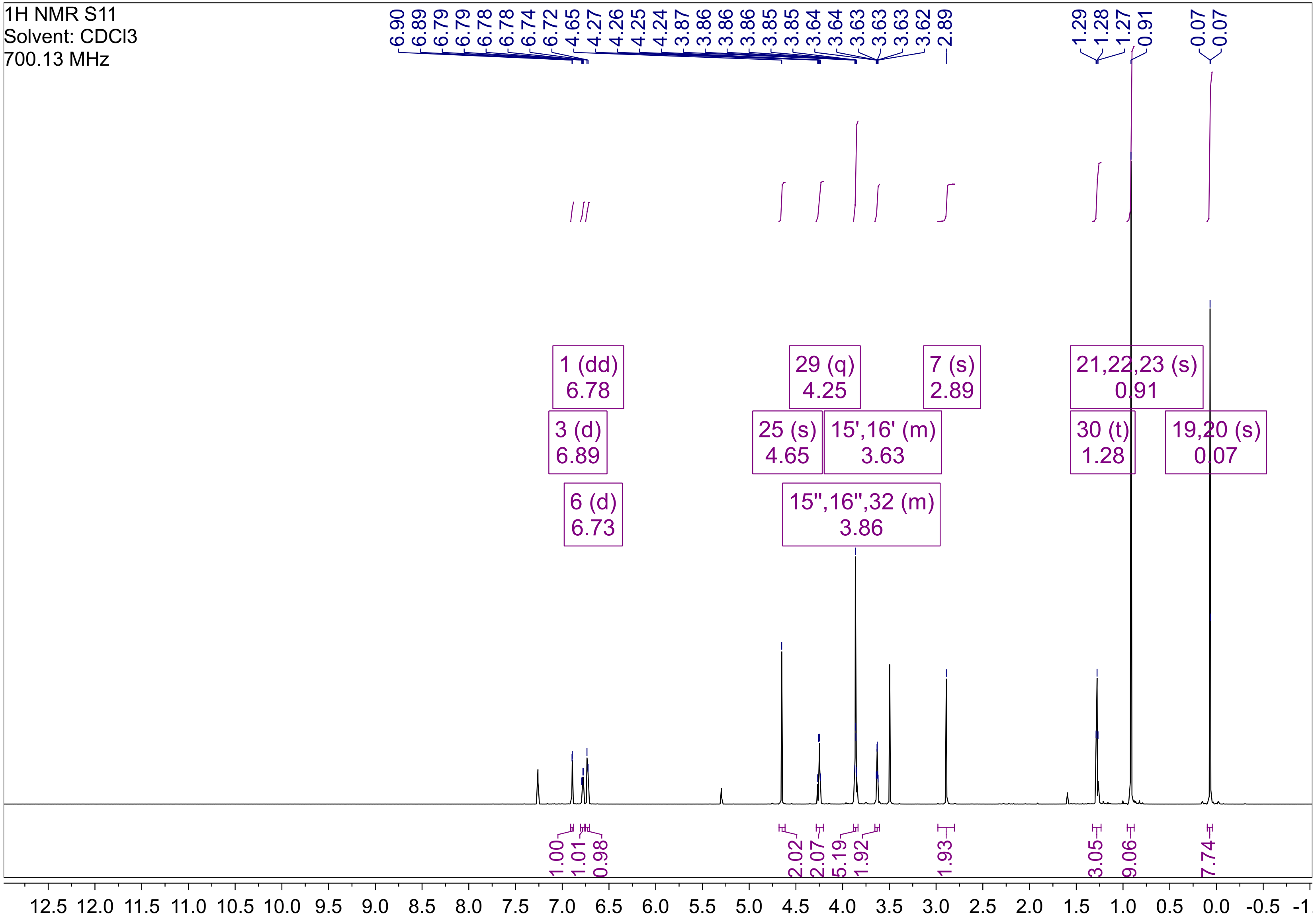
1H NMR S11
Solvent: CDCl3
700.13 MHz
6.90 6.89 6.79 6.79 6.78 6.78 6.74 6.72 4.65 4.27 4.26 4.25 4.24 3.87 3.86 3.86 3.86 3.85 3.85 3.64 3.64 3.63 3.63 3.63 3.62 2.89
1.29 1.28 1.27 0.91
0.07 0.07
1 (dd) 6.78
3 (d) 6.89
6 (d) 6.73
29 (q) 4.25
25 (s) 4.65
15',16' (m) 3.63
15'',16'',32 (m) 3.86
7 (s) 2.89
21,22,23 (s) 0.91
30 (t) 1.28
19,20 (s) 0.07
1.00 1.01 0.98 2.02 2.07 5.19 1.92 1.93 3.05 9.06 7.74
12.5 12.0 11.5 11.0 10.5 10.0 9.5 9.0 8.5 8.0 7.5 7.0 6.5 6.0 5.5 5.0 4.5 4.0 3.5 3.0 2.5 2.0 1.5 1.0 0.5 0.0 -0.5

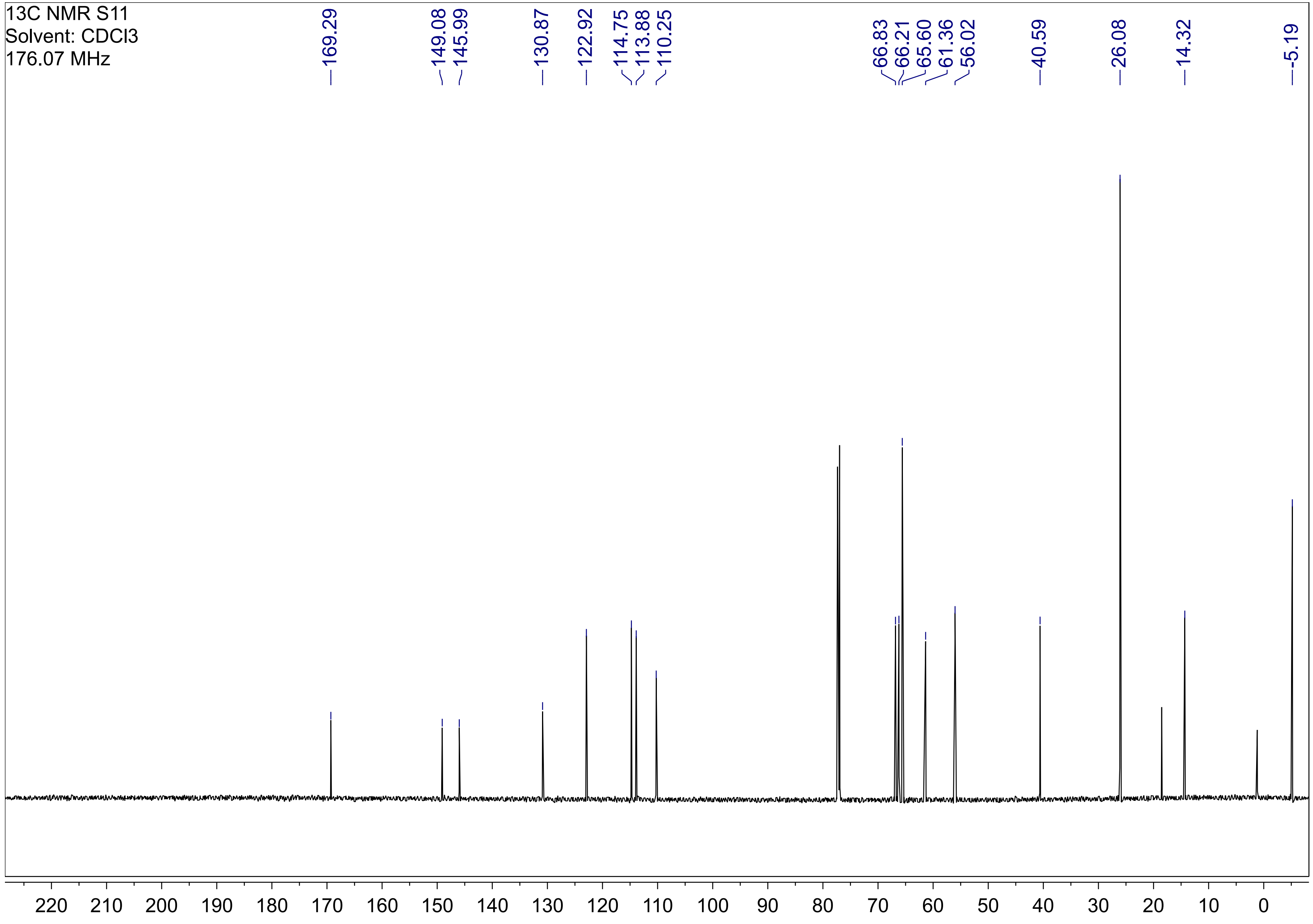
13C NMR S11
Solvent: CDCl3
176.07 MHz
169.29
149.08
145.99
130.87
122.92
114.75
113.88
110.25
66.83
66.21
65.60
61.36
56.02
40.59
26.08
14.32
-5.19
220 210 200 190 180 170 160 150 140 130 120 110 100 90 80 70 60 50 40 30 20 10 0

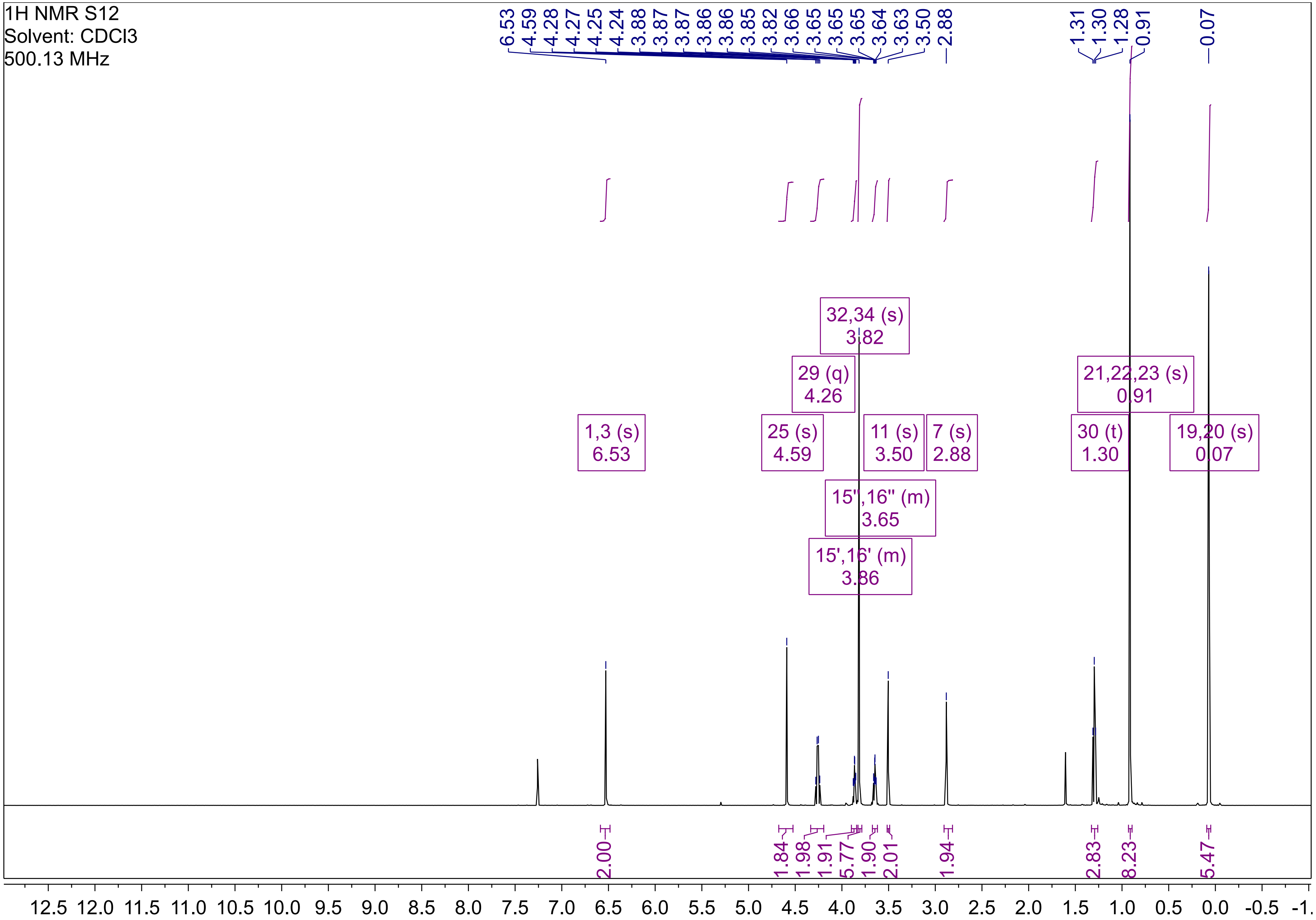
1H NMR S12
Solvent: CDCl3
500.13 MHz
6.53
4.59
4.28
4.27
4.25
4.24
3.88
3.87
3.87
3.86
3.86
3.85
3.82
3.66
3.65
3.65
3.65
3.64
3.63
3.50
2.88
1.31
1.30
1.28
0.91
0.07
32,34 (s)
3.82
29 (q)
4.26
21,22,23 (s)
0.91
1,3 (s)
6.53
25 (s)
4.59
11 (s)
3.50
7 (s)
2.88
30 (t)
1.30
19,20 (s)
0.07
15'',16'' (m)
3.65
15',16' (m)
3.86
2.00
1.84
1.98
1.91
5.77
1.90
2.01
1.94
2.83
8.23
5.47
12.5 12.0 11.5 11.0 10.5 10.0 9.5 9.0 8.5 8.0 7.5 7.0 6.5 6.0 5.5 5.0 4.5 4.0 3.5 3.0 2.5 2.0 1.5 1.0 0.5 0.0 -0.5 -1.

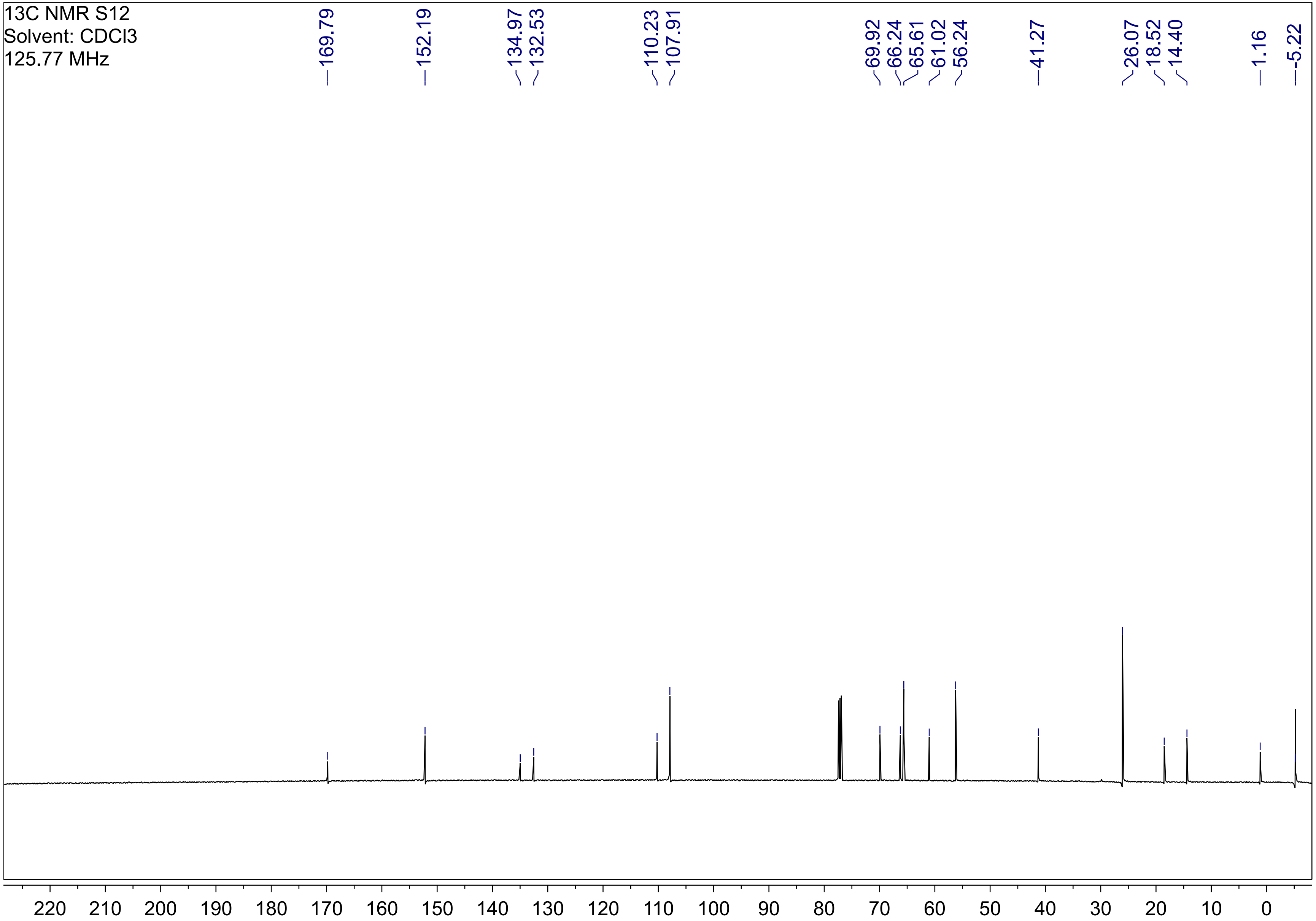
13C NMR S12
Solvent: CDCl3
125.77 MHz
169.79
152.19
134.97
132.53
110.23
107.91
69.92
66.24
65.61
61.02
56.24
41.27
26.07
18.52
14.40
-1.16
-5.22
220 210 200 190 180 170 160 150 140 130 120 110 100 90 80 70 60 50 40 30 20 10 0

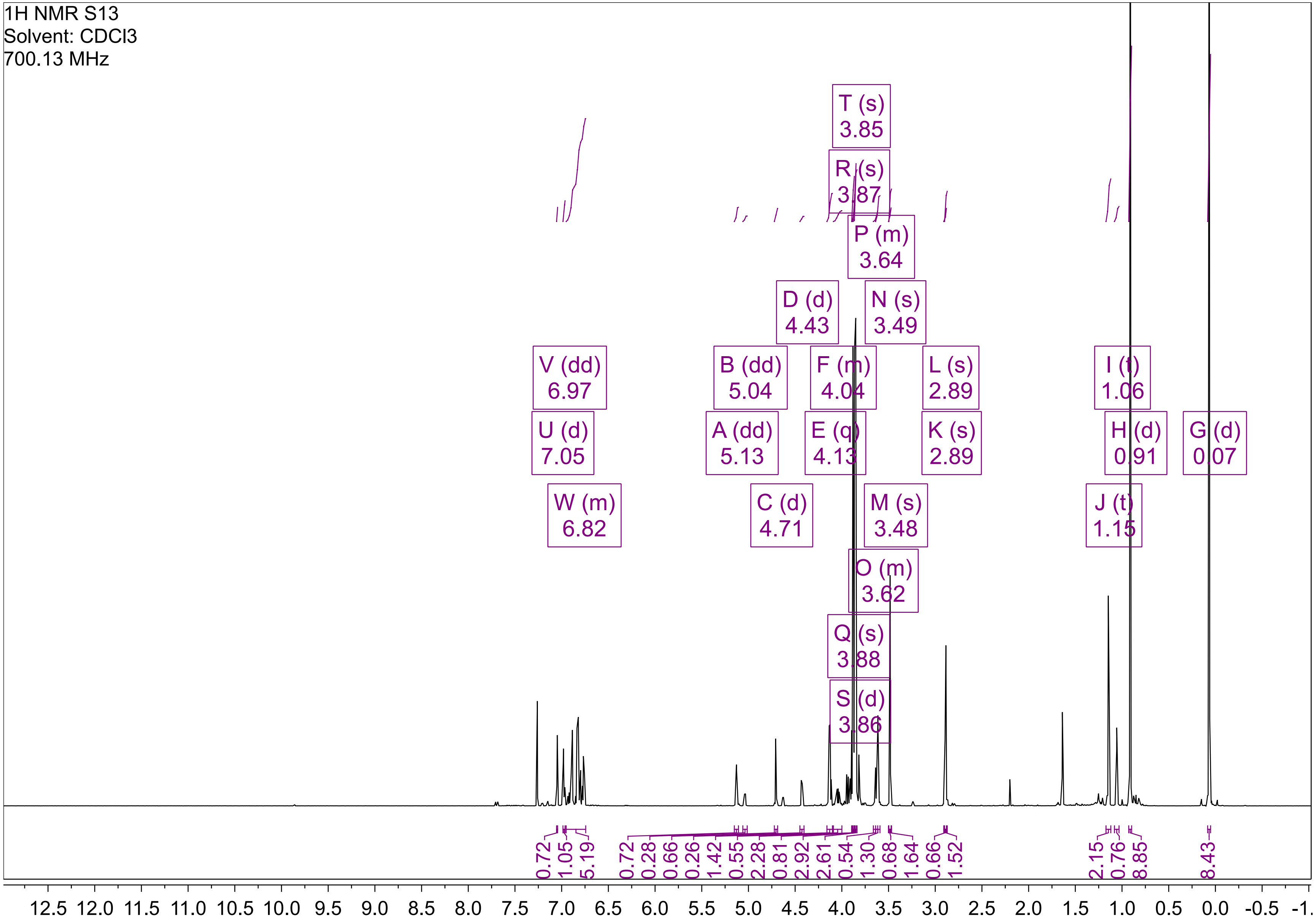
1H NMR S13
Solvent: CDCl3
700.13 MHz
T (s) 3.85
R (s) 3.87
P (m) 3.64
D (d) 4.43
N (s) 3.49
V (dd) 6.97
B (dd) 5.04
F (m) 4.04
L (s) 2.89
I (t) 1.06
U (d) 7.05
A (dd) 5.13
E (q) 4.13
K (s) 2.89
H (d) 0.91
G (d) 0.07
W (m) 6.82
C (d) 4.71
M (s) 3.48
J (t) 1.15
O (m) 3.62
Q (s) 3.88
S (d) 3.86
0.72 1.05 5.19 0.72 0.28 0.66 0.26 1.42 0.55 2.28 0.81 2.92 2.61 0.54 1.30 0.68 1.64 0.66 1.52 2.15 0.76 8.85 8.43
12.5 12.0 11.5 11.0 10.5 10.0 9.5 9.0 8.5 8.0 7.5 7.0 6.5 6.0 5.5 5.0 4.5 4.0 3.5 3.0 2.5 2.0 1.5 1.0 0.5 0.0 -0.5 -1.

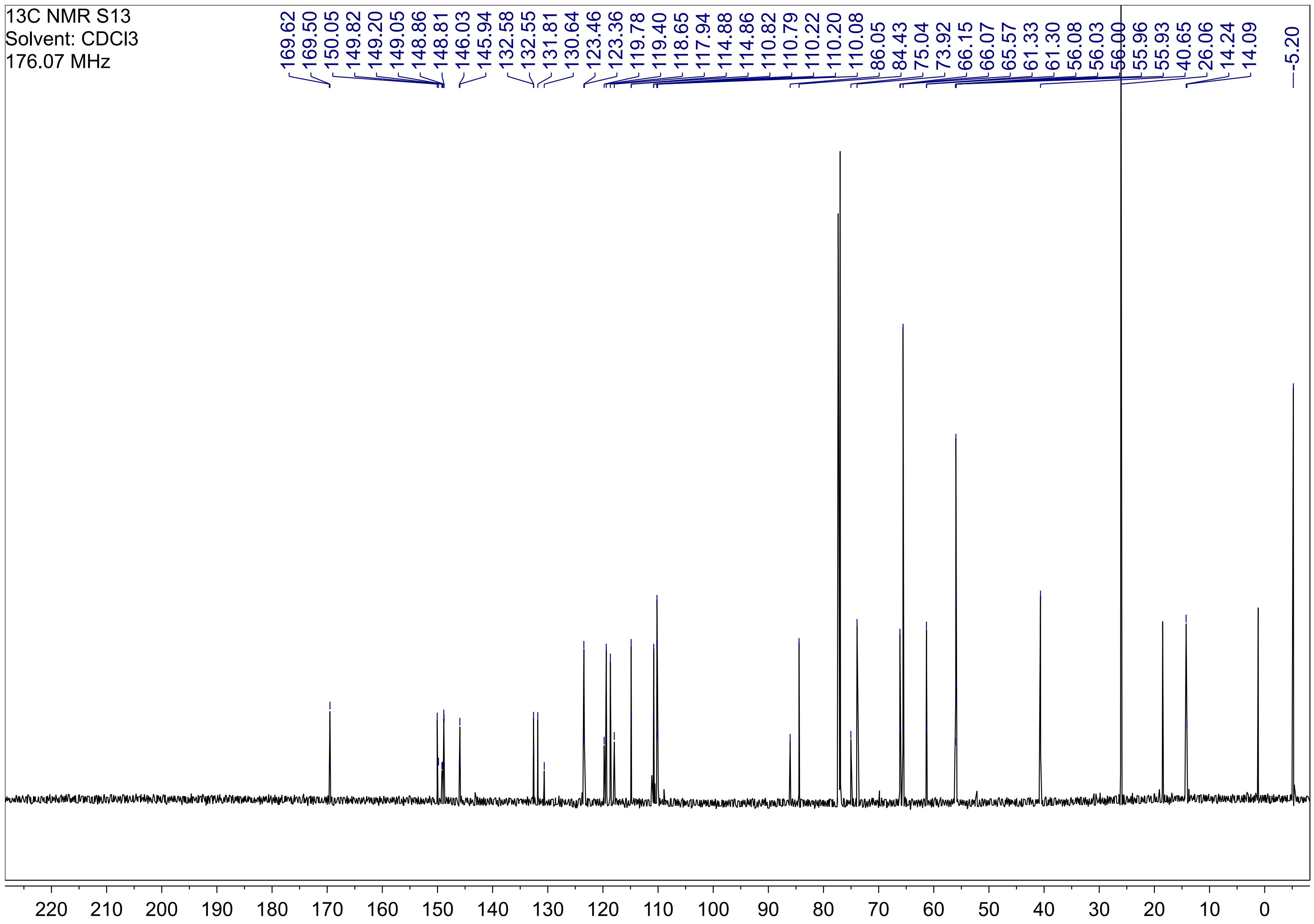
13C NMR S13
Solvent: CDCl3
176.07 MHz
169.62
169.50
150.05
149.82
149.20
149.05
148.86
148.81
146.03
145.94
132.58
132.55
131.81
130.64
123.46
123.36
119.78
119.40
118.65
117.94
114.88
114.86
110.82
110.79
110.22
110.20
110.08
86.05
84.43
75.04
73.92
66.15
66.07
65.57
61.33
61.30
56.08
56.03
56.00
55.96
55.93
40.65
26.06
14.24
14.09
-5.20
220
210
200
190
180
170
160
150
140
130
120
110
100
90
80
70
60
50
40
30
20
10
0

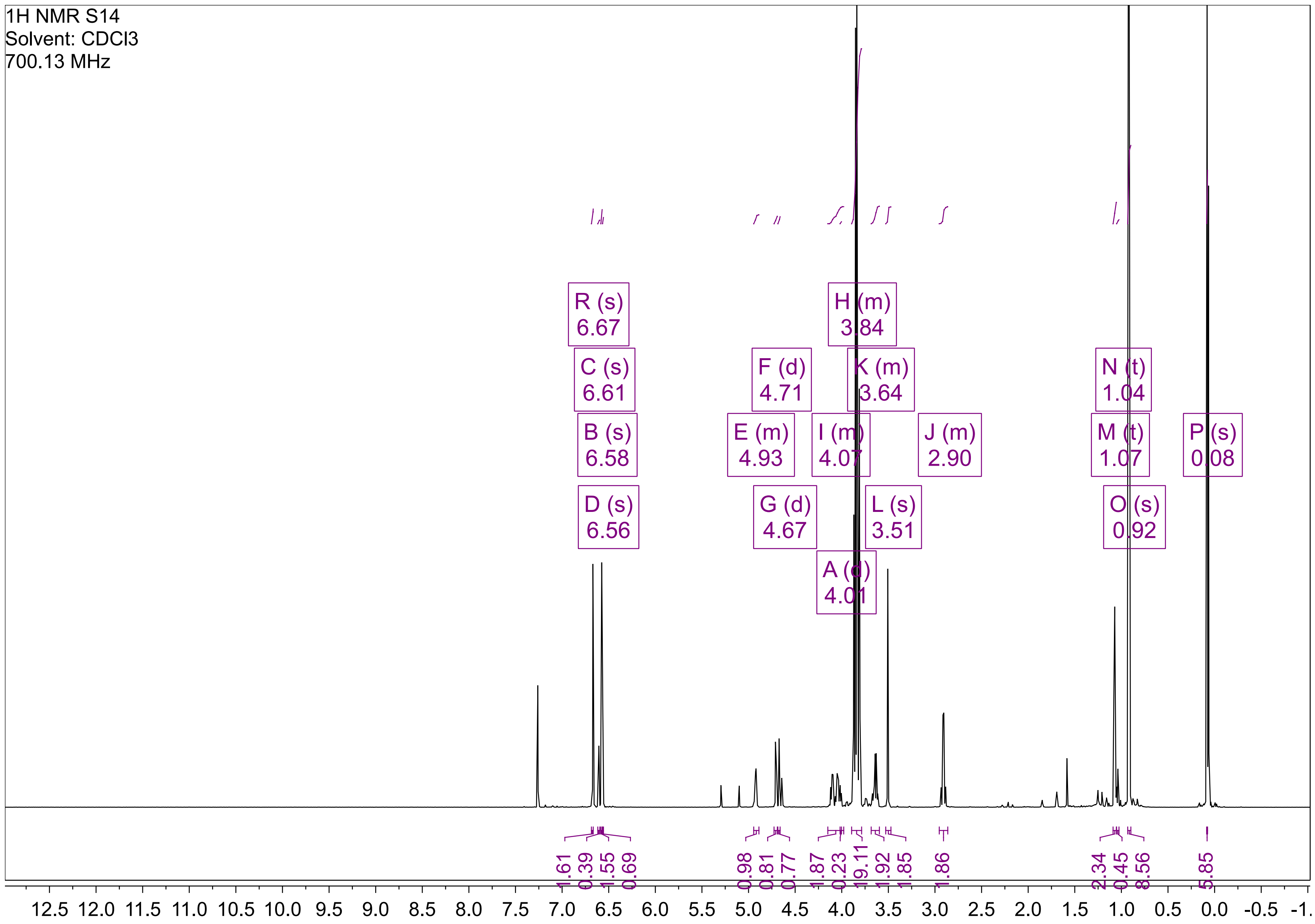
1H NMR S14
Solvent: CDCl3
700.13 MHz
R (s) 6.67
C (s) 6.61
B (s) 6.58
D (s) 6.56
E (m) 4.93
F (d) 4.71
G (d) 4.67
H (m) 3.84
I (m) 4.07
A (d) 4.01
K (m) 3.64
L (s) 3.51
J (m) 2.90
N (t) 1.04
M (t) 1.07
O (s) 0.92
P (s) 0.08
1.61
0.39
1.55
0.69
0.98
0.81
0.77
1.87
0.23
19.11
1.92
1.85
1.86
2.34
0.45
8.56
5.85
12.5 12.0 11.5 11.0 10.5 10.0 9.5 9.0 8.5 8.0 7.5 7.0 6.5 6.0 5.5 5.0 4.5 4.0 3.5 3.0 2.5 2.0 1.5 1.0 0.5 0.0 -0.5

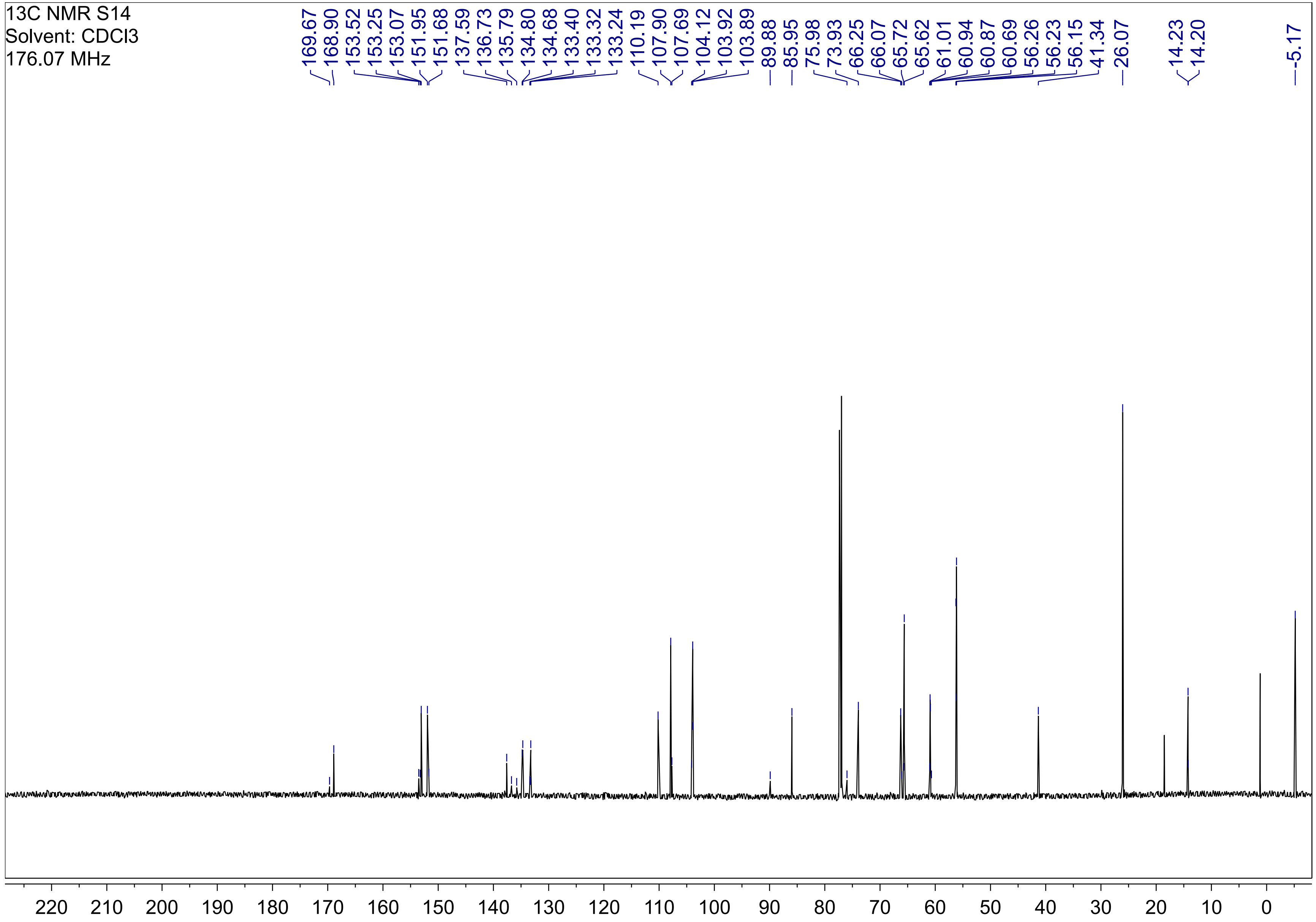
13C NMR S14
Solvent: CDCl3
176.07 MHz
169.67
168.90
153.52
153.25
153.07
151.95
151.68
137.59
136.73
135.79
134.80
134.68
133.40
133.32
133.24
110.19
107.90
107.69
104.12
103.92
103.89
89.88
85.95
75.98
73.93
66.25
66.07
65.72
65.62
61.01
60.94
60.87
60.69
56.26
56.23
56.15
41.34
26.07
14.23
14.20
-5.17
220
210
200
190
180
170
160
150
140
130
120
110
100
90
80
70
60
50
40
30
20
10
0

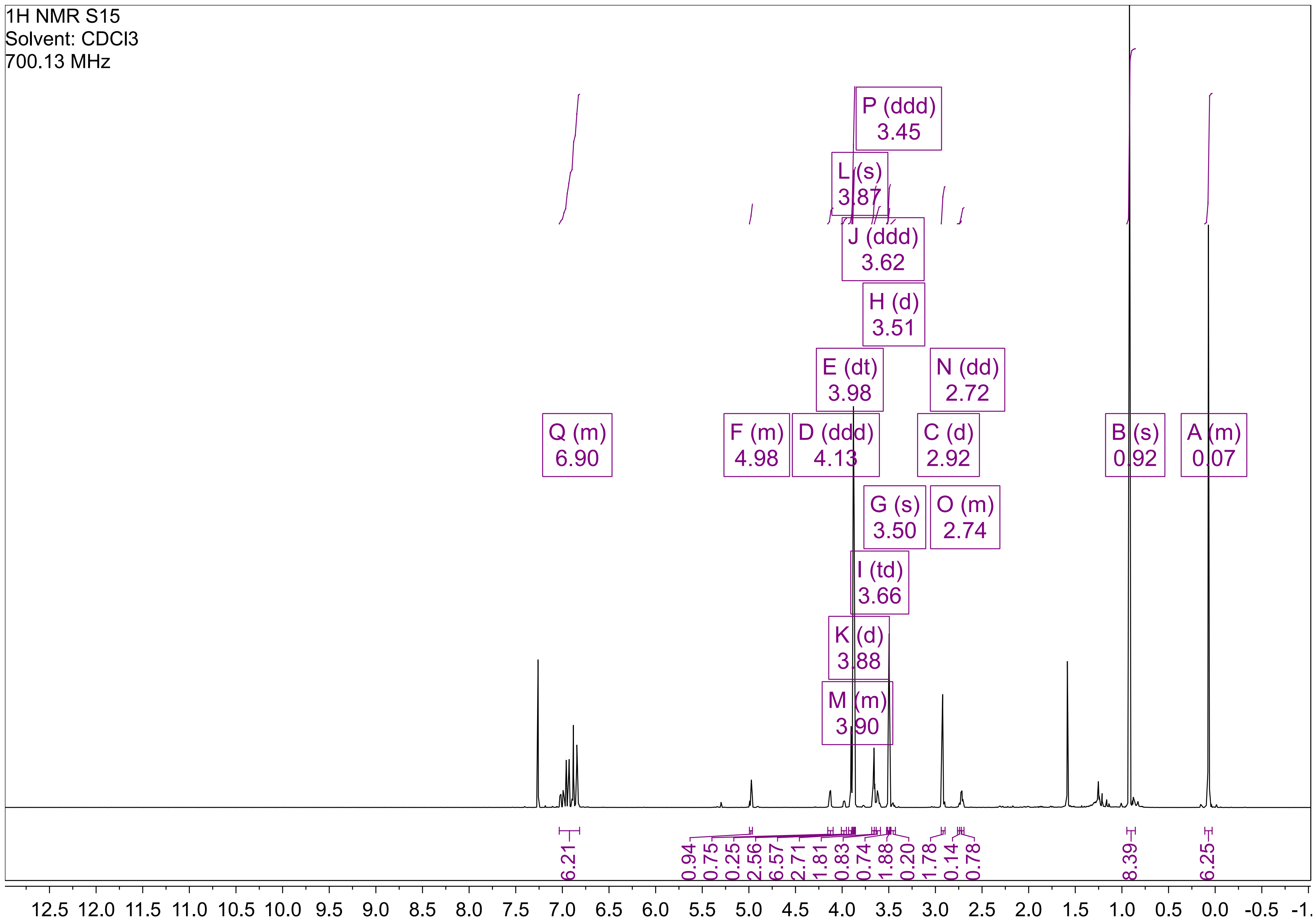
1H NMR S15
Solvent: CDCl3
700.13 MHz
P (ddd) 3.45
L (s) 3.87
J (ddd) 3.62
H (d) 3.51
E (dt) 3.98
N (dd) 2.72
Q (m) 6.90
F (m) 4.98
D (ddd) 4.13
C (d) 2.92
B (s) 0.92
A (m) 0.07
G (s) 3.50
O (m) 2.74
I (td) 3.66
K (d) 3.88
M (m) 3.90
6.21
0.94
0.75
0.25
2.56
6.57
2.71
1.81
0.83
0.74
1.88
0.20
1.78
0.14
0.78
8.39
6.25
12.5 12.0 11.5 11.0 10.5 10.0 9.5 9.0 8.5 8.0 7.5 7.0 6.5 6.0 5.5 5.0 4.5 4.0 3.5 3.0 2.5 2.0 1.5 1.0 0.5 0.0 -0.5 -1.

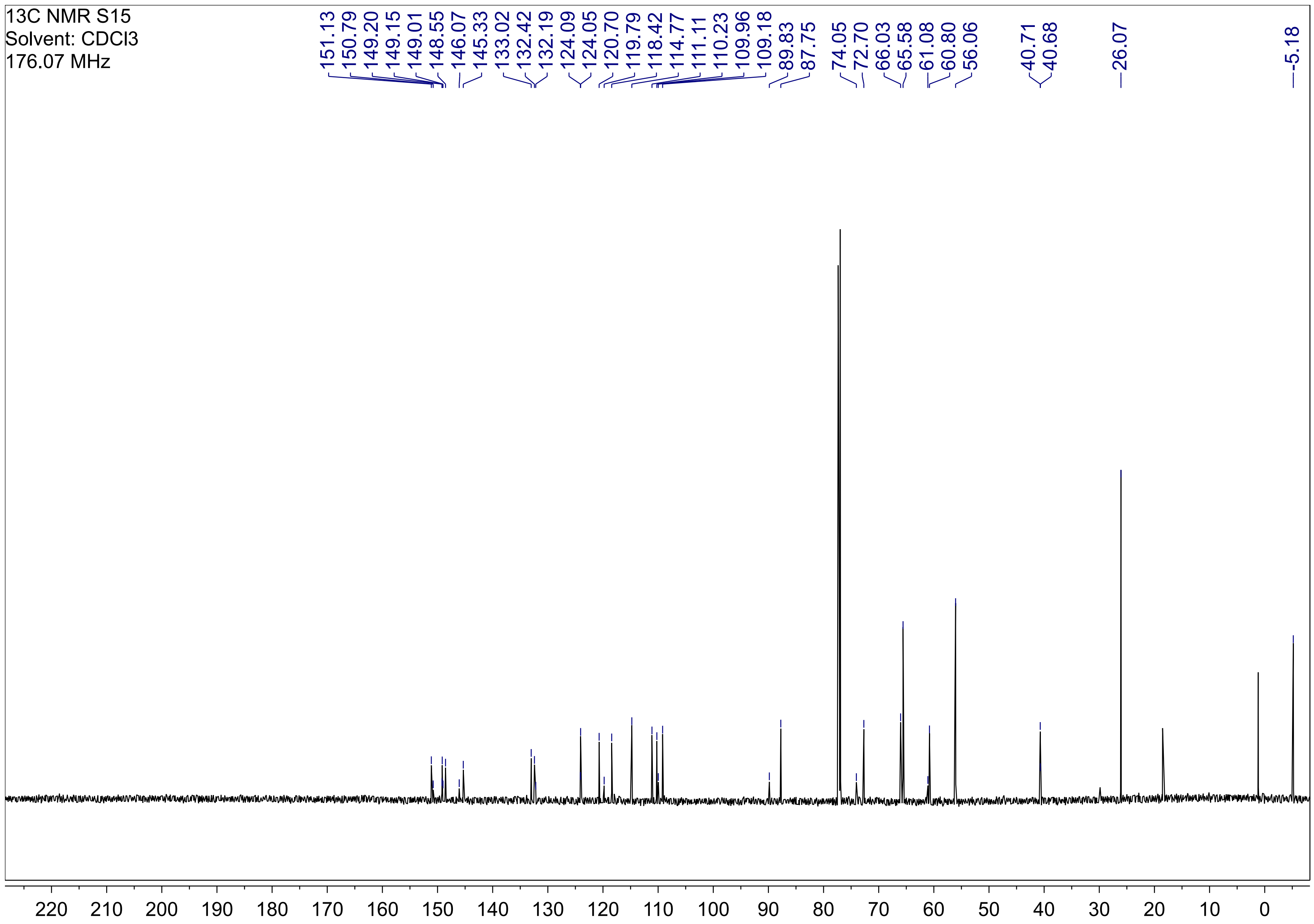
13C NMR S15
Solvent: CDCl3
176.07 MHz
151.13
150.79
149.20
149.15
149.01
148.55
146.07
145.33
133.02
132.42
132.19
124.09
124.05
120.70
119.79
118.42
114.77
111.11
110.23
109.96
109.18
89.83
87.75
74.05
72.70
66.03
65.58
61.08
60.80
56.06
40.71
40.68
26.07
-5.18
220 210 200 190 180 170 160 150 140 130 120 110 100 90 80 70 60 50 40 30 20 10 0

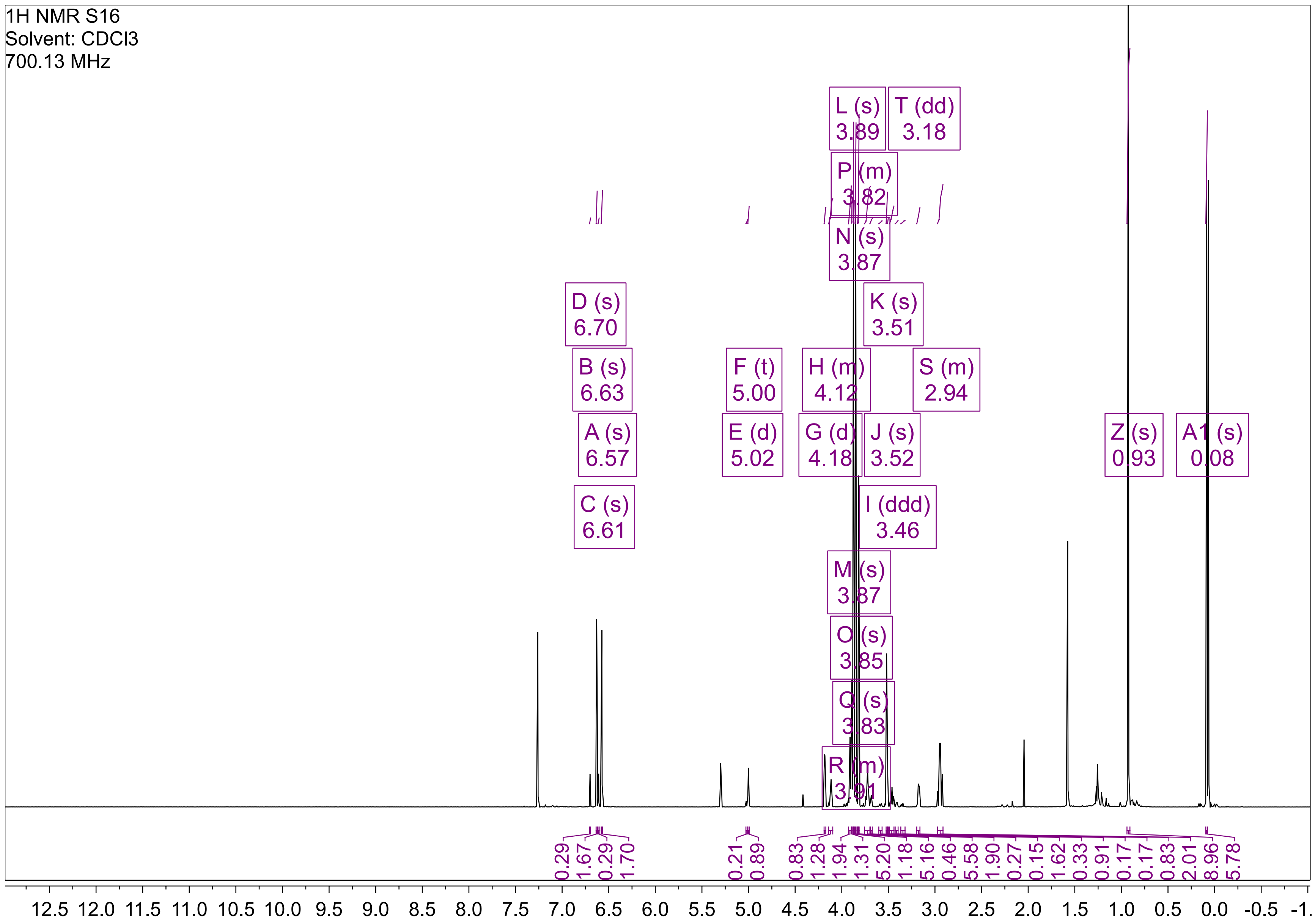
1H NMR S16
Solvent: CDCl3
700.13 MHz

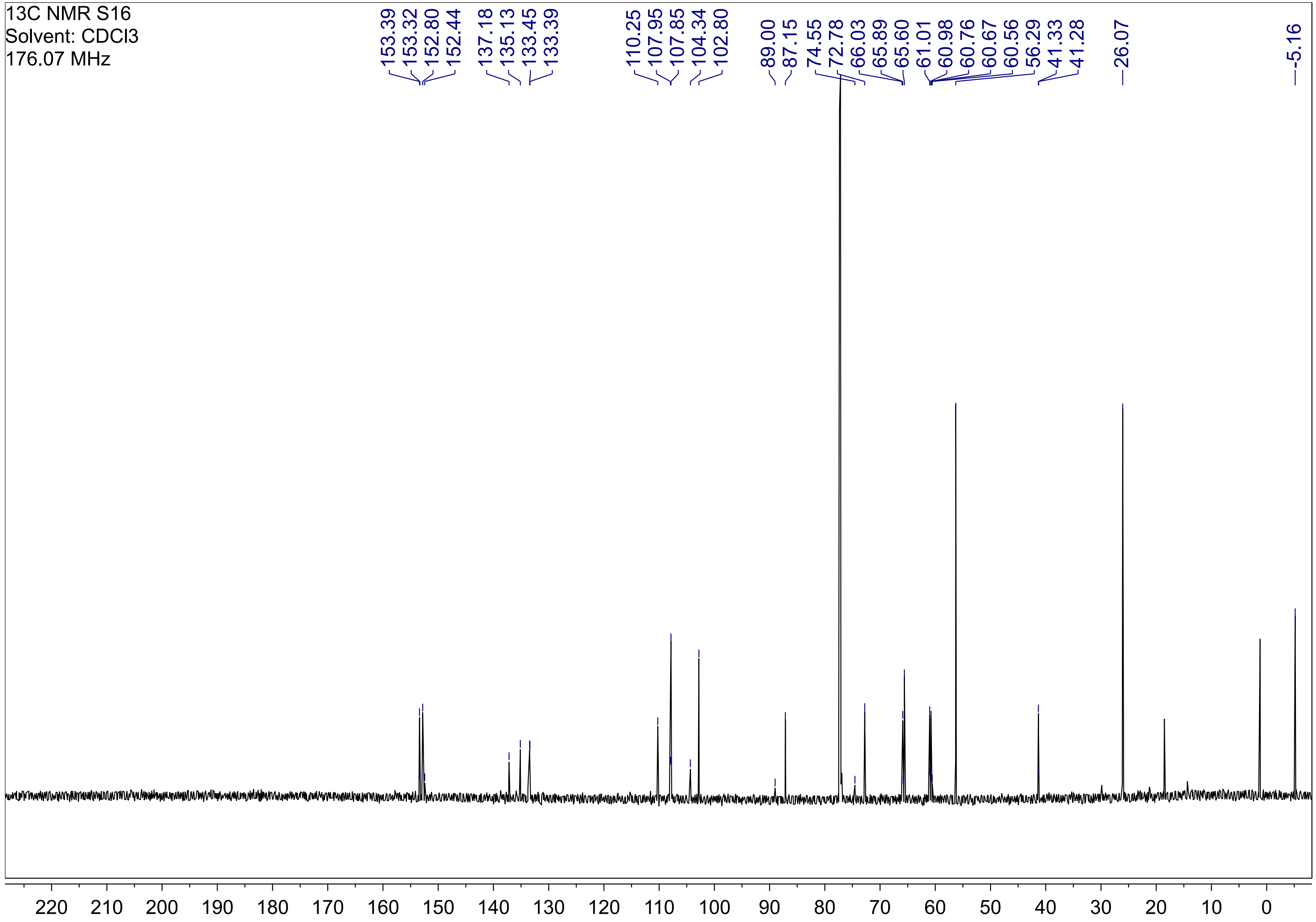
13C NMR S16
Solvent: CDCl3
176.07 MHz
153.39
153.32
152.80
152.44
137.18
135.13
133.45
133.39
110.25
107.95
107.85
104.34
102.80
89.00
87.15
74.55
72.78
66.03
65.89
65.60
61.01
60.98
60.76
60.67
60.56
56.29
41.33
41.28
26.07
-5.16
220
210
200
190
180
170
160
150
140
130
120
110
100
90
80
70
60
50
40
30
20
10
0

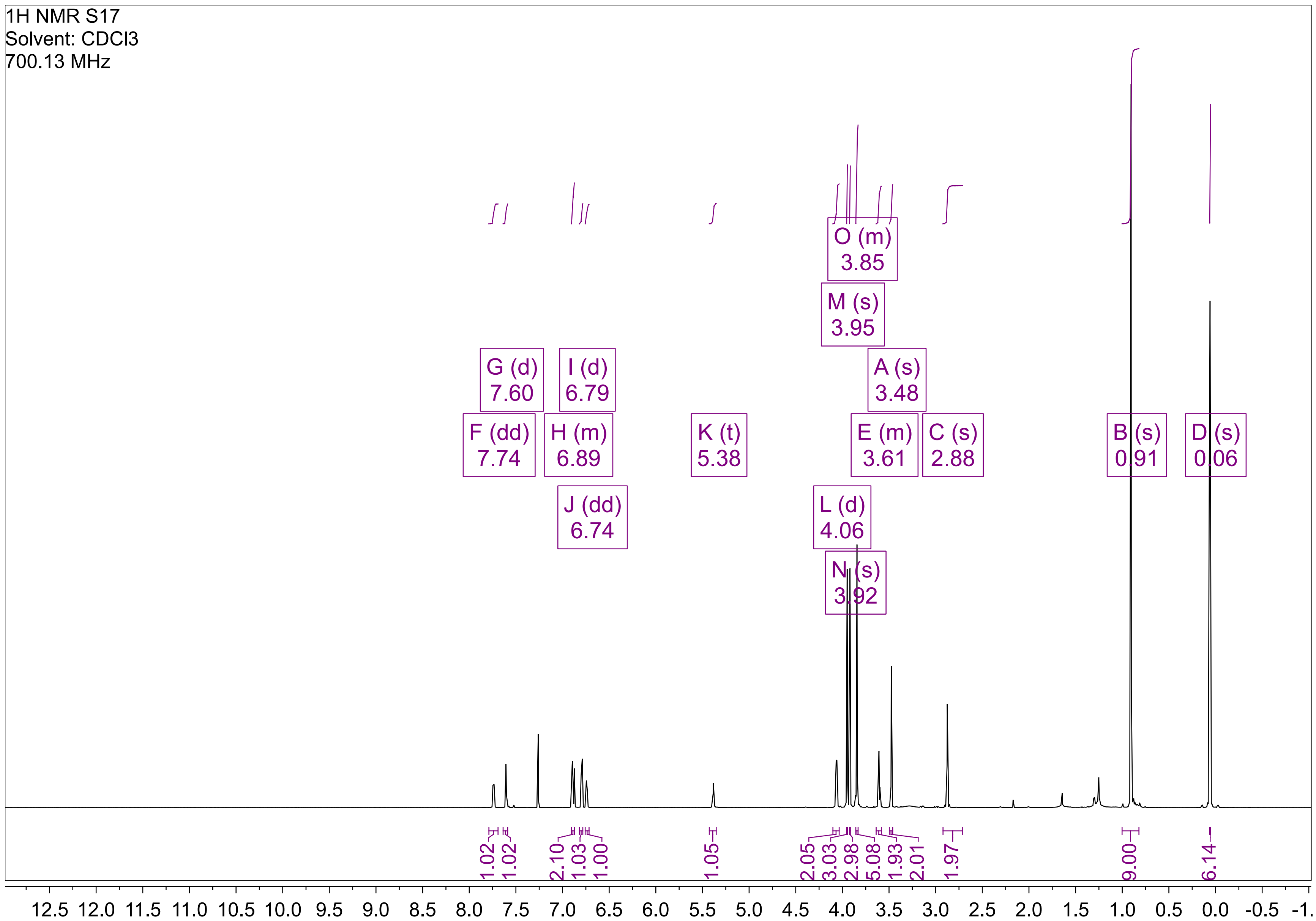
1H NMR S17
Solvent: CDCl3
700.13 MHz
O (m) 3.85
M (s) 3.95
G (d) 7.60
I (d) 6.79
A (s) 3.48
F (dd) 7.74
H (m) 6.89
K (t) 5.38
E (m) 3.61
C (s) 2.88
B (s) 0.91
D (s) 0.06
J (dd) 6.74
L (d) 4.06
N (s) 3.92
1.02
1.02
2.10
1.03
1.00
1.05
2.05
3.03
2.98
5.08
1.93
2.01
1.97
9.00
6.14
12.5 12.0 11.5 11.0 10.5 10.0 9.5 9.0 8.5 8.0 7.5 7.0 6.5 6.0 5.5 5.0 4.5 4.0 3.5 3.0 2.5 2.0 1.5 1.0 0.5 0.0 -0.5 -1.

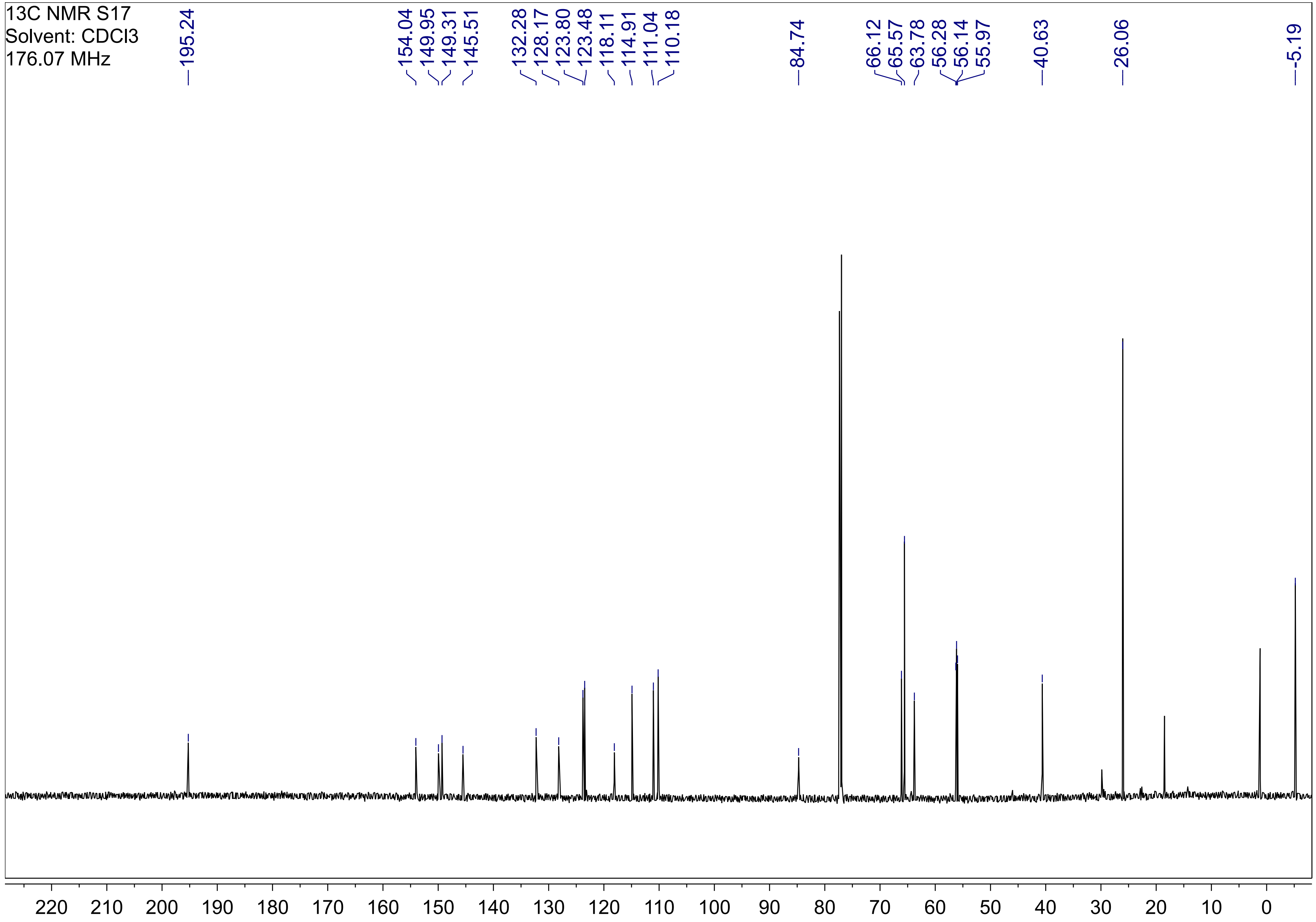
13C NMR S17
Solvent: CDCl3
176.07 MHz
195.24
154.04
149.95
149.31
145.51
132.28
128.17
123.80
123.48
118.11
114.91
111.04
110.18
84.74
66.12
65.57
63.78
56.28
56.14
55.97
40.63
26.06
-5.19
220 210 200 190 180 170 160 150 140 130 120 110 100 90 80 70 60 50 40 30 20 10 0

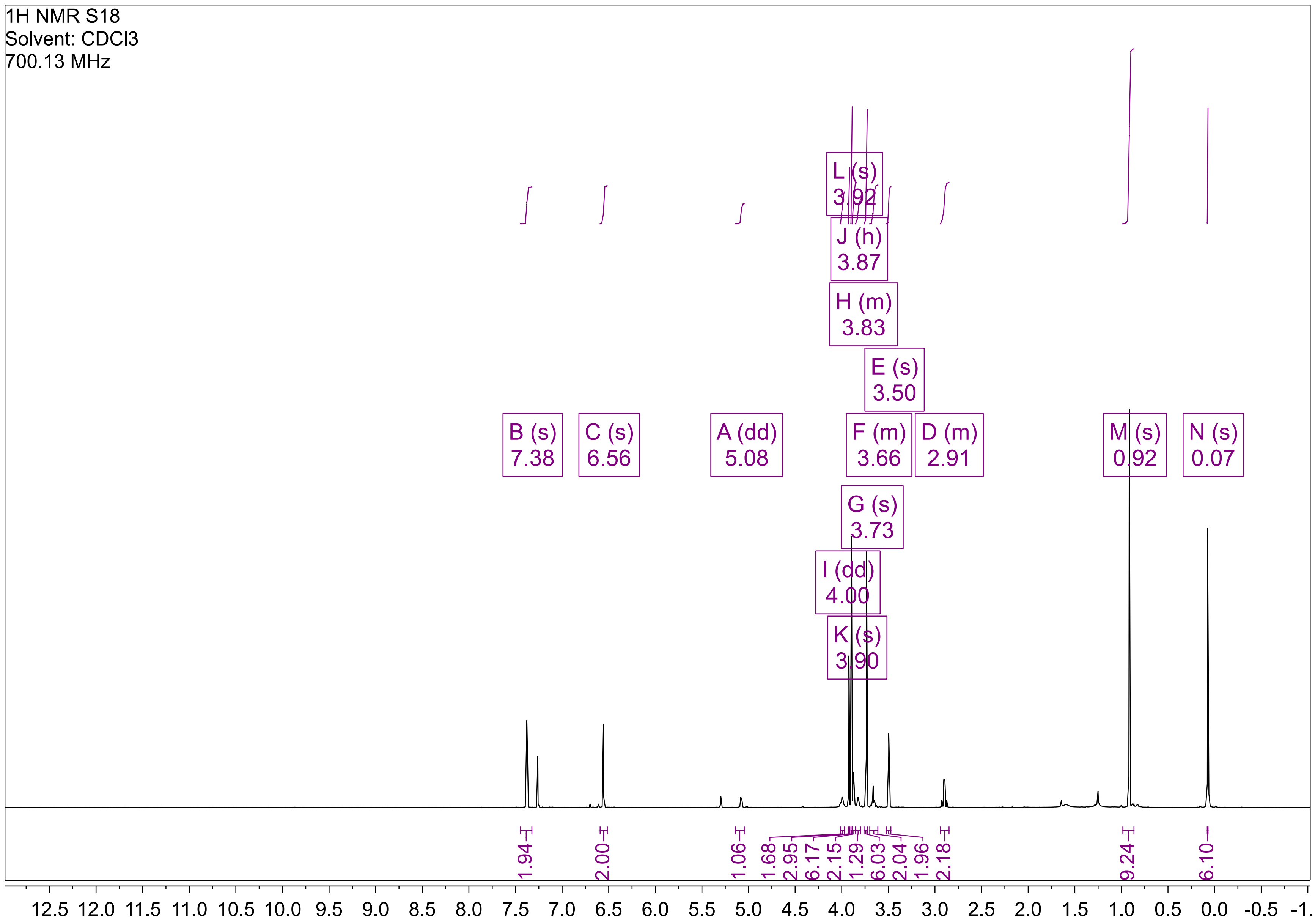
1H NMR S18
Solvent: CDCl3
700.13 MHz
B (s) 7.38
C (s) 6.56
A (dd) 5.08
L (s) 3.92
J (h) 3.87
H (m) 3.83
E (s) 3.50
F (m) 3.66
D (m) 2.91
G (s) 3.73
I (dd) 4.00
K (s) 3.90
M (s) 0.92
N (s) 0.07
1.94
2.00
1.06
1.68
2.95
6.17
2.15
1.29
6.03
2.04
1.96
2.18
9.24
6.10
12.5 12.0 11.5 11.0 10.5 10.0 9.5 9.0 8.5 8.0 7.5 7.0 6.5 6.0 5.5 5.0 4.5 4.0 3.5 3.0 2.5 2.0 1.5 1.0 0.5 0.0 -0.5 -1.

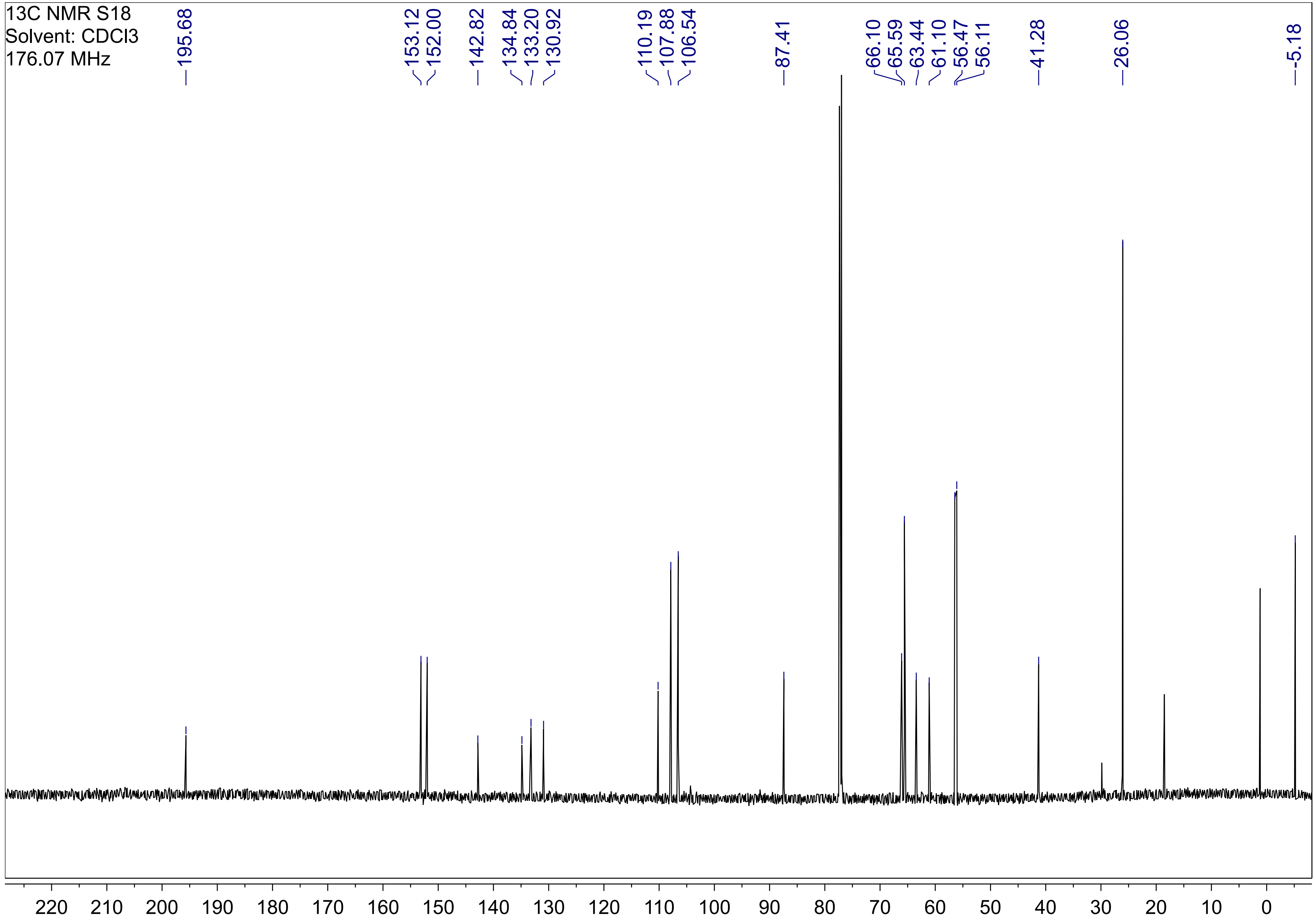
13C NMR S18
Solvent: CDCl3
176.07 MHz
195.68
153.12
152.00
142.82
134.84
133.20
130.92
110.19
107.88
106.54
87.41
66.10
65.59
63.44
61.10
56.47
56.11
41.28
26.06
-5.18
220 210 200 190 180 170 160 150 140 130 120 110 100 90 80 70 60 50 40 30 20 10 0

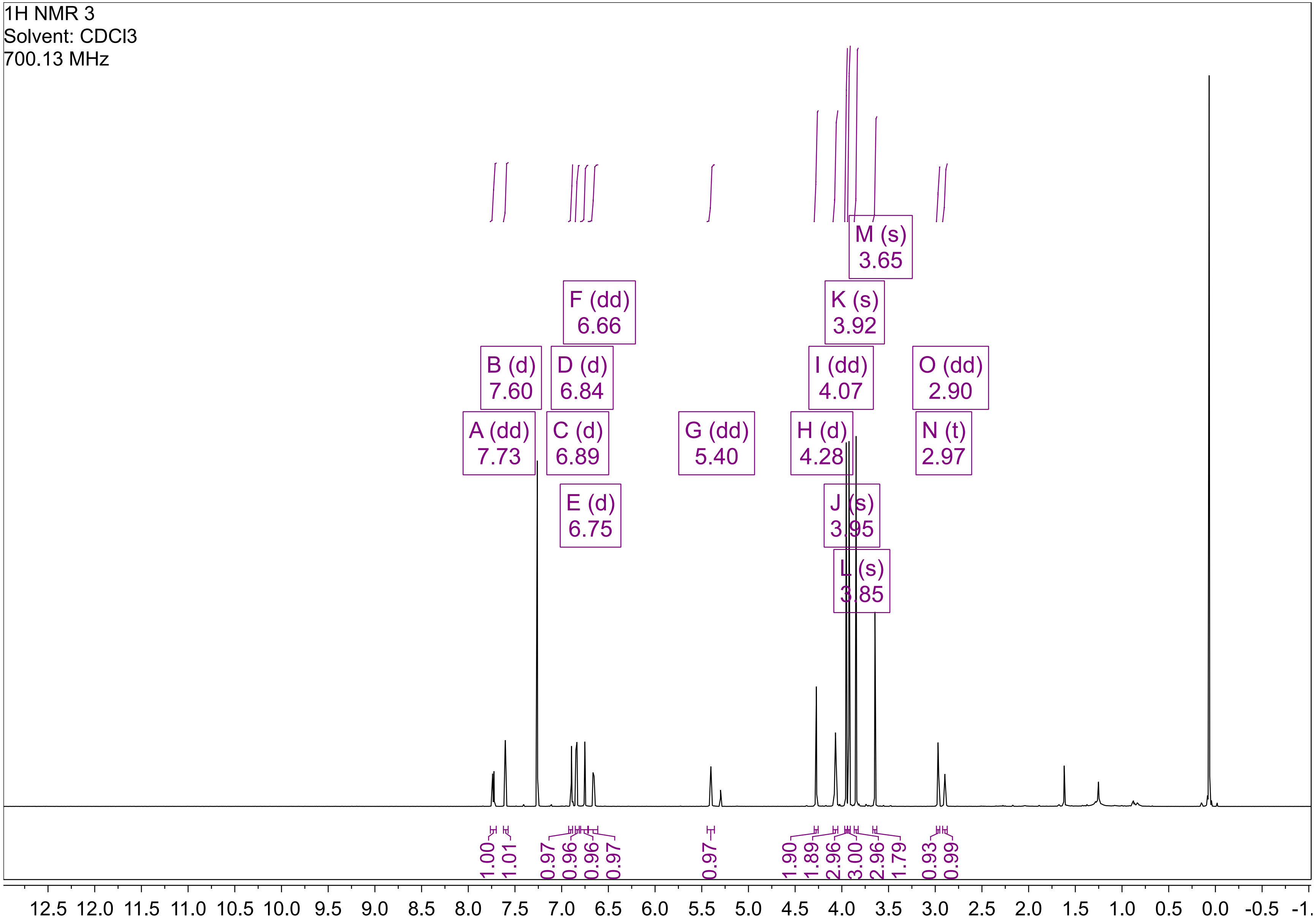
1H NMR 3
Solvent: CDCl3
700.13 MHz
M (s) 3.65
F (dd) 6.66
K (s) 3.92
B (d) 7.60
D (d) 6.84
I (dd) 4.07
O (dd) 2.90
A (dd) 7.73
C (d) 6.89
G (dd) 5.40
H (d) 4.28
N (t) 2.97
E (d) 6.75
J (s) 3.95
L (s) 3.85
1.00
1.01
0.97
0.96
0.96
0.97
0.97
1.90
1.89
2.96
3.00
2.96
1.79
0.93
0.99
12.5 12.0 11.5 11.0 10.5 10.0 9.5 9.0 8.5 8.0 7.5 7.0 6.5 6.0 5.5 5.0 4.5 4.0 3.5 3.0 2.5 2.0 1.5 1.0 0.5 0.0 -0.5 -1.

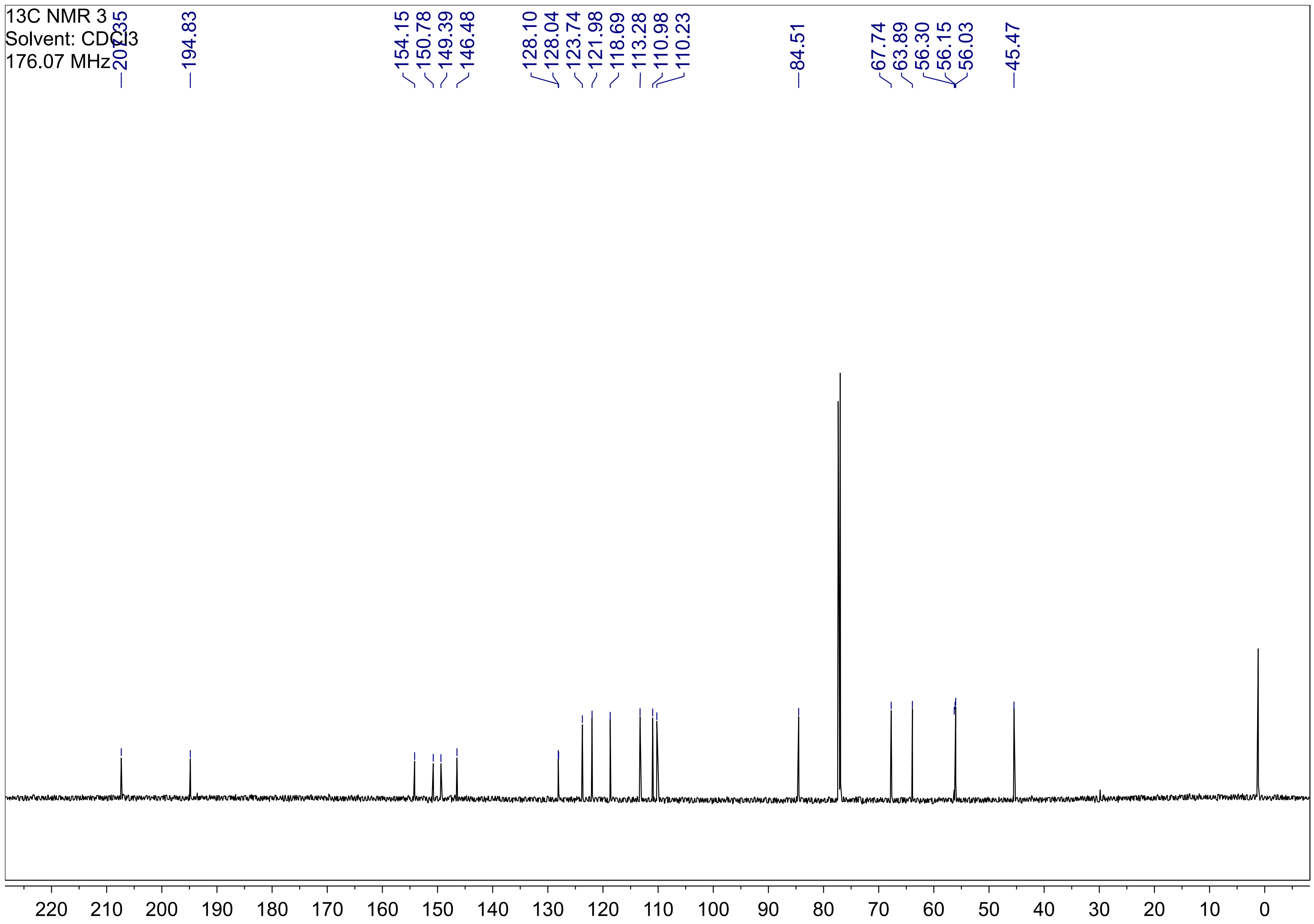

13C NMR 3
Solvent: CDCl3
176.07 MHz
207.35
194.83
154.15
150.78
149.39
146.48
128.10
128.04
123.74
121.98
118.69
113.28
110.98
110.23
84.51
67.74
63.89
56.30
56.15
56.03
45.47
220 210 200 190 180 170 160 150 140 130 120 110 100 90 80 70 60 50 40 30 20 10 0

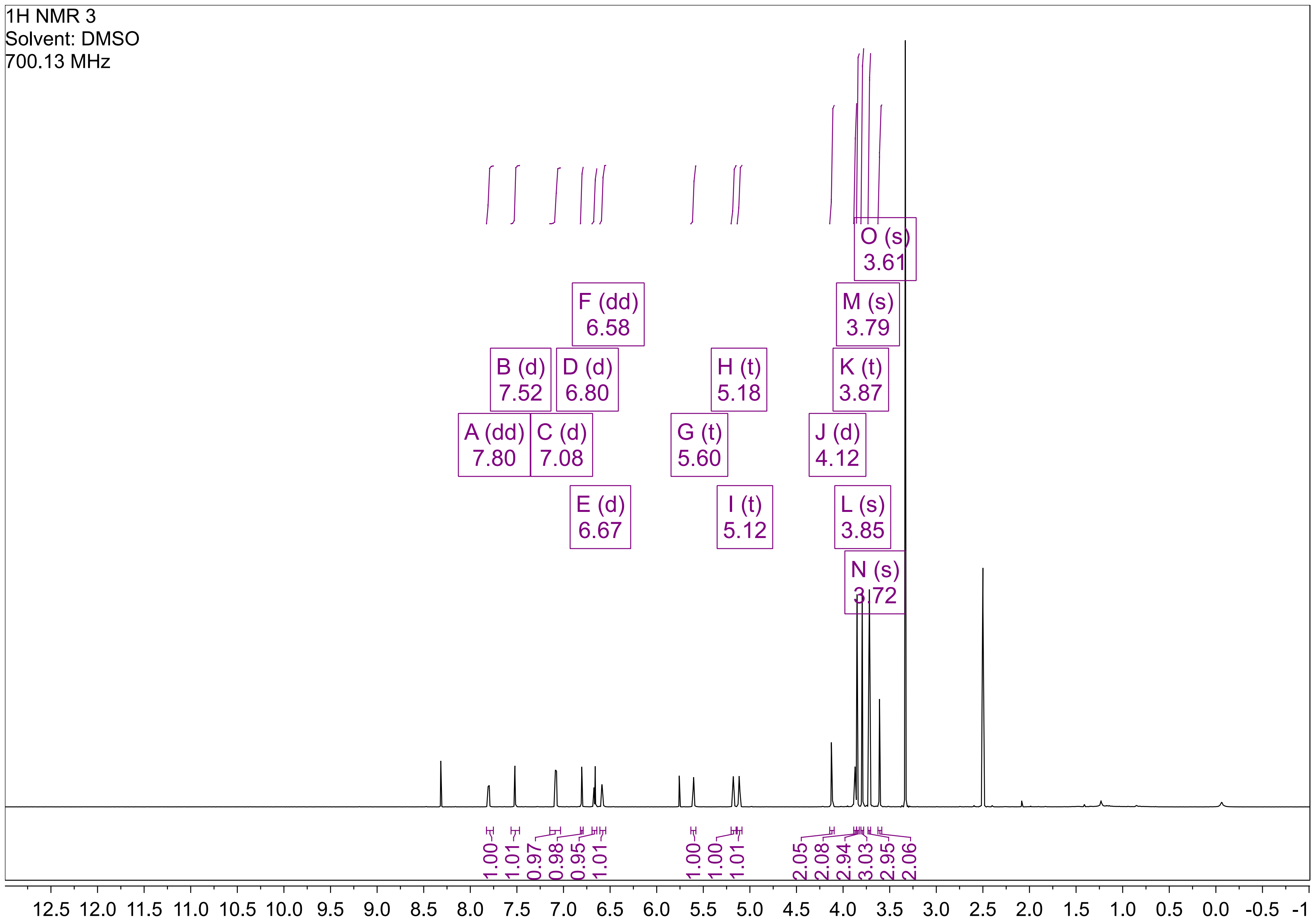
1H NMR 3
Solvent: DMSO
700.13 MHz
O (s) 3.61
M (s) 3.79
F (dd) 6.58
B (d) 7.52
D (d) 6.80
H (t) 5.18
K (t) 3.87
A (dd) 7.80
C (d) 7.08
G (t) 5.60
J (d) 4.12
E (d) 6.67
I (t) 5.12
L (s) 3.85
N (s) 3.72
1.00
1.01
0.97
0.98
0.95
1.01
1.00
1.00
1.01
2.05
2.08
2.94
3.03
2.95
2.06
12.5 12.0 11.5 11.0 10.5 10.0 9.5 9.0 8.5 8.0 7.5 7.0 6.5 6.0 5.5 5.0 4.5 4.0 3.5 3.0 2.5 2.0 1.5 1.0 0.5 0.0 -0.5 -1

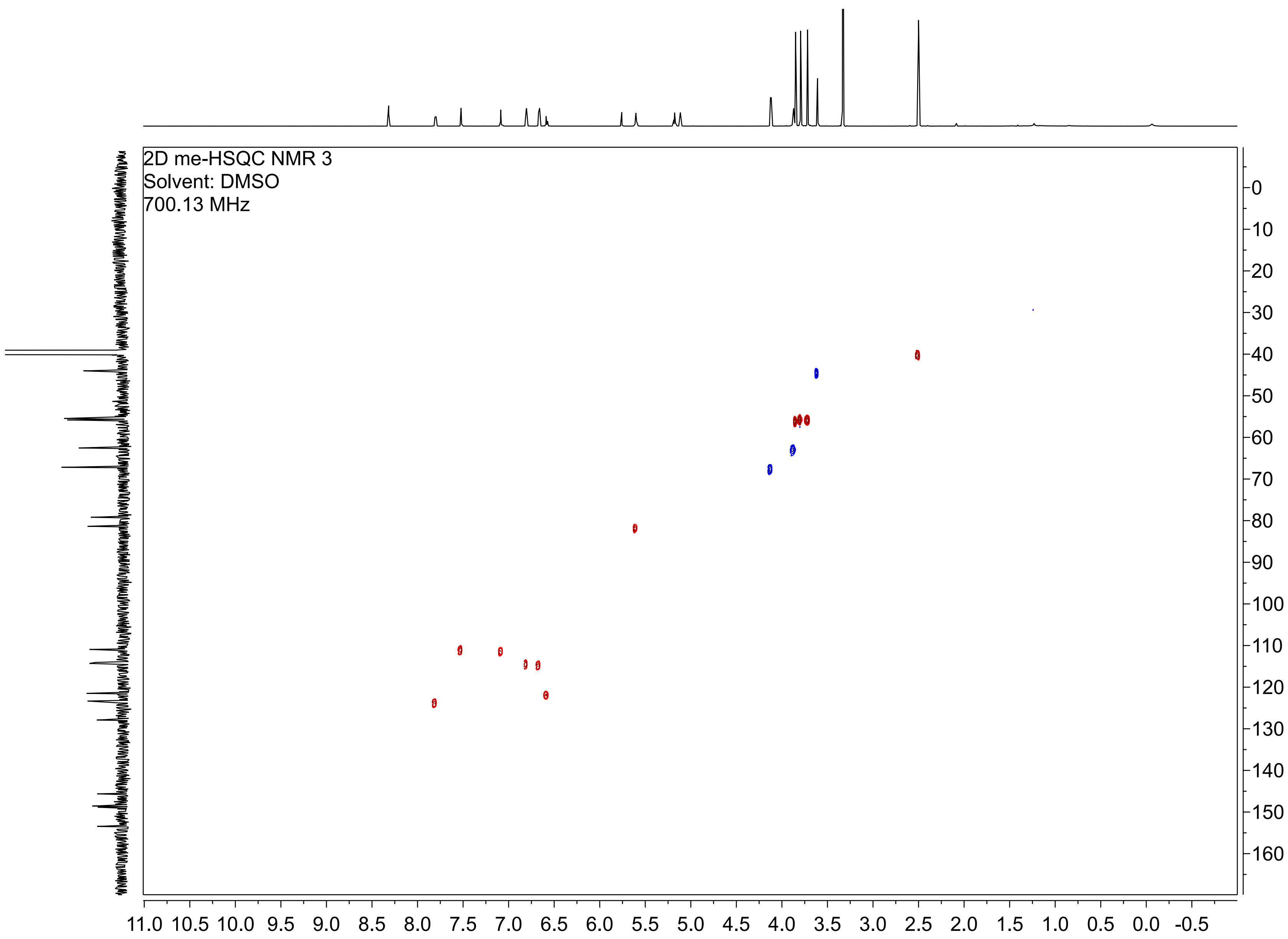

2D me-HSQC NMR 3
Solvent: DMSO
700.13 MHz
0
10
20
30
40
50
60
70
80
90
100
110
120
130
140
150
160
11.0 10.5 10.0 9.5 9.0 8.5 8.0 7.5 7.0 6.5 6.0 5.5 5.0 4.5 4.0 3.5 3.0 2.5 2.0 1.5 1.0 0.5 0.0 -0.5

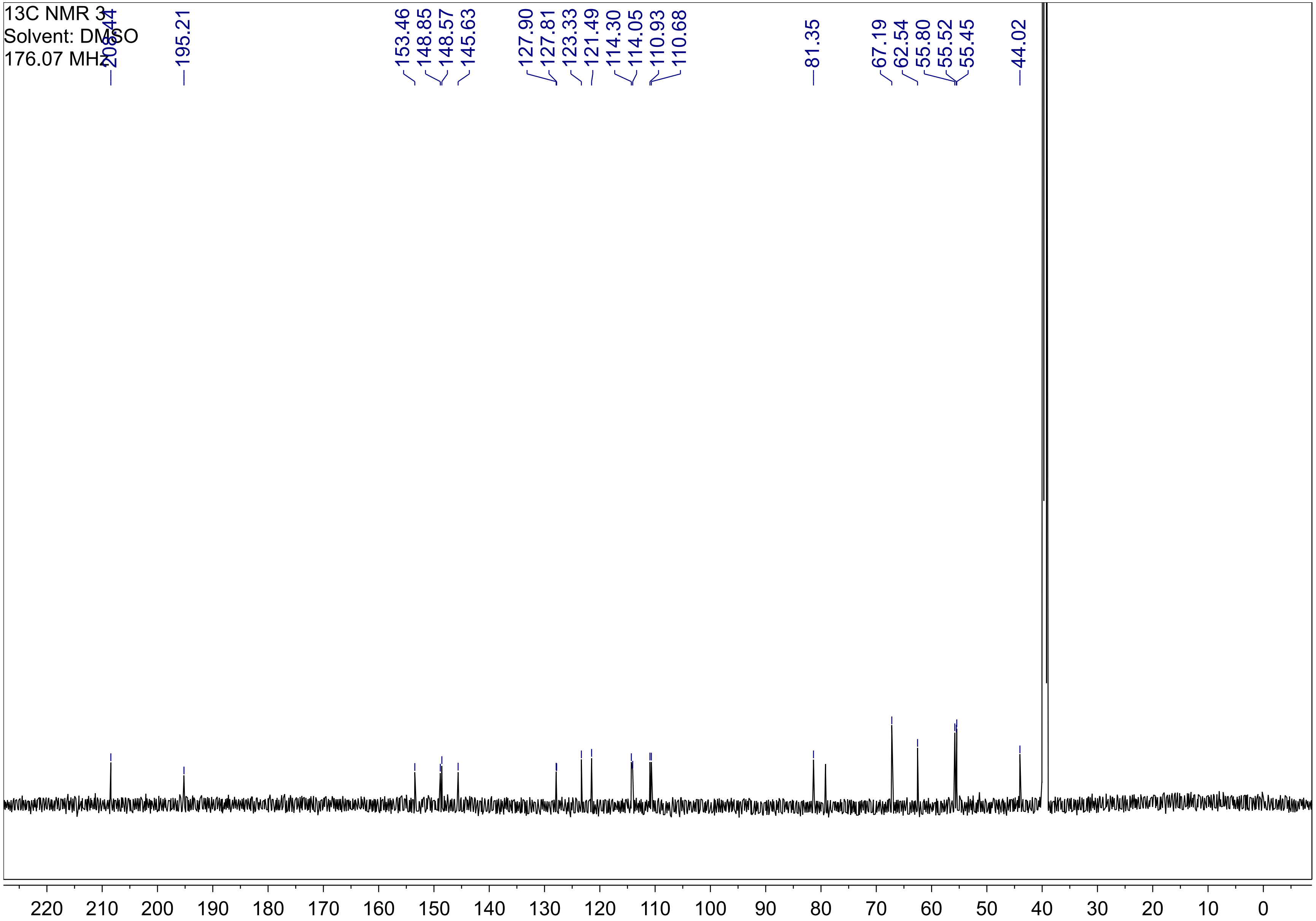
13C NMR 3
Solvent: DMSO
176.07 MHz
208.44
195.21
153.46
148.85
148.57
145.63
127.90
127.81
123.33
121.49
114.30
114.05
110.93
110.68
81.35
67.19
62.54
55.80
55.52
55.45
44.02
220 210 200 190 180 170 160 150 140 130 120 110 100 90 80 70 60 50 40 30 20 10 0

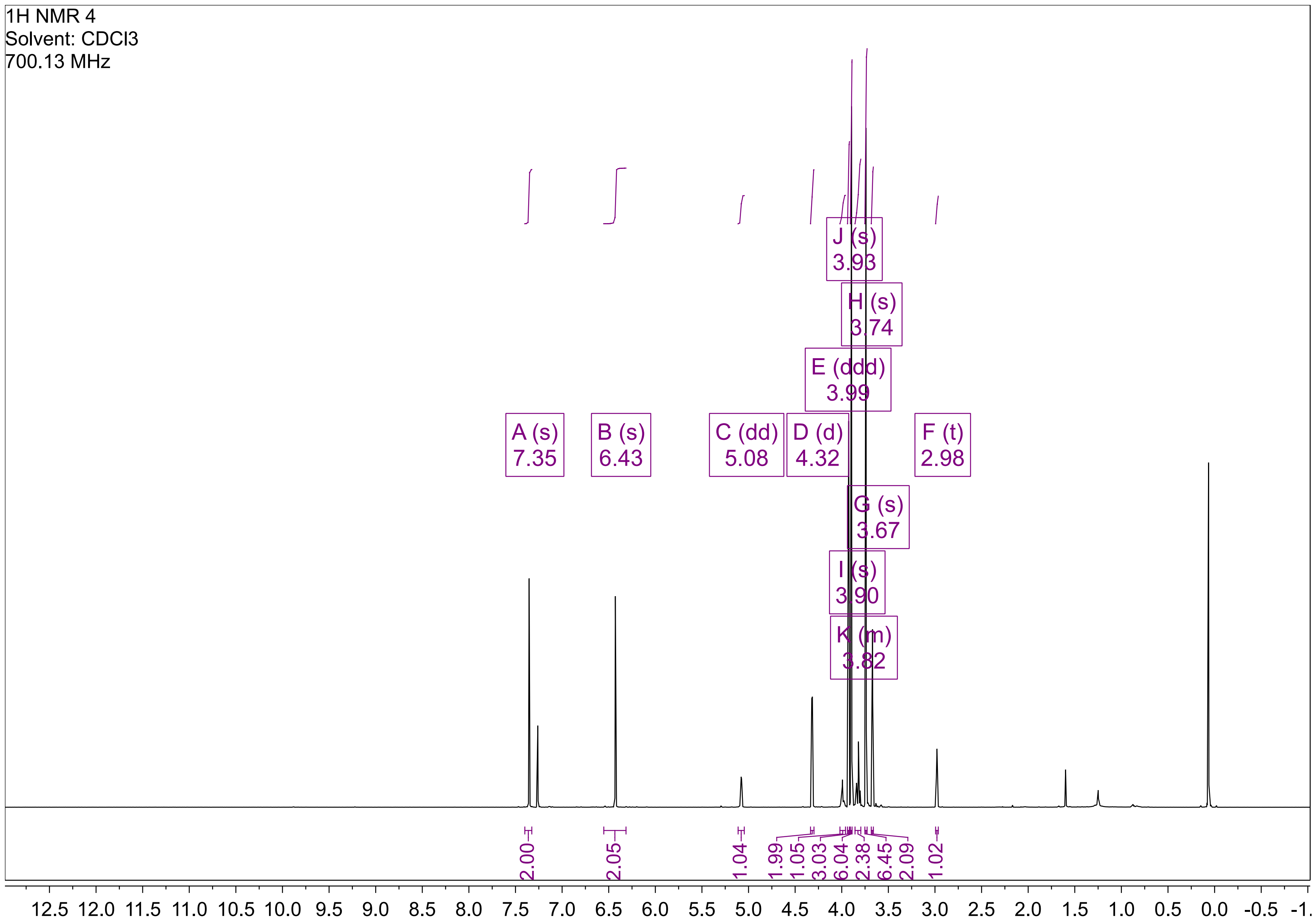
1H NMR 4
Solvent: CDCl3
700.13 MHz
A (s) 7.35
B (s) 6.43
C (dd) 5.08
D (d) 4.32
E (ddd) 3.99
F (t) 2.98
G (s) 3.67
H (s) 3.74
I (s) 3.90
J (s) 3.93
K (m) 3.82
2.00
2.05
1.04
1.99
1.05
3.03
6.04
2.38
6.45
2.09
1.02
12.5 12.0 11.5 11.0 10.5 10.0 9.5 9.0 8.5 8.0 7.5 7.0 6.5 6.0 5.5 5.0 4.5 4.0 3.5 3.0 2.5 2.0 1.5 1.0 0.5 0.0 -0.5 -1.

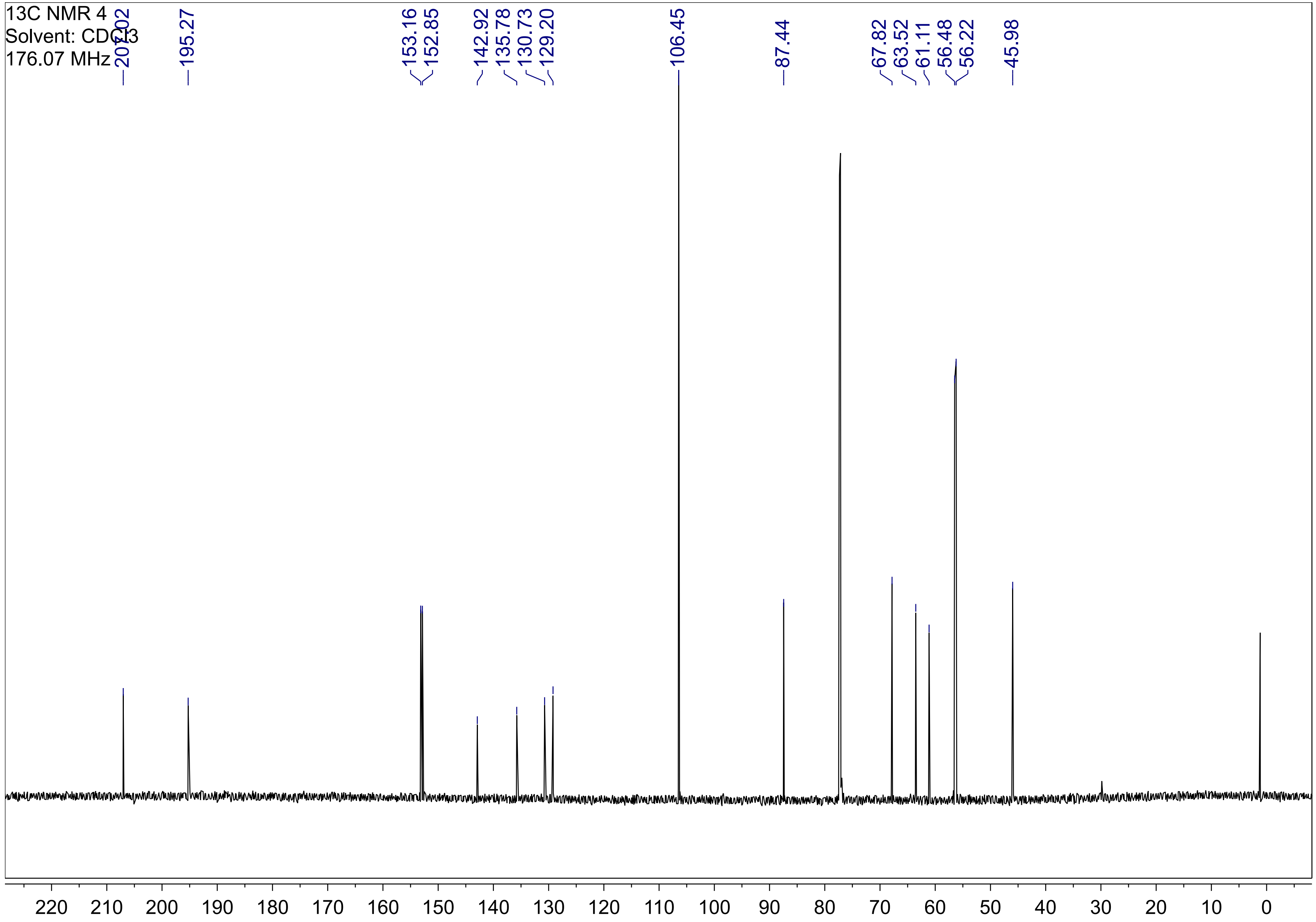
13C NMR 4
Solvent: CDCl3
176.07 MHz
207.02
195.27
153.16
152.85
142.92
135.78
130.73
129.20
106.45
87.44
67.82
63.52
61.11
56.48
56.22
45.98
220 210 200 190 180 170 160 150 140 130 120 110 100 90 80 70 60 50 40 30 20 10 0

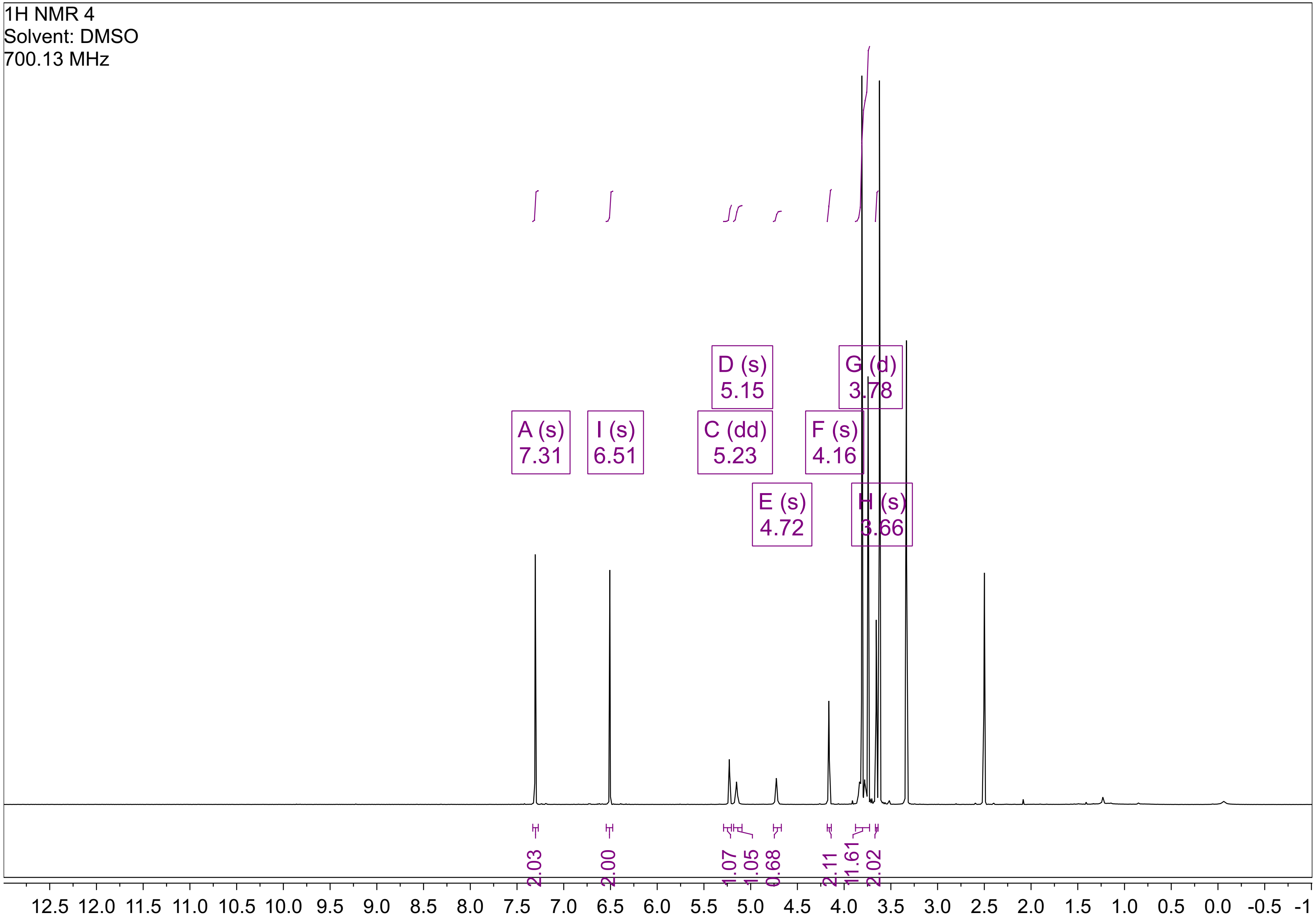
1H NMR 4
Solvent: DMSO
700.13 MHz
A (s)
7.31
I (s)
6.51
D (s)
5.15
C (dd)
5.23
E (s)
4.72
F (s)
4.16
G (d)
3.78
H (s)
3.66
2.03
2.00
1.07
1.05
0.68
2.11
11.61
2.02
12.5 12.0 11.5 11.0 10.5 10.0 9.5 9.0 8.5 8.0 7.5 7.0 6.5 6.0 5.5 5.0 4.5 4.0 3.5 3.0 2.5 2.0 1.5 1.0 0.5 0.0 -0.5 -1

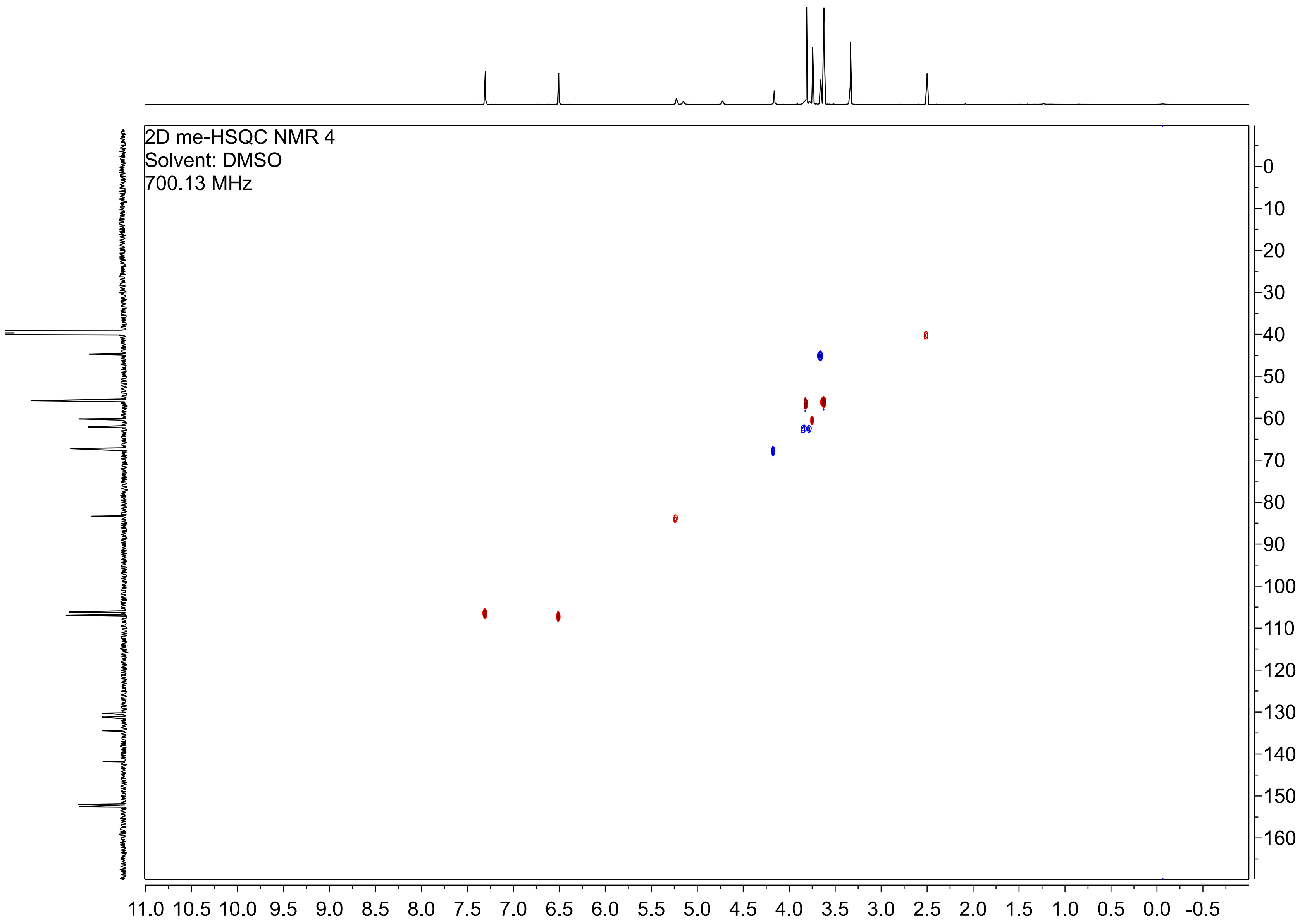

2D me-HSQC NMR 4
Solvent: DMSO
700.13 MHz
0
10
20
30
40
50
60
70
80
90
100
110
120
130
140
150
160
11.0 10.5 10.0 9.5 9.0 8.5 8.0 7.5 7.0 6.5 6.0 5.5 5.0 4.5 4.0 3.5 3.0 2.5 2.0 1.5 1.0 0.5 0.0 -0.5

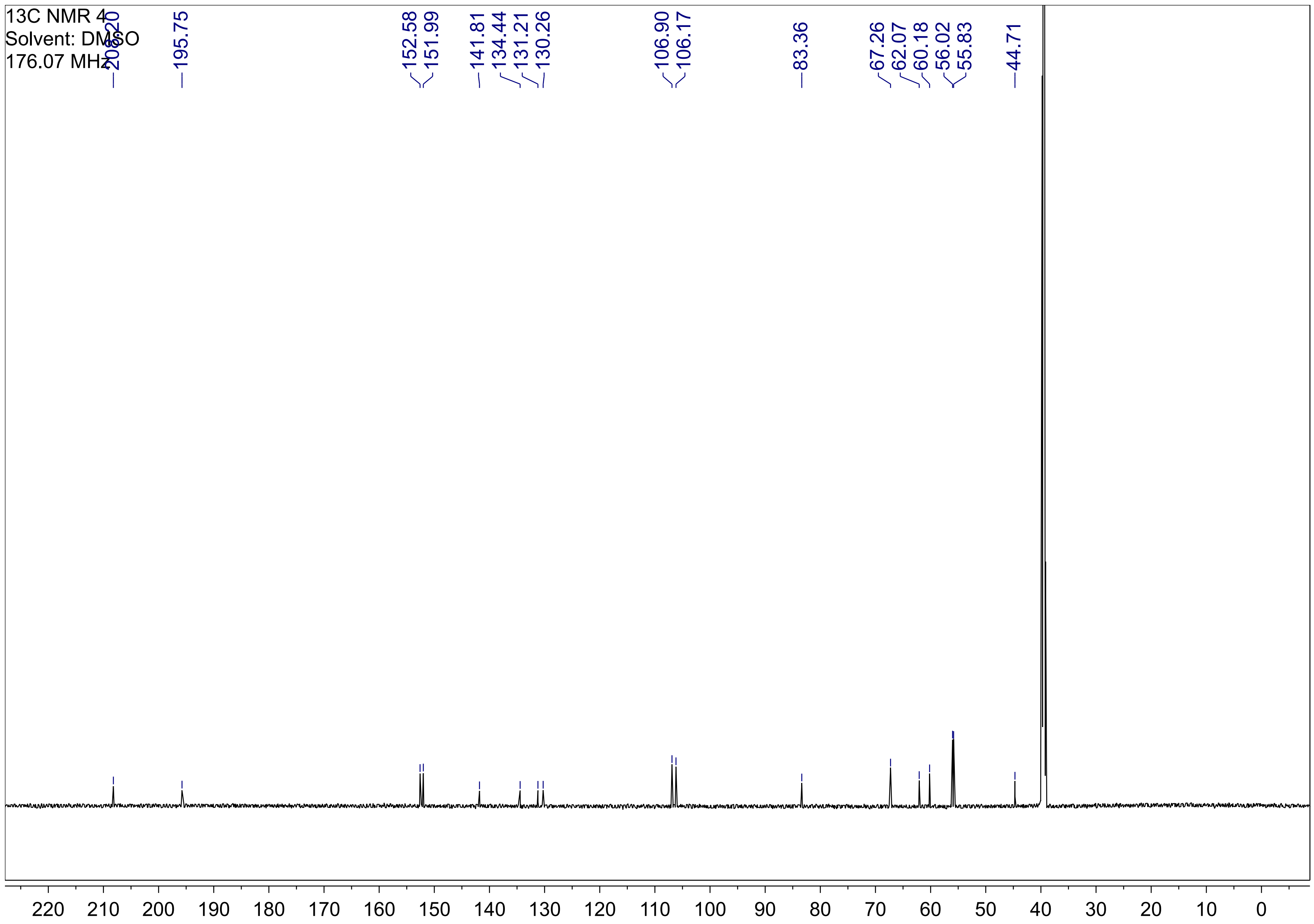
13C NMR 4
Solvent: DMSO
176.07 MHz
208.20
195.75
152.58
151.99
141.81
134.44
131.21
130.26
106.90
106.17
83.36
67.26
62.07
60.18
56.02
55.83
44.71
220 210 200 190 180 170 160 150 140 130 120 110 100 90 80 70 60 50 40 30 20 10 0
